# Design, synthesis and biological evaluation of arylsulfonamides as ADAMTS7 inhibitors†

**DOI:** 10.1039/d4md00149d

**Published:** 2024-06-19

**Authors:** Doretta Cuffaro, Tina Burkhard, Bianca Laura Bernardoni, Riccardo Di Leo, Xiaohan Zhang, Salvatore Galati, Tiziano Tuccinardi, Marco Macchia, Armando Rossello, Salvatore Santamaria, Rens de Groot, Elisa Nuti

**Affiliations:** a Department of Pharmacy, University of Pisa Via Bonanno 6 56126 Pisa Italy elisa.nuti@unipi.it +39 0502219551; b Department of Biochemical and Physiological Sciences, School of Biosciences, Faculty of Health and Medical Sciences, University of Surrey Edward Jenner Building Guildford GU2 7XH UK s.santamaria@surrey.ac.uk; c Institute of Cardiovascular Science, University College London 51 Chenies Mews London WC1E 6HX UK R.deGroot@ucl.ac.uk +44 (0) 20 3108 1423

## Abstract

The proteolytic activity of the enzyme ADAMTS7 was recently shown to enhance the progression of atherosclerosis, in line with human genetic findings suggesting that ADAMTS7 has a role in the pathophysiology of coronary heart disease. Targeting the active site of ADAMTS7 with a small molecule inhibitor, therefore, has therapeutic potential. Here, we report the design and synthesis of a novel hydroxamate-based arylsulfonamide that is a potent and selective ADAMTS7 inhibitor. *In silico* studies guided the hit optimization process aiming to improve selectivity of the previously reported (non-selective) inhibitor EDV33. Our lead compound is a *p*-trifluoromethyl biphenyl sulfonamide, which displayed a 12-fold selectivity for ADAMTS7 (*K*_i_ = 9 nM) over ADAMTS5 (*K*_i_ = 110 nM) and an 8-fold increase in inhibition of ADAMTS7 compared to EDV33 (*K*_i_ = 70 nM). The substitutions switched selectivity and produced a new potent ADAMTS7 inhibitor that can be taken forward for further characterisation.

## Abstract 2

Starting from the ADAMTS5/ADAMTS7 inhibitor EDV33, a structure-based optimization led to the lead compound 3a, with nanomolar activity against ADAMTS7 and good selectivity over ADAMTS5.

## Graphical abstract

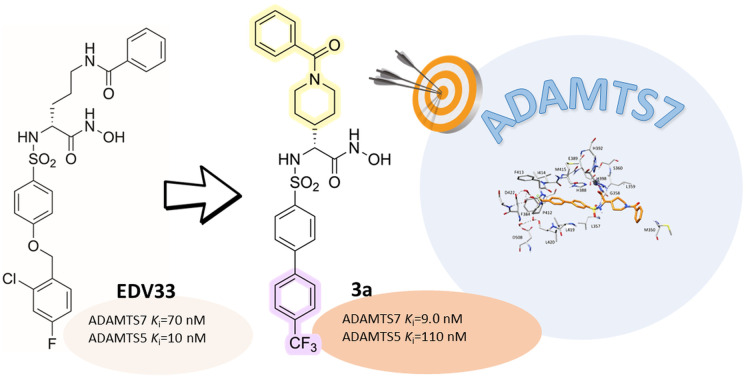

## 1. Introduction

The metalloprotease A disintegrin and metalloproteinase with thrombospondin motif (ADAMTS)7 is a therapeutic target in coronary heart disease (CHD).^1,2^ The *ADAMTS7* locus is consistently associated with CHD and associated phenotypes in Genome Wide Association Studies.^3–8^ The pathophysiology of CHD is complex and involves both atherosclerosis and vascular remodeling.^9^ In *Ldlr*^*−/−*^ and *Apoe*^*−/−*^ mice, deletion of the *ADAMTS7* gene reduced atherosclerosis.^10^ Another study confirmed that this effect was mediated by the proteolytic activity of ADAMTS7,^11^ suggesting that a small molecule inhibitor that targets the active site of the enzyme could also slow down the progression of atherosclerosis. The molecular and cellular mechanisms that mediate the effect of ADAMTS7 on atherosclerosis and CHD are not entirely clear. What is known is that ADAMTS7 can affect both re-reendothelialization of damaged/diseased arteries^12^ and vascular smooth muscle cell behavior,^10,11^ two cell types central to the disease process.

Overall, strong evidence suggests ADAMTS7 as a promising pharmacological target to treat diseases in which atherosclerosis and vascular smooth muscle cells play a central role.^13–15^ The inhibition of ADAMTS7, achieved by using compounds able to chelate the catalytic zinc ion, represents a promising novel approach for CHD and atherosclerosis treatment. However, so far just a first series of hydantoin-based selective ADAMTS7 inhibitors have been reported^16^ very recently by Bayer's researchers, as high structural and functional similarities among ADAMTS proteases and other members of the metzincin superfamily make the design of selective zinc-chelating inhibitors a particularly challenging task.^17,18^

The ADAMTS proteases play diverse roles in connective tissue organization, coagulation, inflammation, arthritis, angiogenesis and cell migration.^19,20^ With the notable exception of ADAMTS13, they are involved in the remodeling of the extracellular matrix or in modulating cell–matrix interactions, and their emerging role in cardiovascular diseases recently gained a considerable attention.^21–24^ The ADAMTS family comprises 19 secreted zinc metalloproteinases and 7 ADAMTS-like proteins (characterized by the lack of the catalytic domain). Their expression is finely regulated at different levels (transcription, activation, inhibition) while dysregulated profiles have been found in many pathological conditions.^25^ From the N-terminus, their general structure consists of the metalloproteinase catalytic domain, and ancillary domains such as a disintegrin-like domain, a central thrombospondin-type I motif (TSR), a cysteine-rich domain, a spacer domain, and, with the exception of ADAMTS4, a various number of TSRs.^20,26^ The active site, responsible for proteolytic activity and common to all the ADAMTS family members, is characterized by the presence of three histidine residues coordinating a Zn^2+^ ion, a glutamic acid residue and three structural Ca^2+^ ions.^27^

**Fig. 1:**
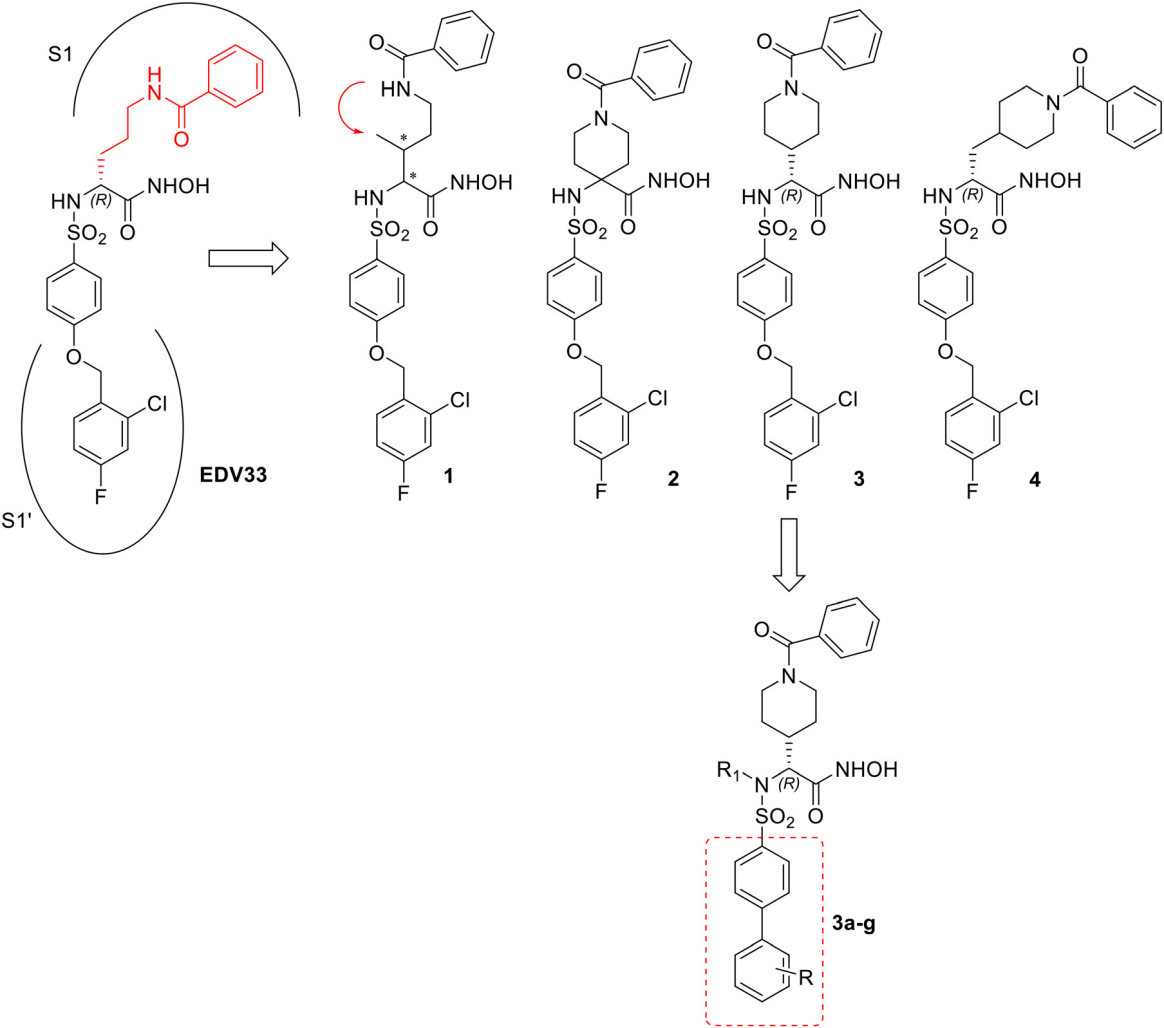
Progression of the structure activity relationship (SAR) study.

Broad-spectrum inhibition of these proteases could cause off-target toxicity as not all ADAMTSs are involved in cardiovascular diseases. ADAMTS5 and ADAMTS4 (aggrecanases) have emerged as targets in osteoarthritis^28,29^ and ADAMTS1 has been proposed as a target in breast cancer therapy,^19^ but ADAMTS7 is comparatively much less characterized. Just very recently we developed the first fluorescence resonance energy transfer (FRET) substrate for ADAMTS7 based on the cleavage sites we identified in the protein substrate latent TGF-β binding protein 4 (LTBP4).^30^ The use of this FRET peptide represents a rapid and quantitative way to perform high-throughput screening (HTS) of large molecule libraries of potential inhibitors. To validate the suitability of our FRET assay to screen and identify ADAMTS7 inhibitors, we characterized the inhibitory activity of the sulfonamido-based hydroxamic acid EDV33.^30^ We showed that while potently inhibiting its target ADAMTS5, EDV33 retained nanomolar activity against ADAMTS7 (*K*_i_ 70 nM). ADAMTS5 is considered as an anti-target in CHD (reviewed in Santamaria 2020 (ref. 31)), since recombinant ADAMT5 has been shown to release low density lipoproteins (LDLs) from human atherosclerotic lesions and *ApoE*^*−/−*^ knockout mice have higher levels of ADAMTS5 and its proteoglycan (proatherogenic) substrates.^32^ Here, we report the design and synthesis of a series of novel hydroxamate-based arylsulfonamides which represent a first step towards obtaining selective ADAMTS7 inhibitors. *In silico* studies guided the hit optimization process (Fig. 1) aiming to improve EDV33 selectivity for ADAMTS7 over ADAMTS5.

Despite the debate over the feasibility of using hydroxamate as a zinc-binding group (ZBG)^33^ due to known plasma stability issues, we chose to maintain this moiety at least in the first series of compounds to privilege *in vitro* efficacy. Moreover, a recent study by Hermant *et al.*^34^ showed that a sulfonamide scaffold with a bulky substituent in α position to the hydroxamate could be beneficial for plasma stability.

The first modifications focused on the linear benzamide chain (P1 group) in α position to the hydroxamic acid, that was replaced by a constrained analogue such as a *N*-benzamido-piperidine ring positioned at different lengths from the chiral center (compounds 2–4, Fig. 1). On the basis of preliminary activity data, a second series of biphenylsulfonamide derivatives of compound 3 bearing a *N*-benzamido-piperidinyl substituent in P1 (compounds 3a–g, Fig. 1) was then designed in order to improve selectivity over ADAMTS5. The percentage of amino acid conservation in the active site of ADAMTS5 and ADAMTS7 is only 43%, indicating the presence of non-conserved residues that could provide selective interactions for novel ligands. More in detail, the presence of non-conserved residues that form the S1′ pocket suggested that changes in the portion of the ligand that is placed at this position could contribute to selectivity. These non-conserved residues in ADAMTS5, I446, P451, L534, correspond to D422, T427 and D508 in the active site of ADAMTS7. This difference makes the ADAMTS7 pocket more suitable to allocate bulky and linear substituents that could not fit within the ADAMTS5 S1′ pocket. On this basis, we designed and synthesized biphenyl derivatives 3a–g bearing rigid and linear substituents in P1′ that were screened against recombinant human ADAMTS7 in order to select the P1′ substituent able to give the best selectivity profile for the target enzyme over other metzincins. Finally, the ability of the best compound to block ADAMTS7 proteolytic activity on a protein substrate (LTBP4S-A) was verified *in vitro* by a western blot assay.

## 2. Results and discussion

### 2.1. Chemistry

**Scheme 1:**
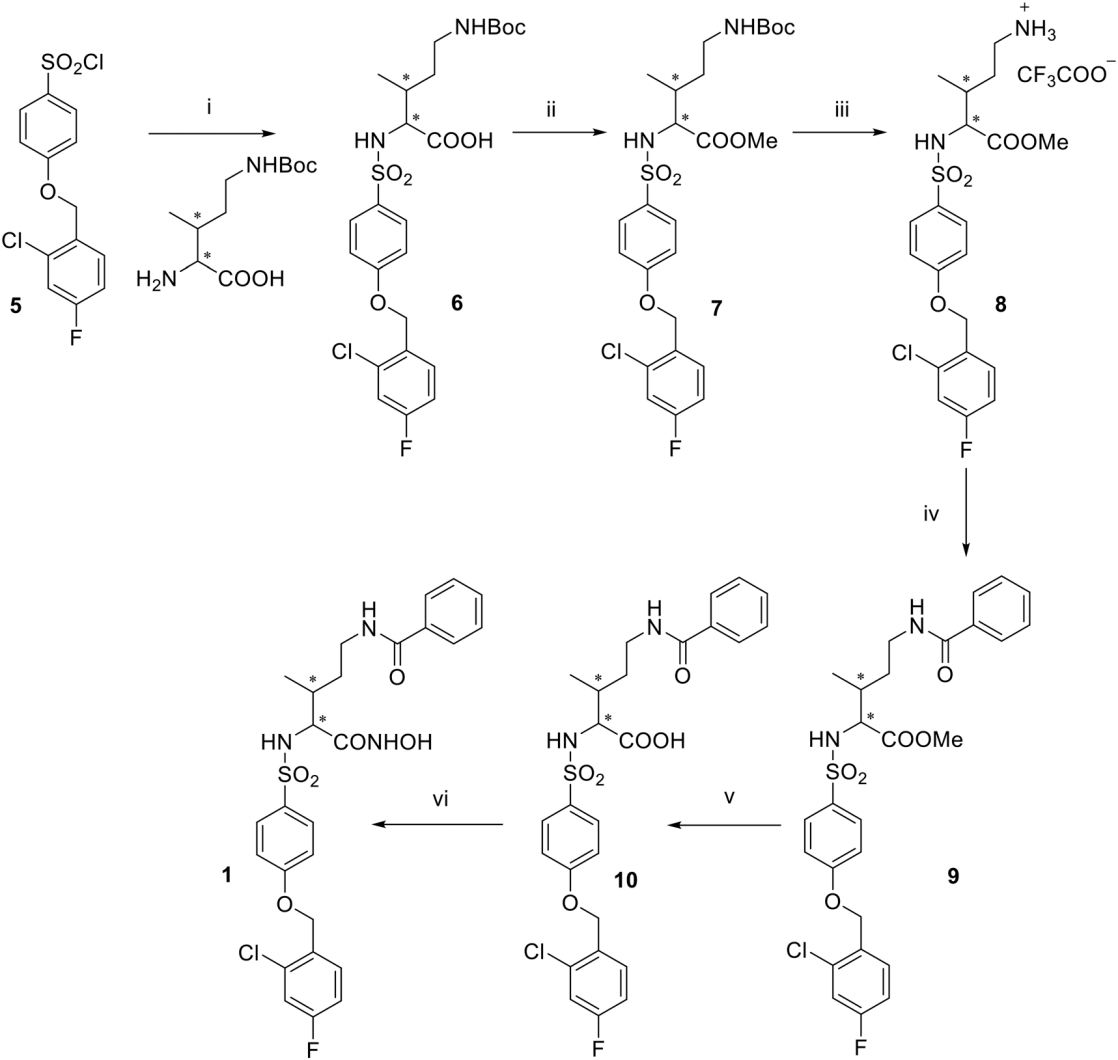
Reagents and conditions: (i) Et_3_N, H_2_O/dioxane 1 : 1, rt, 16 h, 79%; (ii) CH_3_I, K_2_CO_3_, DMF dry, rt, 3 h, quantitative; (iii) TFA, DCM dry, rt, 3 h, 91%; (iv) benzoyl chloride, DIPEA, DMF dry, rt, 3 h, 99%; (v) LiOH 1 N, MeOH, 60 °C, 6 h, 75%; (vi) 1. THPONH_2_, EDC, HOBt, NMM, DMF dry, rt, 18 h; 2. HCl 4 N, MeOH/dioxane 1 : 1, rt, 6 h, 16% over two steps.

**Scheme 2:**
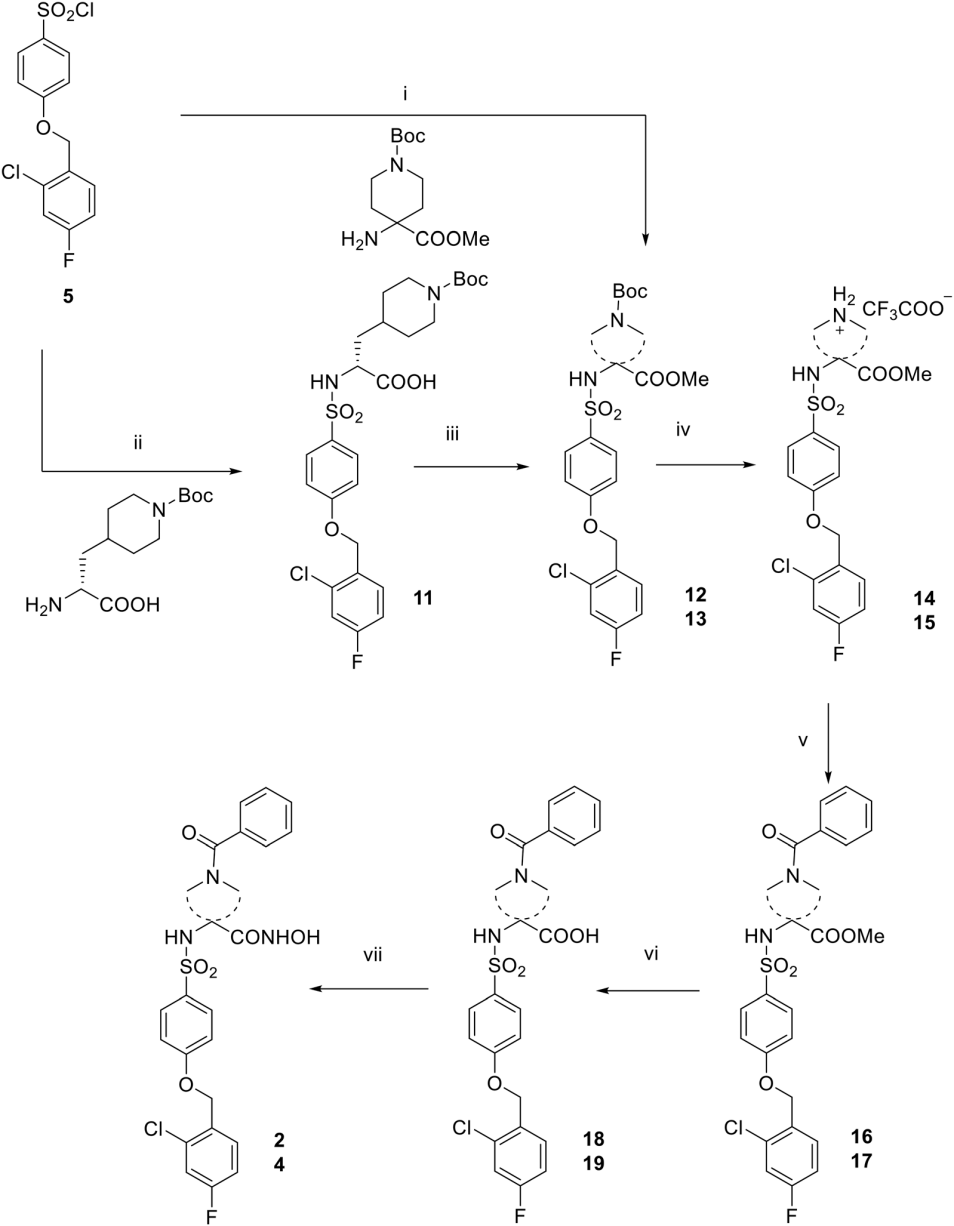
Reagents and conditions: (i) pyridine dry, CHCl_3_ dry, 18 h, rt, 84%; (ii) Et_3_N, H_2_O/dioxane 1 : 1, rt, 18 h, 85%; (iii) CH_3_I, K_2_CO_3_, DMF dry, rt, 3 h, 34%; (iv) TFA, DCM dry, rt, 3 h (14: 92%; 15: quantitative); (v) benzoyl chloride, DIPEA, DMF dry, rt, 3 h (16: 88%; 17: 95%); (vi) LiOH 1 N, 60 °C (18: THF, 5 h, 42%; 19: MeOH, 18 h, quantitative); (vii) 1. THPONH_2_, EDC, HOBt, NMM, DMF dry, 24–48 h; 2. HCl 4 N, MeOH/dioxane 1 : 1, rt, 18 h (2: 20%; 4: 14% over two steps).

Supported by literature and previous data obtained for similar arylsulfonamido-based MMP^35,36^ and ADAM inhibitors^37,38^ over the years, EDV33 and its new optically active derivatives were synthesized as (*R)*-enantiomers. The synthesis of hydroxamates 1, 2 and 4 is reported in Schemes 1 and 2. 4-(2-Chloro-4-fluorobenzyloxy)benzene-1-sulfonyl chloride 5, synthesized as already reported,^39^ was conjugated with the appropriate commercially available amino acid 2-amino-5-((*tert*-butoxycarbonyl)amino)-3-methylpentanoic acid or (*R*)-2-amino-3-(1-(*tert*-butoxycarbonyl)piperidin-4-yl)propanoic acid by reaction in water and dioxane in the presence of triethylamine (Et_3_N) to give sulfonamide carboxylates 6, 11. The latter were then converted into the corresponding methyl esters 7, 13 by S_N_2 reaction with iodomethane, in presence of K_2_CO_3_ in dry *N*,*N*-dimethylformamide (DMF). For the spiro derivative, the methyl ester 12 was directly obtained by reaction of sulfonyl chloride 5 with the commercially available methyl 1-Boc-4-aminopiperidine-4-carboxylate in dry CHCl_3_. The *N*-Boc derivatives 7, 12 and 13 were deprotected by acid hydrolysis using trifluoroacetic acid (TFA) in dry dichloromethane (DCM) at 0 °C to afford trifluoroacetate salts 8, 14 and 15, which were then acylated by reaction with benzoyl chloride in presence of *N,N*-diisopropylethylamine (DIPEA) in dry DMF to give 9, 16 and 17. Basic hydrolysis of esters 9, 16 and 17, conducted with LiOH in methanol or THF, yielded the corresponding carboxylic acids 10, 18 and 19. This hydrolysis was first performed at room temperature for 2 h and then the reaction was heated at 60 °C until disappearance of the starting material on TLC. Finally, a two-steps reaction was carried out starting from the conversion of the carboxylic acids 10, 18 and 19 into the corresponding *O*-(tetrahydropyranyl) intermediates by condensation with *O*-(tetrahydropyranyl)hydroxylamine (THPONH_2_), using *N*-(3-dimethylaminopropyl)-*N′*-ethylcarbodiimide hydrochloride (EDC) as condensing agent, *N*-methylmorpholine (NMM) and 1-hydroxybenzotriazole (HOBt) in dry DMF. Then, the tetrahydropyran protection was removed by acid hydrolysis (4 N HCl), to yield the desired hydroxamic acids 1, 2 and 4. Final compound 1 was obtained as a mixture of diastereomers since the commercial amino acid used as starting compound (2-amino-5-((*tert*-butoxycarbonyl)amino)-3-methylpentanoic acid) contained two stereogenic centers with not defined configuration. The attempt to separate diastereomers by flash chromatography did not get satisfactory results and compound 1 was tested as a mixture of diastereomers.

**Scheme 3:**
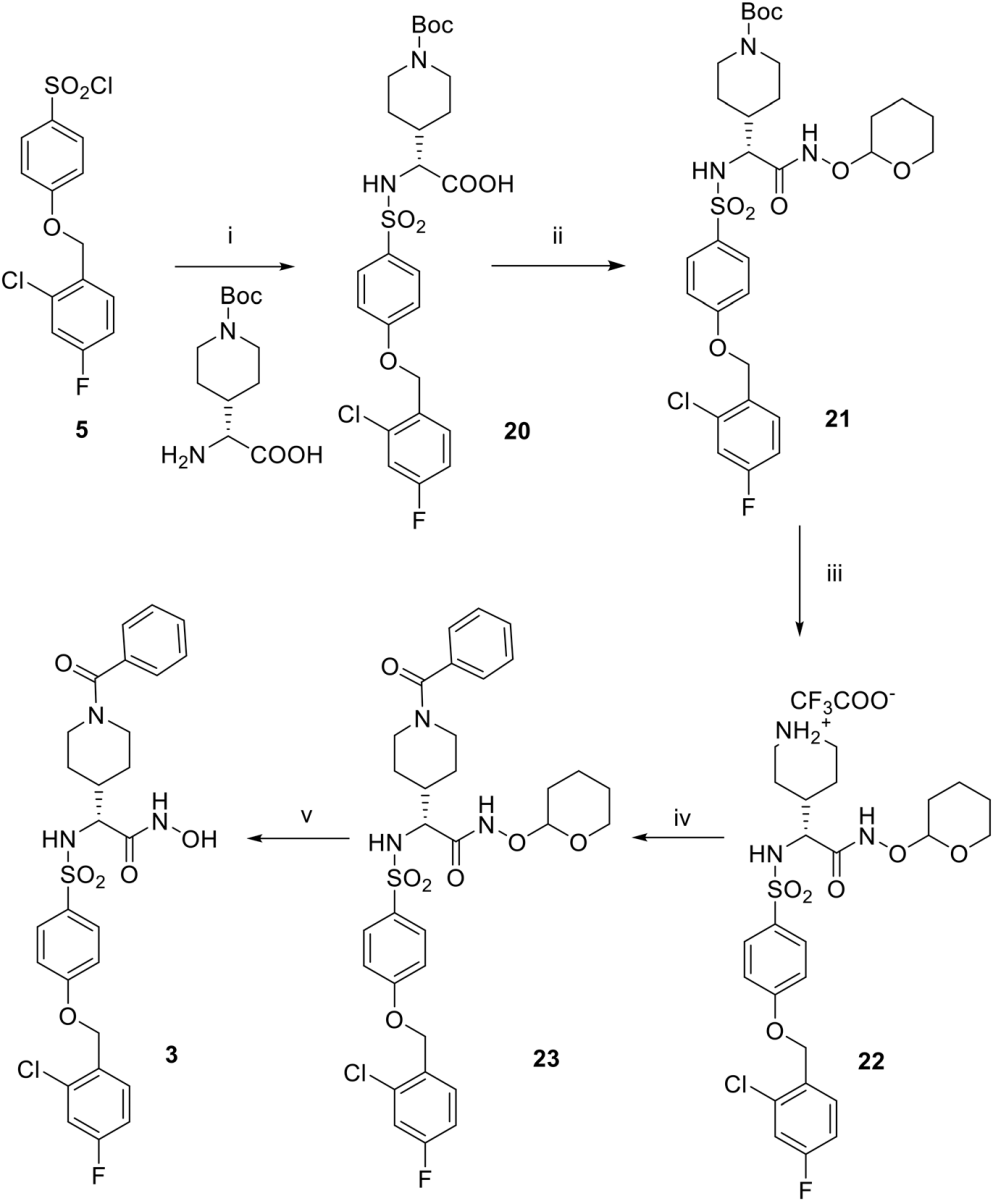
Reagents and conditions: (i) Et_3_N, H_2_O/dioxane 1 : 1, rt, 18 h, 73%; (ii) THPONH_2_, EDC, HOBt, NMM, DMF dry, rt, 18 h, 53%; (iii) TFA, DCM dry, 0 °C, 15 min, 32%; (iv) benzoyl chloride, DIPEA, DMF dry, rt, 3 h, 50%; (v) HCl 4 N, MeOH/dioxane 1 : 1, rt, 18 h, 33%.

The synthesis of 3 has been conducted as reported in Scheme 3. To give sulfonamide 20, the sulfonyl chloride 5 was reacted with the commercially available (*R*)-2-amino-2-(1-(*tert*-butoxycarbonyl)piperidin-4-yl)acetic acid in the presence of triethylamine in a mixture of water and dioxane. (*R*)-Carboxylate 20 was then converted into the *O*-THP-protected hydroxamate 21 by condensation with THPONH_2_ in dry DMF. Compound 21 was then subjected to a selective acid hydrolysis in controlled conditions to remove the Boc group and obtain the corresponding trifluoroacetate salt 22. The low yield of this hydrolysis, performed using TFA in DCM at 0 °C, was due to the competitive hydrolysis of THP protection on the hydroxamate function. The secondary piperidine amine 22 was then acylated by reaction with benzoyl chloride under basic conditions using DIPEA in dry DMF to give benzoyl derivative 23. Finally, the THP protection of 23 was removed by acid cleavage performed with HCl 4 N in dioxane and methanol to give the desired (*R*)-hydroxamic acid 3.

**Scheme 4:**
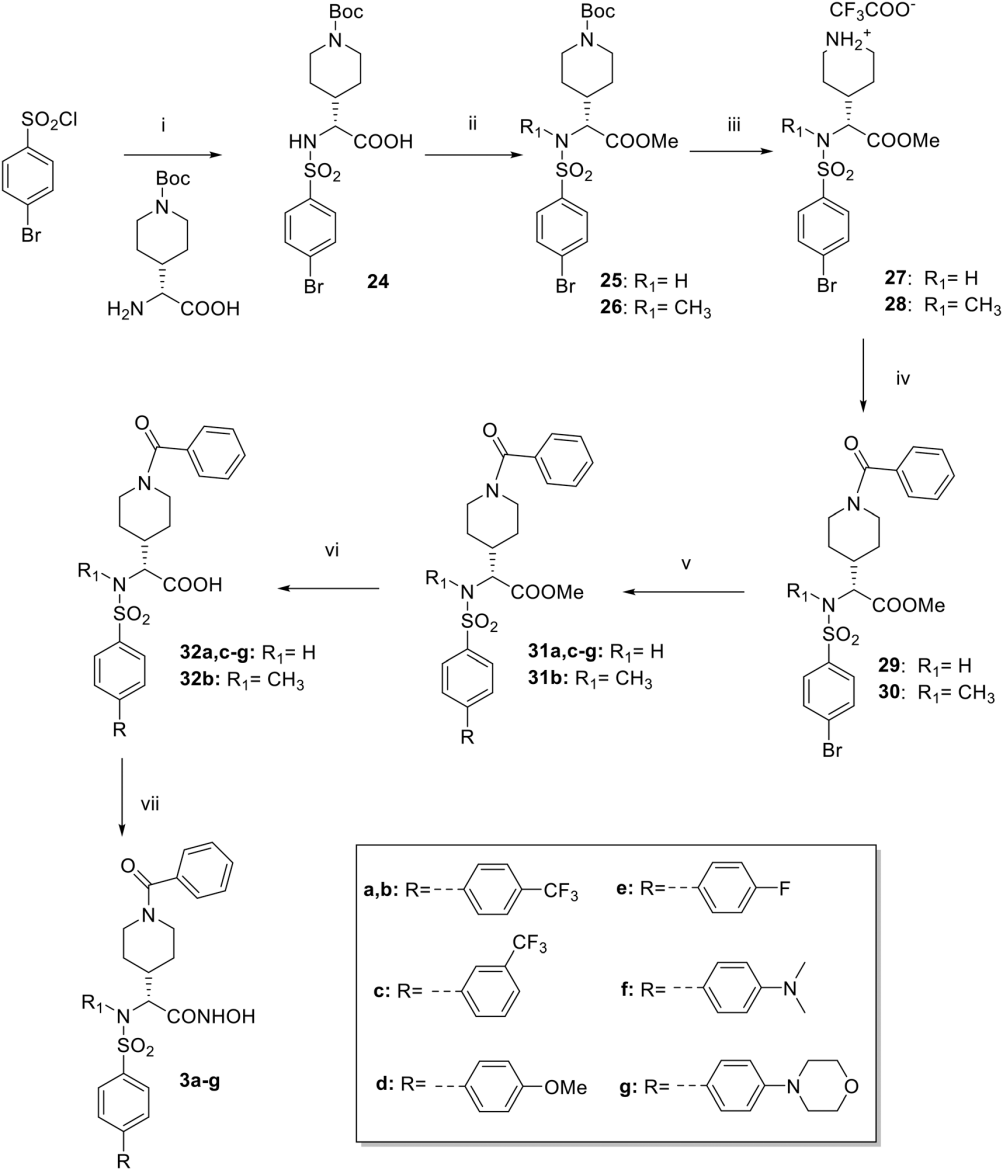
Reagents and conditions: (i) Et_3_N, H_2_O/dioxane 1 : 1, 0 °C, 30 min, 81%; (ii) CH_3_I, K_2_CO_3_, DMF dry, rt, 3 h (25: 53%; 26: 44%); (iii) TFA, DCM dry, 0 °C, 1.5 h, (27: 72%; 28: 95%); (iv) benzoyl chloride, DIPEA, DMF dry, rt, 1–1.5 h, (29: 75%; 30: 95%); (v) RB(OH)_2_, Pd(PPh_3_)_4_, K_3_PO_4_, H_2_O/dioxane 1 : 4,4, 70 °C, 15–30 min, MW (83–98%); (vi) LiOH 1 N, THF, 60 °C, 18 h (54–99%); (vii) 1. THPONH_2_, EDC, HOBt, NMM, DMF dry, rt, 5–48 h; 2. HCl 4 N, MeOH/dioxane 1 : 1, rt, 2–4.5 h (44–73% over two steps).

Compared to Scheme 3, reactions reported in Schemes 1 and 2 showed higher efficiency and reproducibility. For this reason, the synthetic route described in Scheme 4 to prepare compounds 3a–g followed the conditions of Schemes 1 and 2.

The commercially available 4-bromobenzenesulfonyl chloride was firstly conjugated with (*R*)-2-amino-2-(1-(*tert*-butoxycarbonyl)piperidin-4-yl)acetic acid to yield carboxylate 24 that was subsequentially converted into methyl ester 25 by treatment with iodomethane under basic conditions*.* During this reaction, *N*-methylation of the sulfonamido group was also achieved, giving the *N*-methyl ester 26 as a secondary product, which was used for the following reactions after chromatographic separation from 25. Boc deprotection of 25–26 was performed *via* acid hydrolysis to give trifluoroacetate salts 27–28, which were then acylated to obtain benzoyl intermediates 29–30. Different boronic acids (4-(trifluoromethyl)phenylboronic acid, 3-(trifluoromethyl)phenylboronic acid, 4-methoxyphenylboronic acid, (4-fluorophenyl)boronic acid, (4-(dimethylamino)phenyl)boronic acid and (4-morpholinophenyl)boronic acid) were reacted with aryl bromides 29 or 30 in Suzuki cross-coupling conditions to give the corresponding biphenyl derivatives 31a–g. The cross-coupling reactions were carried out using microwave irradiation (70 °C, 15–30 min) in the presence of potassium phosphate tribasic (K_3_PO_4_) and tetrakis(triphenylphosphine)-palladium (Pd(PPh_3_)_4_) in a solution of H_2_O and dioxane (v/v 1 : 4.4). Methyl esters 31a–g were respectively converted into carboxylic acids 32a–g by basic hydrolysis with LiOH in THF at 60 °C. Hydroxamates 3a–g were finally synthesized following the two-step reaction already described in Schemes 1 and 2.

### 2.2. Enzyme inhibitory activity

All newly synthesized final compounds were first screened on recombinant human ADAMTS7 by FRET assay using ATS7FP7 (ref. 30) as a FRET substrate. Inhibition against ADAMTS5 was tested to prove selectivity over an ADAMTS family member representing an anti-target in cardiovascular diseases.^21^ Activity data for compounds 1–4 as compared with EDV33 are reported in Table 1.

**Table 1:** *In vitro* enzymatic^a^ activity (*K*_i_ nM values) of new compounds 1–4 and the reference compound EDV33

Compd	ADAMTS7	ADAMTS5
1	50 ± 20	3.0 ± 1.1
2	380 ± 10	3.0 ± 0.6
3	40 ± 10	6.0 ± 1.0
4	220 ± 80	9.0 ± 1.0
EDV33	70 ± 10	10 ± 0.1

a Data are presented as average ± SEM; *n* = 3.

All 1–4 derivatives (Fig. 1) maintained a 2-chloro-4-fluoro-1-phenoxymethylbenzene sulfonamide as P1′ substituent while the linear benzamide chain in P1 was closed to form a piperidine ring positioned at different distances from the chiral center.

In particular, compound 2 is a piperidine spiro derivative, 3 presents a piperidine ring directly attached to the α position to the ZBG, and 4 bears a piperidine ring separated by a methylene group from the chiral center. Compound 1 presents a P1 substituent that can be considered an intermediate between a linear and a cyclic benzamido-group. Compared to EDV33, such constrained analogues could be endowed with a different orientation in the ADAMTS7 active site.

Compounds 2 and 4 showed a lower potency against ADAMTS7 while piperidinyl derivative 3 showed a ∼2-fold increase of inhibitory activity against ADAMTS7 (*K*_i_ = 40 nM) with respect to EDV33 (Table 1). As expected, compound 1 displayed intermediate activity between EDV33 and its piperidine analogue 3. Of note, all new derivatives retained nanomolar inhibition against ADAMTS5.

To further improve both activity and selectivity, the next step was the modification of P1′ group of piperidinyl derivative 3, which presented the best activity so far. As suggested by molecular docking of EDV33 in the ADAMTS7 active site,^30^ the 2-chloro-4-fluoro-1-phenoxymethylbenzene group present in both EDV33 and 3 was replaced by a more rigid biphenyl moiety to give compounds 3a–g (Fig. 1), bearing different substituents on the distal phenyl ring. In particular, compounds 3a and 3c contained a *p*- and *m*-trifluoromethyl substituent, respectively, and all other compounds were *para*-substituted. Activity data of the biphenylsulfonamides 3a–g are reported in Table 2.

**Table 2:** *In vitro* enzymatic^a^ activity (*K*_i_ nM values) of biphenylsulfonamides 3a–g

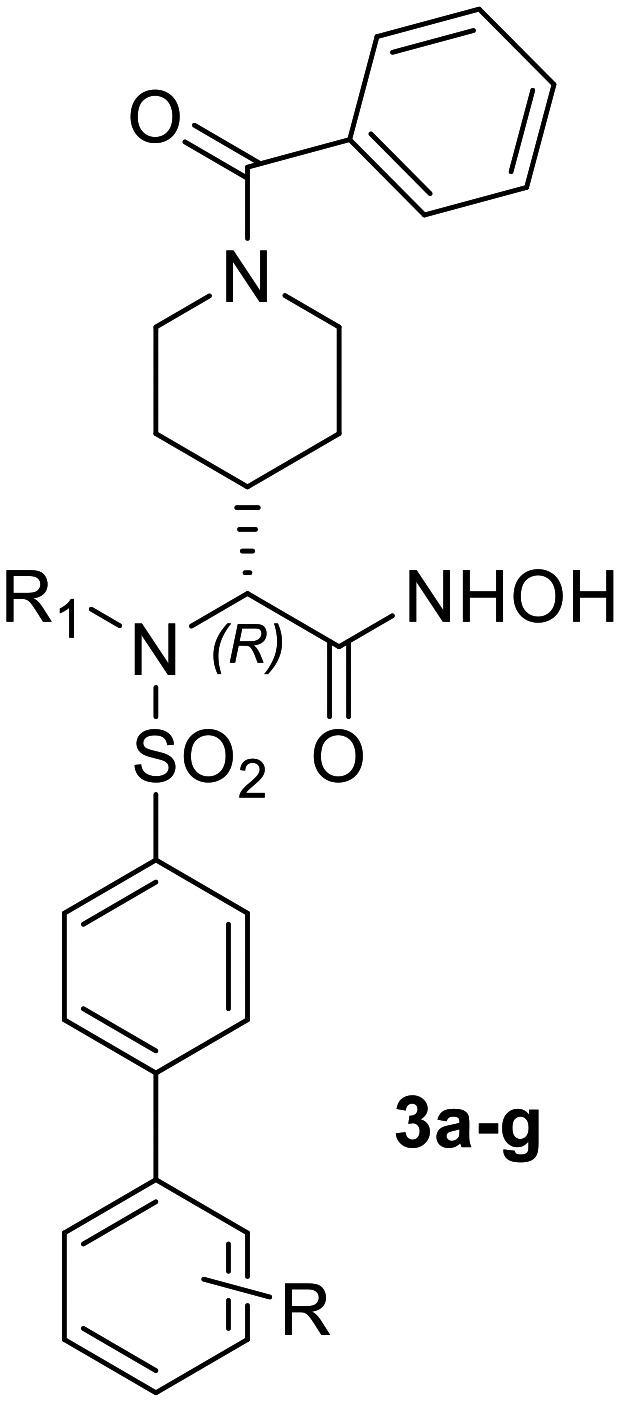				
Compd	R	R_1_	ADAMTS7	ADAMTS5
3a	4-CF_3_	H	9.0 ± 1.0	110 ± 40
3b	4-CF_3_	CH_3_	50 ± 10	90 ± 30
3c	3-CF_3_	H	1000 ± 110	3800 ± 1000
3d	4-OCH_3_	H	40 ± 10	90 ± 20
3e	4-F	H	6.0 ± 1.0	15 ± 4
3f	4-N(CH_3_)_2_	H	550 ± 120	13 000 ± 3200
3g	4-Morpholine	H	940 ± 140	11 000 ± 240

a Data are presented as average ± SEM; *n* = 3.

Overall, the introduction of a rigid and linear biphenylsulfonyl moiety in P1′ led to a shift of selectivity from ADAMTS5 to ADAMTS7. This was a great improvement with respect to EDV33 and previous compounds 1–4. In particular, the *p*-trifluoromethyl biphenyl sulfonamide 3a displayed a 12-fold selectivity for ADAMTS7 (*K*_i_ = 9 nM) over ADAMTS5 (*K*_i_ = 110 nM) and an 8-fold increase in activity against ADAMTS7 compared to EDV33 (*K*_i_ = 70 nM).

The introduction of the same substituent in the *meta*-position of the biphenyl moiety caused a drop of activity for ADAMTS7, with 3c being 100-fold less active than 3a. Also, the *N*-methyl analogue of 3a, compound 3b, showed a decrease of activity and selectivity with respect to 3a. This was not unexpected, considering that similar results were previously obtained for arylsulfonamide ADAM17 inhibitors.^37^ The replacement of the *p*-trifluoromethyl group with a fluorine atom, as recently described for hydantoin-based ADAMTS7 inhibitors,^16,40^ led to a slight increase of inhibitory activity for ADAMTS7 (3e, *K*_i_ = 6.0 nM) which was accompanied by a drop of selectivity over ADAMTS5 (*K*_i_ = 15 nM). Finally, the presence of a basic substituent such as 4-morpholino in 3g or 4-dimethylamino group in 3f led to a decrease in activity against both enzymes, probably due to an excessive bulkiness of the substituents.

The selectivity profile of the most promising derivatives, 3a and 3e, was determined against other metzincins in comparison with the hit compound EDV33. As shown in Table 3, the 4-trifluoromethyl derivative 3a gave the best results in terms of selectivity for ADAMTS7 over the other tested proteases. In particular, 3a showed a 10-fold selectivity for ADAMTS7 over MMP1 and a 14-fold selectivity over ADAM17.

**Table 3:** Inhibition data^a^ (*K*_i_ nM values) of 3a, 3e and the reference compound EDV33 against other metzincins

Compd	ADAMTS7	ADAMTS5	ADAMTS4	ADAMTS1	MMP1	ADAM17
3a	9.0 ± 1.0	110 ± 40	60 ± 20	70 ± 40	90 ± 3	130 ± 10
3e	6.0 ± 1.0	15 ± 4	10 ± 3	30 ± 3	30 ± 1	100 ± 5.0
EDV33	70 ± 14	10 ± 0.1	31 ± 1	2800 ± 400	19 000 ± 1700	8.0 ± 0.5

a Data are presented as average ± SEM; *n* = 3. Data for ADAM17 are presented as IC_50_ values.

To predict the drug-likeness of 3a, an *in silico* calculation of its physicochemical, ADME and toxicological properties was carried out by using MolBook UNIPI Software (Table 4).^41,42^

Chemical descriptors, ADME and toxicological properties predicted for 3a with MolBook UNIPI

**Table:** 

Descriptor	Calculated value
Molecular weight	561.58 Da
H-bond donors	3
H-bond acceptors	5
Log *P*	4.08
TPSA	133.83 Å^2^
PAINS	Passed

a Probability value ranging between 0 and 100.

**Table:** 

Endpoint	Predicted value
Albumin binding	High
BBB permeability	Yes (82^a^)
HIA	Yes (76^a^)
Acute oral toxicity	Not toxic (72^a^)
Androgenicity	Not toxic (93^a^)
Carcinogenicity	Not toxic (64^a^)
Estrogenicity	Not toxic (79^a^)
Eye irritation	Not toxic (80^a^)
Hepatotoxicity	Toxic (56^a^)
Mutagenicity	Not toxic (70^a^)
Skin irritation	Not toxic (93^a^)

According to Lipinski's Rule of Five, compounds with a molecular weight (MW) greater than 500 Da, and a calculated log *P* greater than 5 should guarantee a very low systemic bioavailability. Also molecules that have topological polar surface area (TPSA) > 140 Å^2^ are less likely to reach systemic circulation. In this respect, 3a can be considered a drug-like compound with the exception of the molecular weight threshold (500 Da) since 3a stands at about 561 Da. In addition, *in silico* predictions were performed for the evaluation of ADME and toxicological properties, and compound 3a presented no particular alert albeit a moderate potential hepatotoxicity.

**Fig. 2:**
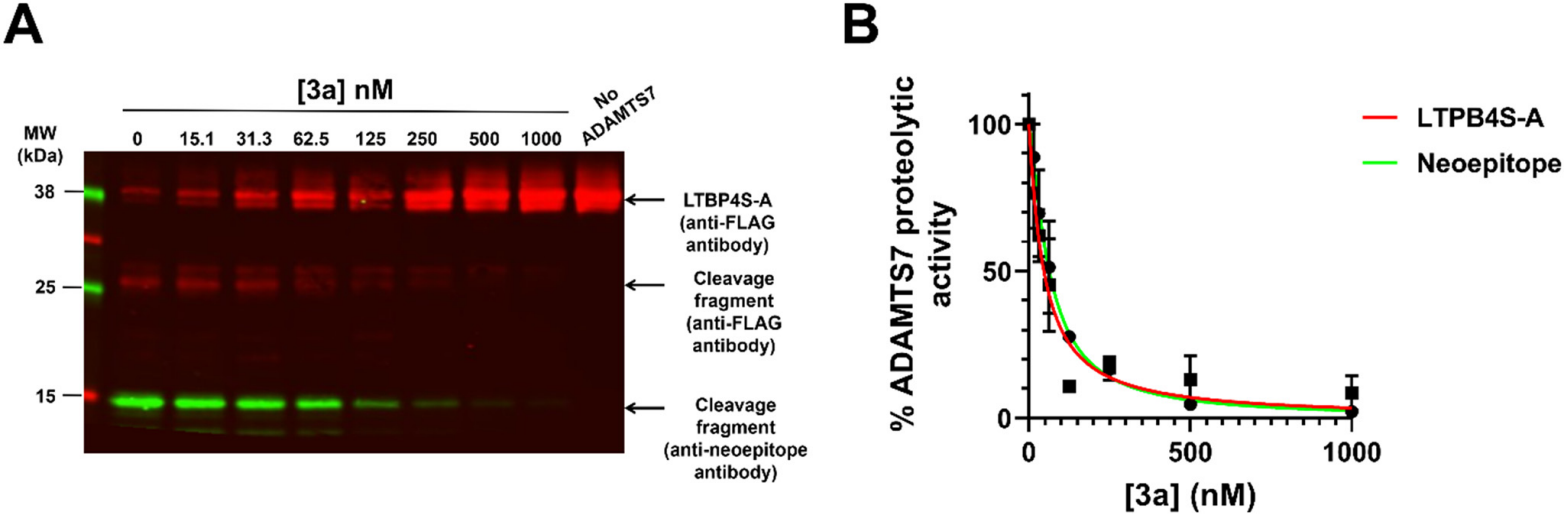
Inhibition of ADAMTS7 proteolytic activity by compound 3a. (A) LTBP4S-A (3.5 μg) was incubated with ADAMTS7 (20 nM) for 24 h at 37 °C and the reactions visualized by immunoblot. Red bands were visualized by a mouse anti-FLAG tag antibody followed by IRDye® 680RD goat anti-mouse IgG. The FLAG tag is at the N-terminus of LTBP4S-A. The green bands were visualized using a custom-made neo-epitope antibody that detects the new N-terminus (AAAPYTV) of the C-terminal cleavage product. Please see the supporting info for the original uncropped image. (B) Densitometric analysis of the C-terminal cleavage product. Data are shown as mean ± SEM (*n* = 3).

To assess the ability of compound 3a to inhibit ADAMTS7 cleavage of a protein substrate, which more closely resembles a translational setting, we deployed the visualization of latent TGFβ binding protein 4 (LTBP4) cleavage fragments by western blot.^43^ The protein fragment used as ADAMTS7 substrate was the N-terminal fragment LTBP4S-A (R27-E356). Visualization of this protein fragment by immunoblot was performed with an antibody against the N-terminal FLAG tag as well as a newly developed antibody against the neo-epitope in the C-terminal cleavage product (^263^AAAPYTV^269^, UniProt ID:Q8N2S1-1). We observed potent inhibition of LTBP4S-A proteolysis by 3a (Fig. 2A), as increasing concentrations of the inhibitor rapidly increased the intensity of the band representing uncleaved LTBP4S-A and reduced the intensity of the bands representing the C-terminal cleavage product detected with the neo-epitope antibody. Both bands were quantified by densitometric analysis. Since we could not determine the Michaelis–Menten constant for cleavage of LTBP4S-A by ADAMTS7 due to the signal saturation intrinsic to immunoblots, IC_50_ values were used instead of *K*_i_ values to evaluate the inhibitory potency of compound 3a. The IC_50_ values determined by densitometric analysis of bands corresponding to uncleaved LTBP4S-A and the neoepitope antibody reactive fragment were similar and within one order of magnitude of the *K*_i_ value determined using the FRET substrate (60 ± 20 and 50 ± 20 nM, respectively) (Fig. 2B). This result suggests that compound 3a is effective in inhibiting ADAMTS7 cleavage of its protein substrates.

### 2.3. Molecular modeling

With the aim to provide a mechanistic understanding of the interactions between compound 3a and the target protease ADAMTS7 and shed light on its selectivity, molecular modeling studies including a robust docking procedure, followed by molecular dynamics (MD) simulations in an explicit water environment, were carried out.

**Fig. 3:**
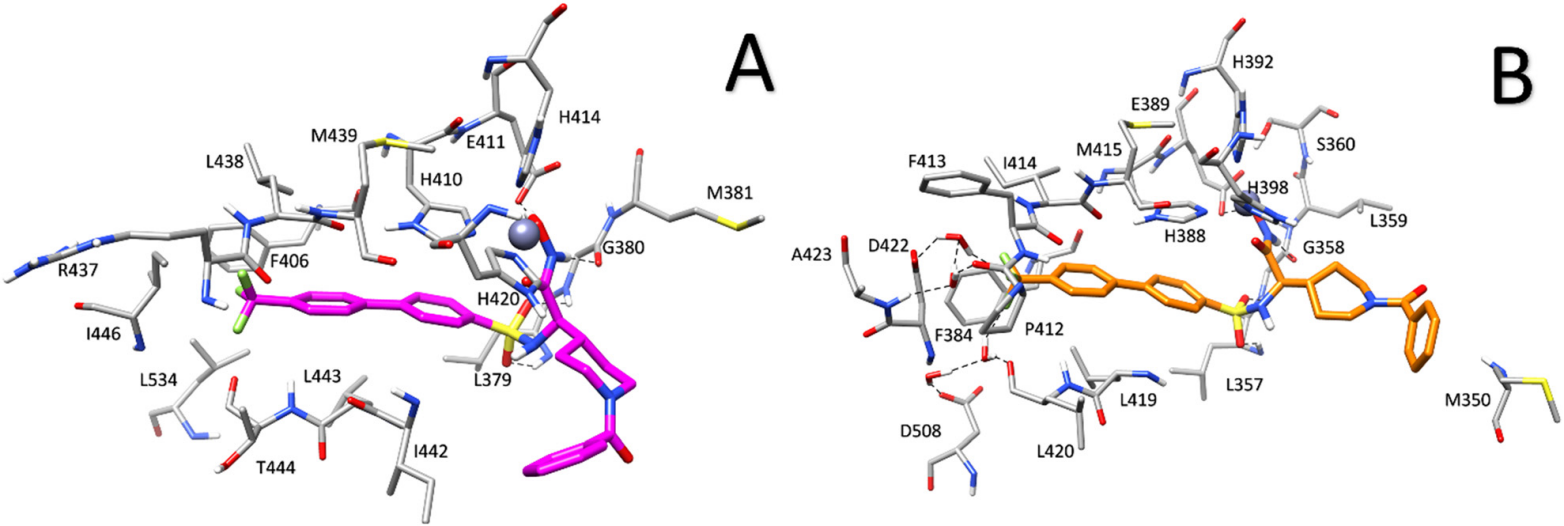
Results of MD simulation and proposed binding mode of 3a in ADAMTS5 (A) and ADAMTS7 (B). 3a is shown with magenta carbon atoms in the ADAMTS5 model (A) and with orange carbon atoms in the ADAMTS7 (B) model.

To date, no experimental 3D structures of the metalloproteinase domain of ADAMTS7 have been reported; therefore, a homology model of ADAMTS7 was developed based on the crystal structure of ADAMTS5 in complex with Batimastat (PDB code 2RJQ). Compound 3a was docked into the metalloproteinase domain of both ADAMTS5 and ADAMTS7 using the GOLD software, and each of the two predicted ligand–protein complexes was subjected to 451 ns of MD simulation studies (see Experimental Section for further information). The poses identified for ADAMTS5 and ADAMTS7 isoforms are highly stable, with an average RMSD below 1.0 Å (Fig. S1†). As shown in Fig. 3, the predicted binding mode for 3a was similar for both proteases although some differences were evident. The hydroxamic acid chelated the zinc ion and interacted with the surrounding residues through hydrogen bonds. In particular, interactions were formed with ADAMTS5 residues E411 and G380 and ADAMTS7 residues E389 and G358. Similarly, the sulfonamide moiety interacts by hydrogen bonding with the backbone of the residues L379 and G380 in ADAMTS5, the same interactions being observed in ADAMTS7 with L357 and G358. A first difference between the two binding modes was highlighted by the orientation of the benzoylpiperidine moiety; this fragment showed in ADAMTS7 a hydrophobic interaction with M350 that was not present in the 3a-ADAMTS5 complex. The biphenyl moiety of 3a was positioned almost identically in both proteases, showing hydrophobic interactions; in particular, the benzene ring bound to the sulfonamide established a π–π interaction with H410 for ADAMTS5 and H388 for ADAMTS7. The trifluoromethyl substituted ring was in a plane defined by L438 and L433 in ADAMTS5, corresponding to residues I414 and L419 in ADAMTS7. An important difference was the formation of water-mediated hydrogen bonds in ADAMTS7. The presence of water molecules in the cavity surrounding the trifluoromethyl group provided further interactions compared to the arrangement of 3a in ADAMTS5, where no water molecule was able to occupy such space during the MD simulation. In ADAMTS7, the H-bond acceptor nature of trifluoromethyl allowed the formation of a water bridge with the D422 side chain. Furthermore, such moiety of 3a was involved in a network of interactions mediated by water molecules, allowing the formation of hydrogen bonds with residues P412 and A423. A further interaction mediated by two water molecules allowed the interaction with the side chain of D508. The differences here highlighted provide a mechanistic explanation for the selectivity of 3a for ADAMTS7 over ADAMTS5.

## 3. Conclusions

Our findings show that, compared to our starting compound EDV33, the introduction of a rigid and linear biphenylsulfonyl moiety in P1′ led to a shift of selectivity from ADAMTS5 to ADAMTS7. The best compound, 3a, a *p*-trifluoromethyl biphenyl sulfonamide, displayed a 12-fold selectivity for ADAMTS7 (*K*_i_ = 9 nM) over ADAMTS5 (*K*_i_ = 110 nM) and an 8-fold increase in inhibition of ADAMTS7 with respect to EDV33 (*K*_i_ = 70 nM), with similar inhibitory potency when LTBP4 was used as a protein substrate instead of a FRET peptide. Selectivity over ADAMTS5 is important because this enzyme represents an anti-target in cardiovascular diseases.^21^

The docking of 3a into models of ADAMTS5 and ADAMTS7 active sites provided a mechanistic explanation as to how the selectivity is mediated. The predicted orientation of the benzoylpiperidine moiety in ADAMTS7 showed a hydrophobic interaction with M350 that was not present in the 3a-ADAMTS5 complex. The docking also predicted water molecules in the cavity surrounding the trifluoromethyl group that were not present in the 3a-ADAMTS5 complex. In ADAMTS7, the H-bond acceptor nature of trifluoromethyl group may allow the formation of a water bridge with the D422 side chain. Additional water molecules around this group allowed the formation of hydrogen bonds with residues P412 and A423 and D508. Future X-ray crystallography studies of the ADAMTS7 metalloprotease domain in complex with 3a will be carried out to confirm this binding mode.

In conclusion, we present the development of a selective inhibitor of ADAMTS7 which provides a good basis for further optimization studies, pharmacokinetics studies and *in vivo* studies using models of atherosclerosis.

## 4. Experimental section

### 4.1. Chemistry

All experiments described here require the use of standard personal protective equipment for laboratory work, *i.e.* lab coats and gloves. Goggles to protect the eyes are required where safety symbols indicate the chemicals are corrosive. All reactions were performed in solution, mostly under N_2_ atmosphere in dried glassware and were followed by thin-layer chromatography (TLC) on Merck aluminium silica gel sheets (60 F254) visualized under an UV lamp (254 nm; 365 nm). Non chromophoric substances were stained with a phosphomolybdic acid solution and visualized after heating. Furthermore, FeCl_3_ aqueous solution was used as a staining solution to identify hydroxamic acids. Evaporation was conducted under vacuum condition in rotating evaporator. Anhydrous sodium sulfate was used as drying agent after extraction treatments. Chromatographic separations were performed on silica gel by using an Isolera Prime automatic system (Biotage, Uppsala, Sweden) and SFÄR HC Duo silica cartridges (Biotage, Uppsala, Sweden), equipped with a UV detector with variable wavelength (*λ* = 200–400 nm) or by using prepacked ISOLUTE Flash Si II cartridges (Biotage). Microwave-assisted reactions were run in a Biotage Initiator+ microwave synthesizer. ^1^H, ^13^C, ^19^F NMR spectra were recorded on a Bruker Avance III HD 400 MHz spectrometer. Chemical shift (*δ*) are reported in ppm and *J* in Hz. The following abbreviations were used to explain the multiplicities: s = singlet, d = doublet, t = triplet, q = quartet, m = multiplet, dt = double triplet, td = triple duplet, dd = double doublet, ds = double septet, br s = broad singlet. Melting points were determined on a Leica Galen III Microscope (Leica/Cambridge Instruments) and are uncorrected. The ESI-MS spectra were recorded by direct injection at 5 (positive) and 7 (negative) μL min^−1^ flow rate in an Orbitrap high-resolution mass spectrometer (Thermo, San Jose, CA, USA), equipped with HESI source. Elemental analysis was used to determine the purity of target compounds, which is >95%. Analytical results are within ± 0.4% of the theoretical values. All commercially available chemicals were purchased from Sigma-Aldrich, Abcr and AstaTech.

#### General procedure to synthesize sulfonamide carboxylates 6, 11, 20 and 24

To the appropriate commercial amino acid (1 eq.) dissolved in H_2_O/dioxane (v/v 1 : 1, 1 mL mmol^−1^) triethylamine (2 eq.) was added at 0 °C. Then, the appropriate sulfonyl chloride (1 eq.) was added slowly. The reaction mixture was maintained at 0 °C/RT for 0.5–24 h. The mixture was diluted with a solution of HCl 1 N and extracted with DCM/EtOAc (3×). The organic phases were combined, dried over Na_2_SO_4_, filtered and evaporated under vacuum conditions.

##### 5-((*tert*-Butoxycarbonyl)amino)-2-((4-((2-chloro-4-fluorobenzyl)oxy)phenyl)sulfonamido)-3-methylpentanoic acid (6)

The title compound was synthesized from 2-amino-5-((*tert*-butoxycarbonyl)amino)-3-methylpentanoic acid as a diastereomeric mixture and sulfonyl chloride 5 following the general procedure. The obtained residue was purified by flash chromatography (DCM/MeOH from 50 : 1 to 20 : 1 v/v) using a ISOLUTE Flash Si II 20 g cartridge to give compound 6 as a white solid. Yield: 79%. ^1^H NMR (400 MHz DMSO-d_6_) *δ*: 12.57 (br s, 1H); 7.81 (d, *J* = 10.8 Hz, 1H); 7.76–7.68 (m, 2H); 7.67–7.66 (m, 1H); 7.54 (dd, *J*_1_ = 2.4 Hz, *J*_2_ = 8.8 Hz, 1H); 7.32–7.27 (m, 1H); 7.26–7.15 (m, 2H); 6.76 (t, *J* = 4.8 Hz, 1H); 5.19 (s, 2H); 3.68 (dd, *J*_1_ = 4.8 Hz, *J*_2_ = 6.8 Hz, 1H); 2.99–2.92 (m, 1H); 2.88–2.81 (m, 1H); 1.89–1.87 (m, 1H); 1.36 (s, 9H); 1.23–1.19 (m, 2H); 0.75 (d, *J* = 6.8 Hz, 3H).

##### (*R*)-3-(1-(*tert*-Butoxycarbonyl)piperidin-4-yl)-2-((4-((2-chloro-4-fluorobenzyl)oxy)phenyl) sulfonamido)propanoic acid (11)

The title compound was synthesized from (*R*)-2-amino-3-(1-(*tert*-butoxycarbonyl)piperidin-4-yl)propanoic acid and sulfonyl chloride 5 following the general procedure. White solid, yield: 85%. ^1^H NMR (400 MHz, CDCl_3_) *δ*: 7.81–7.78 (m, 2H); 7.49 (dd, *J*_1_ = 6 Hz, *J*_2_ = 8.8 Hz, 1H); 7.18 (dd, *J*_1_ = 2.4 Hz, *J*_2_ = 9.2 Hz, 1H); 7.05–7.00 (m, 3H); 5.27 (br d, *J* = 8.8 Hz, 1H); 5.15 (s, 2H); 4.02–3.99 (m, 2H); 3.93–3.90 (m, 1H); 2.63–2.50 (m, 2H); 1.65–1.53 (m, 5H); 1.44 (s, 9H); 1.20–1.05 (m, 1H).

##### (*R*)-2-(1-(*tert*-Butoxycarbonyl)piperidin-4-yl)-2-((4-((2-chloro-4-fluorobenzyl)oxy) phenyl)sulfonamido)acetic acid (20)

The title compound was synthesized from (*R*)-2-amino-2-(1-(*tert*-butoxycarbonyl)piperidin-4-yl)acetic acid following the general procedure. The residue was triturated with *n*-hexane to yield pure compound 20 as a white solid (159 mg). Yield: 73%. ^1^H NMR (400 MHz, DMSO-d_6_) *δ*: 12.73 (br s, 1H); 7.97 (d, *J* = 9.2 Hz, 1H); 7.72–7.51 (m, 4H); 7.31–7.16 (m, 3H); 5.21 (s, 2H); 3.90–3.88 (m, 2H); 3.58–3.56 (m, 1H); 2.70–2.60 (m, 2H); 1.76–1.75 (m, 1H); 1.50–1.36 (m, 2H); 1.37 (s, 9H); 1.23–1.21 (m, 2H).

##### (*R*)-2-((4-Bromophenyl)sulfonamido)-2-(1-(*tert*-butoxycarbonyl) piperidin-4-yl)acetic acid (24)

The title compound was synthesized from (*R*)-2-amino-2-(1-(*tert*-butoxycarbonyl)piperidin-4-yl)acetic acid following the general procedure. Trituration of the crude product with *n*-hexane afforded pure carboxylic acid 24, as a yellow semi-solid. Yield: 81%. ^1^H NMR (400 MHz CDCl_3_) *δ*: 7.80–7.79 (m, 1H); 7.70–7.61 (m, 3H); 5.41 (d, *J* = 9.6 Hz, 1H); 4.12–4.10 (m, 4H); 3.82–3.79 (m, 1H); 2.68–2.59 (m, 2H); 1.90–1.72 (m, 1H); 1.57–1.54 (m, 1H); 1.44 (s, 9H); 1.25–1.18 (m, 1H).

##### 1-(*tert*-Butyl) 4-methyl 4-((4-((2-chloro-4-fluorobenzyl)oxy)phenyl) sulfonamido)piperidine-1,4-dicarboxylate (12)

To a solution of commercial methyl 1-Boc-4-aminopiperidine-4-carboxylate (300 mg, 1.16 mmol, 1 eq) and pyridine (0.155 mL mmol^−1^, 0.18 mL) in dry CHCl_3_ (3.63 mL mmol^−1^, 4.2 mL), sulfonyl chloride 5 (425.6 mg, 1.27 mmol, 1.1 eq) was added portion wise. The reaction mixture was stirred at RT overnight under argon atmosphere and then the solvent was evaporated. The crude product was diluted with H_2_O and extracted with CHCl_3_ (3 × 125 mL). The organic solution was dried over Na_2_SO_4_, filtered and evaporated to obtain the pure product 12 as a colorless oil (598 mg). Yield: 84%. ^1^H NMR (400 MHz, CDCl_3_) *δ*: 7.82–7.79 (m, 2H); 7.61–7.58 (m, 1H); 7.49 (dd, *J*_1_ = 5.2 Hz, *J*_2_ = 8.4 Hz, 1H); 7.05–7.01 (m, 3H); 5.18 (s, 2H); 3.64–3.62 (m, 2H); 3.10–2.90 (m, 2H); 1.93–1.90 (m, 4H); 1.42 (s, 9H).

#### General procedure to synthesize methyl esters 7, 13, 25 and 26

To a solution of carboxylic acid 6, 11 or 24 (1 eq.) in dry DMF (3.125 mL mmol^−1^), K_2_CO_3_ (1.1 eq.) and CH_3_I (1.1 eq.) were added portion wise. The resulting mixture was stirred at RT for 3 h under nitrogen atmosphere. The resulting solution was diluted with H_2_O and extracted with DCM. The combined organic phases were dried over Na_2_SO_4_, filtered and evaporated.

##### Methyl 5-((*tert*-butoxycarbonyl)amino)-2-((4-((2-chloro-4-fluorobenzyl)oxy)phenyl)sulfonamido)-3-methylpentanoate (7)

The title compound was synthesized from carboxylic acid 6 following the general procedure. The residue was purified by flash chromatography using ISOLUTE Si II 10 g cartridge (*n*-hexane/EtOAc 75 : 25 v/v) to give compound 7. Yield: quantitative. ^1^H NMR (400 MHz CDCl_3_) *δ*: 7.79–7.77 (m, 2H); 7.49 (dd, *J*_1_ = 6 Hz; *J*_2_ = 8.8 Hz, 1H); 7.18 (dd, *J*_1_ = 2.4 Hz, *J*_2_ = 8.4 Hz, 1H); 7.05–7.01 (m, 3H); 5.17 (s, 2H); 5.09 (d, *J* = 10 Hz, 1H); 3.93 (dd, *J*_1_ = 3.2 Hz, *J*_2_ = 9.6 Hz, 1H); 3.47 (s, 3H); 3.27–3.25 (m, 1H); 3.14–3.07 (m, 1H); 1.71–1.64 (m, 2H); 1.44 (s, 9H); 0.81 (d, *J* = 6.8 Hz, 3H).

##### *Tert*-Butyl (*R*)-4-(2-((4-((2-chloro-4-fluorobenzyl)oxy)phenyl) sulfonamido)-3-methoxy-3-oxopropyl)piperidine-1-carboxylate (13)

The title compound was synthesized from carboxylic acid 11 following the general procedure. The residue was purified by flash chromatography (ISOLERA Biotage) using a Snap Ultra 25 g cartridge (in gradient from *n*-hexane/EtOAc from 4 : 1 to 1 : 1 v/v) to give pure 13 as a colorless oil. Yield: 34%. ^1^H NMR (400 MHz, CDCl_3_) *δ*: 7.79–7.77 (m, 2H); 7.49 (dd, *J*_1_ = 6 Hz, *J*_2_ = 8.8 Hz, 1H); 7.18 (dd, *J*_1_ = 2.4 Hz, *J*_2_ = 8.4 Hz, 1H); 7.05–7.01 (m, 3H); 5.17 (s, 2H); 5.02 (d, *J* = 10 Hz, 1H); 4.07–4.04 (m, 2H); 3.94–3.93 (m, 1H); 3.46 (s, 3H); 2.65–2.62 (m, 2H); 1.70–1.50 (m, 6H); 1.44 (s, 9H); 1.20–1.00 (m, 1H).

##### *Tert*-Butyl (*R*)-4-(1-((4-bromophenyl)sulfonamido)-2-methoxy-2-oxoethyl)piperidine-1-carboxylate (25)

The title compound was synthesized from carboxylic acid 24 following the general procedure. The residue was purified by flash chromatography using a ISOLUTE Si II 5 g cartridge (*n*-hexane/EtOAc gradient from 15 : 1 to 3 : 1 v/v) to give the desired product 25 as a colorless oil. Yield: 53%. ^1^H NMR (400 MHz CDCl_3_) *δ*: 7.69–7.63 (m, 4H); 5.12 (d, *J* = 10 Hz, 1H); 4.15–4.11 (m, 2H); 3.78–3.75 (m, 1H); 3.49 (s, 3H); 2.64–2.04 (m, 2H); 1.83–1.78 (m, 1H); 1.44 (s, 9H); 1.37–1.19 (m, 4H).

##### *Tert*-Butyl (*R*)-4-(1-((4-bromo-*N*-methylphenyl)sulfonamido)-2-methoxy-2-oxoethyl)piperidine-1-carboxylate (26)

The title compound was synthesized from carboxylic acid 24 following the general procedure. The residue was purified by flash chromatography using a ISOLUTE Si II 20 g cartridge (*n*-hexane/EtOAc gradient from 10 : 1 to 1 : 1 v/v) to give the desired product 26 as a white solid. Yield: 44%. ^1^H NMR (400 MHz CDCl_3_) *δ*: 7.66–7.62 (m, 4H); 4.25 (d, *J* = 10.8 Hz, 1H); 4.13–4.11 (m, 2H); 3.43 (s, 3H); 2.85 (s, 3H); 2.70–2.67 (m, 2H); 2.01–1.89 (m, 1H); 1.75–1.72 (m, 1H); 1.52–1.48 (m, 1H); 1.45 (s, 9H); 1.31–1.25 (m, 3H).

##### *Tert*-Butyl 4-((1*R*)-1-((4-((2-chloro-4-fluorobenzyl)oxy)phenyl)sulfonamido)-2-oxo-2-(((tetrahydro-2H-pyran-2-yl)oxy)amino)ethyl)piperidine-1-carboxylate (21)

To a solution of the carboxylic acid 20 (159 mg, 0.29 mmol, 1 eq.) in dry DMF (2.21 mL mmol^−1^, 0.63 mL), HOBt (46 mg, 0.34 mmol, 1.2 eq.), NMM (0.09 mL, 0.86 mmol, 3 eq.), THPONH_2_ (104 mg, 0.89 mmol, 3.1 eq.), and lastly EDC (77 mg, 0.40 mmol, 1.4 eq.) were added. After stirring at RT overnight, the mixture was treated with NaHCO_3_ and extracted with EtOAc. The combined organic extracts were dried over anhydrous Na_2_SO_4_, filtered, and evaporated under reduced pressure. The residue was purified by flash chromatography (ISOLERA Biotage) using a SNAP Ultra 10 g column (CHCl_3_) to yield pure compound 21 as a colorless oil (99.5 mg). Yield: 53%. ^1^H NMR *diastereomeric mixture* (400 MHz, CDCl_3_) *δ*: 7.77 (d, *J* = 8.8 Hz, 4H); 7.51–7.48 (m, 2H); 7.20–7.17 (m, 2H); 7.06–7.02 (m, 6H); 5.24–5.23 (m, 2H); 5.16 (d, *J* = 2.4 Hz, 4H); 4.83–4.82 (m, 2H); 4.48–4.47 (m, 2H); 4.11–4.10 (m, 4H); 3.84–3.82 (m, 2H); 3.59–3.56 (m, 2H); 3.42–3.37 (m,2H); 2.61–2.60 (m, 4H); 1.79–1.45 (m, 22H); 1.44 (s, 18H).

#### General procedure to synthesize trifluoroacetate salts 8, 14–15, 22, 27 and 28

Trifluoroacetic acid (30 eq.) was slowly added to a solution of compound 7, 12–14, 21, 25 or 26 (1 eq) in dry DCM (6.28 mL mmol^−1^) at 0 °C. The reaction mixture was stirred at 0 °C/RT for 15 min - 3 h. The solvent was co-evaporated in high vacuum conditions with the appropriate solvent.

##### 4-((4-((2-Chloro-4-fluorobenzyl)oxy)phenyl)sulfonamido)-5-methoxy-3-methyl-5-oxopentan-1-aminium trifluoroacetate (8)

The title compound was synthesized from methyl ester 7 following the general procedure. Purification by flash chromatography using a ISOLUTE Si II 10 g cartridge (CHCl_3_/MeOH gradient from 30 : 1 to 10 : 1 v/v) afforded pure compound 8. Yield: 91%. ^1^H NMR (400 MHz DMSO-d_6_) *δ*: 7.71–7.66 (m, 5H); 7.55 (dd, *J*_1_ = 2.4 Hz; *J*_2_ = 8.8 Hz, 1H); 7.32–7.27 (m, 1H); 7.20–7.18 (m, 2H); 5.22 (s, 2H); 3.80–3.78 (m, 1H); 3.28 (s, 3H); 2.82–2.73 (m, 2H); 1.99–1.96 (m, 1H); 1.63–1.60 (m, 1H); 1.45–1.40 (m, 1H); 0.78 (d, *J* = 6.8 Hz, 3H).

##### 4-((4-((2-Chloro-4-fluorobenzyl)oxy)phenyl)sulfonamido)-4-(methoxycarbonyl)piperidin-1-ium trifluoroacetate (14)

The title compound was synthesized from methyl ester 12 following the general procedure. Purification of the crude product by trituration with Et_2_O yielded pure compound 14 as a colorless oil. Yield: 92%. ^1^H NMR (400 MHz, DMSO-d_6_) *δ*: 8.52–8.48 (br s, 2H); 7.80–7.77 (m, 1H); 7.71–7.66 (m, 2H); 7.54 (dd, *J*_1_ = 6 Hz, *J*_2_ = 8.4 Hz, 1 H); 7.29 (td, *J*_1_ = 2.4 Hz, *J*_2_ = 8.4 Hz, 1H); 7.22 (d, *J* = 8.8 Hz, 2H); 5.23 (s, 2H); 3.30 (s, 3H); 3.22–3.20 (m, 1H); 3.10–3.07 (m, 2H); 2.94–2.92 (m, 2H); 2.10–2.08 (m, 2H); 1.99–1.97 (m, 2H).

##### (*R*)-4-(2-((4-((2-Chloro-4-fluorobenzyl)oxy)phenyl)sulfonamido)-3-methoxy-3-oxopropyl)piperidin-1-ium trifluoroacetate (15)

The title compound was synthesized from methyl ester 13 following the general procedure. The residue was purified by trituration with Et_2_O affording compound 15 as a pure white solid (126 mg). Yield: quantitative. ^1^H NMR (400 MHz, MeOD) *δ*: 7.78–7.76 (m, 2H); 7.60 (dd, J_1_= 6 Hz, J_2_= 8.8 Hz, 1H); 7.31 (dd, *J*_1_ = 2.8 Hz, *J*_2_ = 8.8 Hz, 1H); 7.16–7.13 (m, 3H); 5.23 (s, 2H); 3.94 (t, *J* = 7.6 Hz, 1H); 3.37–3.36 (m, 2H); 3.36 (s, 3H); 2.99–2.92 (m, 2H); 2.02–1.87 (m, 4H); 1.44–1.34 (m, 3H).

##### 4-((1*R*)-1-((4-((2-Chloro-4-fluorobenzyl)oxy)phenyl)sulfonamido)-2-oxo-2-(((tetrahydro-2*H*-pyran-2-yl)oxy)amino)ethyl)piperidin-1-ium trifluoroacetate (22)

The title compound was synthesized from methyl ester 21 following the general procedure. The residue was purified by flash chromatography (ISOLERA Biotage), using a SNAP Ultra 10 g cartridge (CHCl_3_/MeOH gradient from 90 : 10 to 70 : 30 v/v) yielded pure compound 22 as colorless oil. Yield: 32%. ^1^H NMR (400 MHz, MeOD) *δ*: 7.81–7.77 (m, 2H); 7.64–7.60 (m, 1H); 7.36–7.33 (m, 1H); 7.19–7.10 (m, 3H); 5.23 (d, *J* = 10.4 Hz, 2H); 4.45–4.44 (m, 1H); 3.92–3.88 (m, 2H); 3.59–3.43 (m, 3H); 2.98–2.93 (m, 2H); 2.21–2.10 (m, 2H); 1.92–1.31 (m, 10H).

##### (*R*)-4-(1-((4-Bromophenyl)sulfonamido)-2-methoxy-2-oxoethyl)piperidin-1-ium trifluoroacetate (27)

The title compound was synthesized from methyl ester 25 following the general procedure. The residue was purified by flash chromatography using a ISOLUTE Si II 10 g cartridge (CHCl_3_/MeOH gradient from 30 : 1 to 10 : 1 v/v) to give compound 27 as a hygroscopic white solid. Yield: 72%. ^1^H NMR (400 MHz, CDCl_3_) *δ*: 9.34 (br s, 1H); 8.80 (br s, 1H); 7.70–7.63 (m, 4H); 5.96 (d, *J* = 9.2 Hz, 1H); 3.82 (q, *J* = 5.6 Hz, 1H); 3.50 (s, 3H); 3.47–3.45 (m, 2H); 2.88–2.86 (m, 1H); 2.26–2.17 (m, 1H); 1.82–1.66 (m, 4H). ^13^C NMR (100 MHz, CDCl_3_): 171.4, 138.8, 132.4, 128.9, 128.0, 60.5, 52.6, 46.1, 46.0, 39.8, 36.6, 29.8, 29.5, 28.5.

##### (*R*)-4-(1-((4-Bromo-*N*-methylphenyl)sulfonamido)-2-methoxy-2-oxoethyl)piperidin-1-ium trifluoroacetate (28)

The title compound was synthesized from methyl ester 26 following the general procedure. Trituration with Et_2_O afforded pure 28 as a white solid. Yield: 95%. ^1^H NMR (400 MHz CDCl_3_) *δ*: 9.59–9.16 (m, 2H); 7.69–7.65 (m, 4H); 4.33 (d, *J* = 8.4 Hz, 1H); 3.60–3.55 (m, 2H); 3.42 (s, 3H); 2.99–2.98 (m, 2H); 2.80 (s, 3H); 2.12–2.04 (m,2H); 1.89–1.86 (m, 1H); 1.72–1.70 (m, 2H). ^19^F NMR (376 MHz, CDCl_3_) *δ*: −74.06.

#### General procedure to synthesize benzoylated derivatives 9, 16–17, 23, 29 and 30

Trifluoroacetate salt 8, 14–15, 22, 27 or 28 (1 eq.) was dissolved in dry DMF (9.5 mL mmol^−1^) under nitrogen atmosphere, then DIPEA (2 eq.) and benzoyl chloride (1 eq.) were added and the reaction mixture was stirred at RT for 1–3 h. The resulting mixture was diluted with H_2_O and extracted with EtOAc. The combined organic phases were dried over Na_2_SO_4_, filtered and evaporated.

##### Methyl 5-benzamido-2-((4-((2-chloro-4-fluorobenzyl)oxy)phenyl)sulfonamido)-3-methylpentanoate (9)

The title compound was synthesized from trifluoroacetate salt 8 following the general procedure. Yield: 99%. ^1^H NMR (400 MHz CDCl_3_) *δ*: 7.79–7.73 (m, 2H); 7.50–7.42 (m, 4H); 7.18 (dd, *J*_1_ = 2.4 Hz, *J*_2_ = 8 Hz, 1H); 7.02–7.00 (m, 3H); 6.31 (br s, 1H), 5.16 (br s, 1H); 5.14 (s, 2H); 4.00–3.98 (m, 1H); 3.68–3.64 (m, 1H); 3.48 (s, 3H); 3.44–3.41 (m, 1H); 2.16–2.06 (m, 1H); 1.87–1.83 (m, 1H); 1.67–1.61 (m, 1H); 0.85 (d, *J* = 6.8 Hz, 3H).

##### Methyl 1-benzoyl-4-((4-((2-chloro-4-fluorobenzyl)oxy)phenyl)sulfonamido)piperidine-4-carboxylate (16)

The title compound was synthesized from trifluoroacetate salt 14 following the general procedure. The residue was purified by flash chromatography (ISOLERA Biotage) using a Zip 10 g silica cartridge (CHCl_3_/MeOH 98 : 2 v/v) to give compound 16 as a colorless oil. Yield: 88%. ^1^H NMR (400 MHz, CDCl_3_) *δ*: 7.80–7.78 (m, 2H); 7.50–7.47 (m, 1H); 7.41–7.33 (m, 5H); 7.19 (dd, *J*_1_ = 2.4 Hz, *J*_2_ = 8.4 Hz, 1H); 7.06–7.02 (m, 3H); 5.17 (s, 2H); 5.02 (s, 1H); 4.18–4.16 (m, 1H); 3.52 (s, 3H); 3.52–3.50 (m, 1H); 3.29–3.28 (m, 2H); 2.00–1.97 (m, 2H); 1.73–1.72 (m, 2H).

##### Methyl (*R*)-3-(1-benzoylpiperidin-4-yl)-2-((4-((2-chloro-4-fluorobenzyl)oxy)phenyl)sulfonamido)propanoate (17)

The title compound was synthesized from trifluoroacetate salt 15 following the general procedure. Yellowish oil, yield: 95%. ^1^H NMR (400 MHz, CDCl_3_) *δ*: 7.78–7.76 (m, 2H); 7.50–7.48 (m, 1H); 7.40–7.38 (m, 5H); 7.20–7.17 (m, 1H); 7.05–7.00 (m, 3H); 5.16 (s, 2H); 5.06 (d, *J* = 10 Hz, 1H); 4.70–4.69 (m, 1H); 3.96–3.90 (m, 1H); 3.76–3-73 (m, 1H); 3.46 (s, 3H); 2.92–2.78 (m, 2H); 1.82–1.25 (m, 5H).

(*2R*)-2-(1-Benzoylpiperidin-4-yl)-2-((4-((2-chloro-4-fluorobenzyl)oxy)phenyl)sulfonamido)-*N*-((tetrahydro-2*H*-pyran-2-yl)oxy)acetamide (23)

The title compound was synthesized from trifluoroacetate salt 22 following the general procedure. The residue was purified by trituration with *n*-hexane to give compound 23 as yellowish oil. Yield: 50%. ^1^H NMR (400 MHz, MeOD) *δ*: 7.81–7.77 (m, 2H); 7.64–7.60 (m, 1H); 7.46–7.44 (m, 3H); 7.37–7.35 (m, 2H); 7.34–7.30 (m, 1H); 7.19–7.10 (m, 3H); 5.23 (d, *J* = 10.4 Hz, 2H); 4.45–4.44 (m, 1H); 3.92–3.88 (m, 2H); 3.59–3.43 (m, 3H); 2.98–2.93 (m, 2H); 2.21–2.10 (m, 2H); 1.92–1.31 (m, 10H).

##### Methyl (*R*)-2-(1-benzoylpiperidin-4-yl)-2-((4-bromophenyl)sulfonamido)acetate (29)

The title compound was synthesized from trifluoroacetate salt 27 following the general procedure. The residue was purified by flash chromatography using a ISOLUTE Si II 10 g cartridge (petroleum ether/EtOAc gradient from 3 : 1 to 1 : 1 v/v) to give compound 29 as a white solid. Yield: 75%. ^1^H NMR (400 MHz CDCl_3_) *δ*: 7.70–7.63 (m, 4H); 7.41–7.35 (m, 5H); 5.22 (d, *J* = 9.6 Hz, 1H); 4.77–4.75 (m, 1H); 3.83–3.80 (m, 2H); 3.50 (s, 3H); 2.78–2.70 (m, 1H); 2.00–1.96 (m, 2H); 1.67–1.41 (m, 4H).

##### Methyl (*R*)-2-(1-benzoylpiperidin-4-yl)-2-((4-bromo-*N*-methylphenyl)sulfonamido)acetate (30)

The title compound was synthesized from trifluoroacetate salt 28 following the general procedure. The residue was purified by flash chromatography using a ISOLUTE Si II 10 g cartridge (CHCl_3_) to give compound 30 as a dirty-white solid. Yield: 95%. ^1^H NMR (400 MHz CDCl_3_) *δ*: 7.65–7.63 (m, 4H); 7.42–7.39 (m, 5H); 5.22 (d, *J* = 9.6 Hz, 1H); 4.75–4.69 (m, 1H); 4.30 (d, *J* = 10.4 Hz, 1H); 3.85–3.69 (m, 1H); 3.43 (s, 3H); 2.97–2.94 (m, 1H); 2.84 (s, 3H); 2.12–2.04 (m, 1H); 1.81–1.79 (m, 1H; 1.55–1.23 (m, 4H).

#### General procedure to synthesize biphenyl derivatives 31a–g

To a solution of aryl bromide 29 or 30 (1 eq.) in a mixture of dioxane/H_2_O (4.4 : 1 v/v), K_3_PO_4_ (2.3 eq.), Pd(PPh_3_)_4_ (0.05 eq.) and the appropriate boronic acid (1.7 eq.) were added. The solution was heated under microwave irradiation to 70 °C for 15–30 min and then filtered through celite. With the exception of compound 31g, which was directly purified by chromatography, the crude products were diluted with a saturated solution of NaHCO_3_ and the aqueous phase was extracted with EtOAc (2×). The organic phase was dried over Na_2_SO_4_, filtered and evaporated under vacuum conditions. Purification of the residue by flash chromatography using a ISOLUTE Si II 5–10 g cartridge afforded the pure biphenyl derivatives 31a–g.

##### Methyl (*R*)-2-(1-benzoylpiperidin-4-yl)-2-((4′-(trifluoromethyl)-[1,1′-biphenyl])-4-sulfonamido)acetate (31a)

Biphenyl derivative 31a was synthesized from aryl bromide 29 and 4-(trifluoromethyl)phenylboronic acid following the general procedure. Purification by flash chromatography using a ISOLUTE Si II 5 g cartridge (petroleum ether/EtOAc from 3 : 1 to 2 : 1 v/v) afforded pure 31a as a colorless oil. Yield: 93%. ^1^H NMR (400 MHz CDCl_3_) *δ*: 7.95–7.90 (m, 2H); 7.76–7.95 (m, 6H); 7.45–7.37 (m, 5H); 5.20 (d, *J* = 9.6 Hz, 1H); 4.76–4.72 (m, 1H); 3.86–3.83 (m, 2H); 3.47 (s, 3H); 2.96–2.72 (m, 2H); 1.99–1.98 (m, 1H); 1.70–1.31 (m, 4H).

##### Methyl (*R*)-2-(1-benzoylpiperidin-4-yl)-2-((*N*-methyl-4′-(trifluoromethyl)-[1,1′-biphenyl])-4-sulfonamido)acetate (31b)

Biphenyl derivative 31b was synthesized from aryl bromide 30 and 4-(trifluoromethyl)phenylboronic acid following the general procedure. Purification by flash chromatography using a ISOLUTE Si II 5 g cartridge (petroleum ether/CHCl_3_ gradient from 4 : 1 to 3 : 1 v/v) gave biphenyl derivative 31b pure as a white solid. Yield: 83%. ^1^H NMR (400 MHz CDCl_3_) *δ*: 7.90–7.88 (m, 2H); 7.73–7.71 (m, 6H); 7.42–7.40 (m, 5H); 4.72–4.70 (m, 1H); 4.36 (d, *J* = 10.4 Hz, 1H); 3.86–3.84 (m, 1H); 3.42 (s, 3H); 3.00–2.95 (m, 1H); 2.89 (s, 3H); 2.16–2.01 (m, 1H); 2.01–2.00 (m,1H); 1.49–0.88 (m, 4H).

##### Methyl (*R*)-2-(1-benzoylpiperidin-4-yl)-2-((3′-(trifluoromethyl)-[1,1′-biphenyl])-4-sulfonamido)acetate (31c)

Biphenyl derivative 31c was synthesized from aryl bromide 29 and 3-(trifluoromethyl)phenylboronic acid following the general procedure. Purification by flash chromatography using a ISOLUTE Si II 5 g cartridge (petroleum ether/EtOAc gradient from 3 : 1 to 1 : 1 v/v) afforded 31c as a colorless oil. Yield: 89%. ^1^H NMR (400 MHz CDCl_3_) *δ*: 7.93–7.38 (m, 13H); 5.22 (d, *J* = 9.6 Hz, 1H); 4.77–4.75 (m, 1H); 3.70–3.90 (m, 2H); 3.48 (s, 3H); 3.08–2.57 (m, 2H); 1.99–1.97 (m, 1H); 1.70–1.31 (m, 4H).

##### Methyl (*R*)-2-(1-benzoylpiperidin-4-yl)-2-((4′-methoxy-[1,1′-biphenyl])-4-sulfonamido)acetate (31d)

Biphenyl derivative 31d was synthesized from aryl bromide 29 and 4-methoxyphenylboronic acid following the general procedure. Purification of the residue by flash chromatography using a ISOLUTE Si II, 5 g cartridge (petroleum ether/EtOAc gradient from 3 : 1 to 1 : 1 v/v) afforded pure 31d as a colorless oil. Yield: 98%. ^1^H NMR (400 MHz CDCl_3_) *δ*: 7.84–7.82 (m, 2H); 7.68–7.66 (m, 2H); 7.56–7.54 (m, 2H); 7.40–7.38 (m, 5H); 7.02–6.99 (m, 2H); 5.16 (d, *J* = 8.8 Hz, 1H); 4.76–4.74 (m, 1H); 3.85 (s, 3H); 3.69–3.67 (m, 2H); 3.44 (s, 3H); 2.95–2.93 (m, 1H); 2.71–2.69 (m, 1H); 1.97–1.95 (m, 1H); 1.68–1.16 (m, 4H).

##### Methyl (*R*)-2-(1-benzoylpiperidin-4-yl)-2-((4′-fluoro-[1,1′-biphenyl])-4-sulfonamido)acetate (31e)

Biphenyl derivative 31e was synthesized from aryl bromide 29 and (4-fluorophenyl)boronic acid following the general procedure. Purification by flash chromatography using a ISOLUTE Si II, 5 g cartridge (petroleum ether/EtOAc gradient from 3 : 1 to 2 : 1 v/v) gave pure 31e as a colorless oil (68 mg). Yield: 94%. ^1^H NMR (400 MHz CDCl_3_) *δ*: 7.88–7.86 (m, 2H); 7.69–7.64 (m, 2H); 7.58–7.53 (m, 2H); 7.41–7.34 (m, 5H); 7.19–7.15 (m, 2H); 5.18 (d, *J* = 10 Hz, 1H); 4.80–4.69 (m, 1H); 3.83–3.65 (m, 2H); 3.46 (s, 3H); 2.97–2.69 (m, 2H); 2.04–1.90 (m, 1H); 1.67–0.88 (m, 4H). ^19^F (376 MHz CDCl_3_) *δ*: −113.15.

##### Methyl (*R*)-2-(1-benzoylpiperidin-4-yl)-2-((4′-(dimethylamino)-[1,1′-biphenyl])-4-sulfonamido)acetate (31f)

Biphenyl derivative 31f was synthesized from aryl bromide 29 and 4-(dimethylamino)phenylboronic acid following the general procedure. In this specific case, important gas production has been observed while adding reagents to the microwave vial. Thus, a longer pre-stirring time was needed (15–20 min). Purification by flash chromatography using ISOLUTE Si II, 5 g cartridge (petroleum ether/EtOAc gradient from 3 : 1 v/v to EtOAc 100%) gave pure 31f as a brownish solid. Yield: 95%. ^1^H NMR (400 MHz CDCl_3_) *δ*: 7.81–7.79 (m, 2H); 7.67–7.65 (m, 2H); 7.54–7.52 (m, 2H); 7.40–7.38 (m, 5H); 6.80–6.78 (m, 2H); 5.12 (d, *J* = 10.4 Hz, 1H); 4.78–4.72 (m, 1H); 3.83–3.73 (m, 2H); 3.43 (s, 3H); 3.02 (s, 6H); 2.76–2.60 (m, 1H); 1.94–1.86 (m, 1H); 1.74–1.00 (m, 5H).

##### Methyl (*R*)-2-(1-benzoylpiperidin-4-yl)-2-((4′-morpholino-[1,1′-biphenyl])-4-sulfonamido)acetate (31g)

Biphenyl derivative 31g was synthesized from aryl bromide 29 and (4-morpholinophenyl)boronic acid following the general procedure. Purification by flash chromatography using ISOLUTE Si II, 10 g cartridge (isocratic eluent CHCl_3_ 100%) gave pure 31g as a white solid. Yield: 90%. ^1^H NMR (400 MHz CDCl_3_) *δ*: 7.83–7.81 (m, 2H); 7.68–7.64 (m, 2H); 7.56–7.51 (m, 2H); 7.40–7.37 (m, 5 h); 7.00–6.98 (m, 2H); 5.16 (d, *J* = 9.2 Hz, 1H); 4.76–4.68 (m, 1H); 3.89–3.86 (m, 4H); 3.85–3.84 (m, 2H); 3.43 (s. 3H); 3.25–3.22 (m, 4H); 2.87–2.65 (m, 2H); 2.00–1.89 (m, 1H); 1.33–0.81 (m, 4H).

#### General procedure to synthesize carboxylic acids 10, 18–19, 32a–g

To a solution of the proper derivative 9, 16–17, 31a–g (1 eq.) in THF or MeOH (22.7 mL mmol^−1^), LiOH 1 M (4.47 mL mmol^−1^) was added and the reaction mixture was stirred at 60 °C for 5–24 h. After quenching the reaction with HCl 1 N, the solution was extracted with EtOAc (3×). The organic phases were combined, dried over Na_2_SO_4_, filtered and evaporated.

##### 5-Benzamido-2-((4-((2-chloro-4-fluorobenzyl)oxy)phenyl)sulfonamido)-3-methylpentanoic acid (10)

Carboxylic acid 10 was obtained from derivative 9 following the general procedure. After extraction, trituration of the crude product with *n*-hexane afforded pure carboxylic acid 10. Yield: 75%. ^1^H NMR (400 MHz DMSO-d_6_) *δ*: 12.6 (br s, 1H); 8.42–8.40 (br s, 1H); 7.87–7.72 (m, 3H); 7.70–7.55 (m, 3H); 7.54–7.42 (m, 4H); 7.31–7.26 (m, 1H); 7.18–7.12 (m, 2H); 5.17 (s, 2H); 3.76–3.72 (m, 2H), 3.30–3.28 (m, 2H); 1.92–1.81 (m, 1H); 1.62–1.60 (m, 1H); 1.38–1.36 (m, 1H); 0.81 (d, *J* = 6.8 Hz, 3H).

##### 1-Benzoyl-4-((4-((2-chloro-4-fluorobenzyl)oxy)phenyl) sulfonamido)piperidine-4-carboxylic acid (18)

The title compound was synthesized from compound 16 following the general procedure. The crude product was purified by flash chromatography (ISOLERA Biotage) using a Sfar HC Duo 5 g cartridge (CHCl_3_/MeOH 98 : 2 v/v) to give 18 as a white solid. Yield: 42%. ^1^H NMR (400 MHz, DMSO-d_6_) *δ*: 12.00 (br s, 1H); 7.74–7.72 (m, 2H); 7.70–7.66 (m, 1H); 7.55 (dd, *J*_1_ = 2.4 Hz, *J*_2_ = 8.8 Hz, 1H); 7.41–7.39 (m, 3H); 7.33–7.26 (m, 3H); 7.19–7.17 (m, 2H); 5.20 (s, 2H); 3.90–3.88 (m, 1H); 3.26–3.24 (m, 1H); 3.01–2.93 (m, 2H); 1.83–1.81 (m, 4H).

##### (*R*)-3-(1-Benzoylpiperidin-4-yl)-2-((4-((2-chloro-4-fluorobenzyl)oxy)phenyl) sulfonamido)propanoic acid (19)

The title compound was synthesized from compound 17 following the general procedure. The crude product was purified by trituration with *n*-hexane/Et_2_O to give pure 19 as a white solid. Yield: quantitative. ^1^H NMR (400 MHz, MeOD) *δ*: 7.84–7-80 (m, 2H); 7.60–7.58 (m, 1H); 7.45–7.44 (m, 3H); 7.39–7.37 (m, 2H); 7.31–7.29 (m, 1H); 7.20–7.10 (m, 3H); 5.20–5.17 (m, 2H); 4.57–4.55 (m, 1H); 3.65–3.58 (m, 2H); 3.10–2.72 (m, 2H); 1.80–1.78 (m, 2H); 1.64–1.54 (m, 4H); 1.29–1.08 (m, 1H).

##### (*R*)-2-(1-Benzoylpiperidin-4-yl)-2-((4′-(trifluoromethyl)-[1,1′-biphenyl])-4-sulfonamido)acetic acid (32a)

The carboxylic acid 32a was obtained from biphenyl derivative 31a following the general procedure. After extraction, trituration of the crude product with Et_2_O afforded pure carboxylic acid 32a, as a white solid. Yield: 78%. ^1^H NMR (400 MHz CDCl_3_) *δ*: 7.92–7.90 (m, 2H); 7.72–7.65 (m, 6H); 7.44–7.31 (m, 5H); 5.55 (d, *J* = 35.2 Hz, 1H); 4.69–4.67 (m, 1H); 3.90–3.80 (m, 2H); 2.99–2.97 (m, 1H); 2.69–2.57 (m, 1H); 2.10–2.00 (m, 1H); 1.67–1.58 (m, 2H); 1.43–1.22 (m, 2H).

##### (*R*)-2-(1-Benzoylpiperidin-4-yl)-2-((*N*-methyl-4′-(trifluoromethyl)-[1,1′-biphenyl])-4-sulfonamido)acetic acid (32b)

The title compound was obtained from biphenyl derivative 31b in THF following the general procedure. After extraction, compound 32b was obtained as a white solid and used in the next reaction without further purification. Yield: 90%. ^1^H NMR (400 MHz CDCl_3_) *δ*: 7.91–7.89 (m, 2H); 7.70–7.67 (m, 6H); 7.38–7.35 (m, 5H); 4.65–4.64 (m, 1H); 4.40 (d, *J* = 10.8 Hz, 1H); 3.84–3.82 (m, 1H); 3.50–3.00 (m, 2H); 2.84 (s, 3H); 2.11–2.03 (m, 1H); 1.80–1.75 (m, 2H); 1.50–0.80 (m, 2H).

##### (*R*)-2-(1-Benzoylpiperidin-4-yl)-2-((3′-(trifluoromethyl)-[1,1′-biphenyl])-4-sulfonamido)acetic acid (32c)

The carboxylic acid 32c was obtained from biphenyl derivative 31c following the general procedure. After extraction, trituration of the crude product with *n*-hexane afforded pure carboxylic acid 32c as a white solid. Yield: 81%. ^1^H NMR (400 MHz CDCl_3_) *δ*: 7.91–7.89 (m, 2H); 7.80–7.79 (m, 1H); 7.74–7.72 (m, 1H); 7.67–7.65 (m, 3H); 7.60–7.51 (m, 2H); 7.45–7.31 (m, 4H); 5.58–5-53 (m, 1H); 4.69–4.67 (m, 1H); 3.88–3.63 (m, 2H); 2.98–2.93 (m, 1H); 2.68–2.65 (m, 1H); 2.04–2.00 (m, 1H); 1.57–1.33 (m, 4H). ^13^C NMR (100 MHz, MeOD) *δ*: 171.9; 171.0; 143.4; 140.4; 140.3; 135.7; 131.2; 130.9; 130.7; 129.6; 128.6; 128.3; 127.7; 127.3; 126.3; 125.5; 124.6; 123.6; 122.8; 65.5; 59.9; 44.7; 41.6; 38.7; 14.04.

##### (*R*)-2-(1-Benzoylpiperidin-4-yl)-2-((4′-methoxy-[1,1′-biphenyl])-4-sulfonamido)acetic acid (32d)

The carboxylic acid 32d was obtained from biphenyl derivative 31d following the general procedure. After extraction, the carboxylic acid 32d was obtained as a white solid without any further purification. Yield: quantitative. ^1^H NMR (400 MHz CDCl_3_) *δ*: 7.83–7.81 (m, 2H); 7.63–7.61 (m, 2H); 7.53–7.51 (m, 2H); 7.40–7.31 (m, 5H); 7.00–6.97 (m, 2H); 5.47–5.44 (m, 1H), 4.69–4.66 (m, 1H); 3.86 (s, 3H); 3.81–3.77 (m, 2H); 2.95–2.93 (m, 1H); 2.65–2.63 (m, 1H); 2.04–1.88 (m, 1H); 1.65–1.24 (m, 4H).

##### (*R*)-2-(1-Benzoylpiperidin-4-yl)-2-((4′-fluoro-[1,1′-biphenyl])-4-sulfonamido)acetic acid (32e)

The carboxylic acid 32e was obtained from biphenyl derivative 31e following the general procedure. After extraction, the residue was purified by flash chromatography using a ISOLUTE Si II 5 g cartridge (CHCl_3_/MeOH gradient from CHCl_3_ 100% to 30 : 1 v/v). The carboxylic acid 32e was obtained as a white solid. Yield: 97%. ^1^H NMR (400 MHz CDCl_3_) *δ*: 7.87–7.85 (m, 2H); 7.61–7.59 (m, 2H); 7.52–7.50 (m, 2H); 7.32–7.29 (m, 5H); 7.15–7.10 (m, 2H); 5.75 (br s, 1H); 4.66–4.63 (m, 1H); 3.81–3.62 (m, 2H); 2.90–2.62 (m, 2H); 2.10–2.03 (m, 1H); 1.50–1.00 (m, 4H).

##### (*R*)-2-(1-Benzoylpiperidin-4-yl)-2-((4′-(dimethylamino)-[1,1′-biphenyl])-4-sulfonamido)acetic acid (32f)

The title compound was obtained from biphenyl derivative 31f following the general procedure. After extraction, the residue was purified by flash chromatography using a ISOLUTE Si II 2 g cartridge (CHCl_3_/MeOH gradient from CHCl_3_ 100% to 10 : 1 v/v) and the carboxylic acid 32f was obtained as a white solid. Yield: 56%. ^1^H NMR (400 MHz CDCl_3_) *δ*: 7.81–7.79 (m, 2H); 7.64–7.61 (m, 2H); 7.52–7.50 (m, 2H); 7.39–7.34 (m, 5H); 6.81–6.79 (m, 2H); 5.42 (d, *J* = 8.8 Hz, 1H); 3.82–3.70 (m, 2H); 3.00 (s, 3H); 2.73–2.60 (m, 1H); 2.00–1.95 (m, 1H); 1.60–1.23 (m, 5H).

##### (*R*)-2-(1-Benzoylpiperidin-4-yl)-2-((4′-morpholino-[1,1′-biphenyl])-4-sulfonamido)acetic acid (32g)

The carboxylic acid 32g was obtained from biphenyl derivative 31g following the general procedure. After extraction the residue was purified by trituration with MeOH. The pure carboxylic acid 32g was afforded as a white solid. Yield: 54%. ^1^H NMR (400 MHz DMSO-d_6_) *δ*: 12.77 (br s, 1H); 8.08 (br s, 1H); 7.81–7.75 (m, 4H); 7.65–7.63 (m, 2H); 7.41–7.39 (m, 3H); 7.32–7.31 (m, 2H); 7.06–7.04 (m, 2H); 4.44–4.42 (m, 1H); 3.76–3.74 (m, 4H); 3.66–3.64 (m, 1H); 3.51–3.49 (m, 1H); 3.20–3.17 (m, 4H); 3.00–2.80 (m, 1H); 1.94–1.90 (m, 1H); 1.55–1.35 (m, 2H); 1.25–1.18 (m, 2H).

##### (*R*)-2-(1-Benzoylpiperidin-4-yl)-2-((4-((2-chloro-4-fluorobenzyl)oxy) phenyl)sulfonamido)-*N*-hydroxyacetamide (3)

To a solution of *O*-tetrahydropyranyl derivative 23 (17 mg, 0.03 mmol, 1 eq.) in dioxane (0.166 mL mmol^−1^, 0.5 mL), HCl 4 N (0.166 mL mmol^−1^, 0.5 mL) was added dropwise. Lastly, MeOH (66.7 mL mmol^−1^, 0.2 mL) was added and the reaction mixture was stirred at RT overnight. After evaporation, the residue was purified by flash chromatography using ISOLUTE Si II 2 g cartridge (CHCl_3_/MeOH gradient from CHCl_3_ 100% to 90% v/v) leading to pure final compound 3 as a white solid (5.7 mg). Yield: 33%. Mp: 38–41 °C. ^1^H NMR (400 MHz, MeOD) *δ*: 7.79 (d, *J* = 8.4 Hz, 2H); 7.59 (dd, *J*_1_ = 6.4 Hz, *J*_2_ = 8.8 Hz, 1H); 7.46–7.44 (m, 3H); 7.37–7.35 (m, 2H); 7.30 (dd, *J*_1_ = 2.4 Hz, *J*_2_ = 8.4 Hz, 1H); 7.15–7.10 (m, 3H); 5.22 (s, 2H); 4.61–4.58 (m, 1H); 3.02–2.98 (m, 1H); 2.77–2.75 (m, 1H); 1.92–1.85 (m, 1H); 1.84–1.40 (m, 2H); 1.17–1.11 (m, 2H). ^13^C NMR (100 MHz, MeOD) *δ*: 172.3; 168.8; 163.8 (d, *J*_1 C–F_ = 248 Hz); 163.2; 137.1; 135.3 (d, *J*_3 C–F_ = 10 Hz); 134.3; 132.4 (d, *J*_3 C–F_ = 9 Hz); 131.6 (d, *J*_4 C–F_ = 3 Hz); 131.0; 130.3; 129.7; 127.7; 117.8 (d, *J*_2 C–F_ = 25 Hz); 116.1; 115.3 (d, *J*_2 C–F_ = 21 Hz); 68.2; 59.7; 40.2; 30.7; 23.7. HRMS (ESI, *m*/*z*) calculated for C_27_H_27_N_3_O_6_FSCl [M − H]^−^: 574.12204; found: 574.12256. Elemental analysis for C_27_H_27_N_3_O_6_FSCl, calculated: % C, 56.30; % H, 4.72; % N, 7.29. Found: % C, 56.40; % H, 4.80; % N, 7.18.

#### General procedure to synthesize hydroxamates 1–2, 4 and 3a–g

To a solution of the carboxylic acid 10, 18–19 or 32a–g (1 eq.) in dry DMF (2.21 mL mmol^−1^), HOBt (1.2 eq.), NMM (3 eq.), THPONH_2_ (3 eq.), and lastly EDC (1.4 eq.) were added portionwise. The mixture was stirred at RT for 5–48 h under nitrogen atmosphere. This solution was diluted with a saturated solution of NaHCO_3_ and extracted with DCM (3×). The organic phases were combined, dried over Na_2_SO_4_, filtered and evaporated. The residue was purified by flash chromatography using ISOLUTE Si II to afford the *O*-tetrahydropyranyl derivative, which was used in the next step without further purification. The crude *O*-tetrahydropyranyl derivative was dissolved in MeOH/dioxane (1 : 1 v/v, 25.95 mL mmol^−1^) and HCl 4 N (10 mL mmol^−1^) was added. The reaction mixture was stirred at RT for 2–24 h and the solvent was evaporated.

##### *N*-(4-((4-((2-Chloro-4-fluorobenzyl)oxy)phenyl)sulfonamido)-5-(hydroxyamino)-3-methyl-5-oxopentyl)benzamide (1)

The title compound was synthesized from carboxylate 10 following the general procedure. The obtained crude was purified by flash chromatography using a ISOLUTE Si II 5 g cartridge (DCM/MeOH 100 : 1 v/v) leading to pure final compound 1 as a solid. Yield over two steps: 20%. Mp: 88–91 °C. ^1^H NMR *diastereomeric mixture* (400 MHz, DMSO-d6) *δ*: 10.44 (s, 1H); 8.80 (s, 1H); 8.42 (t, *J* = 4.8 Hz; 1H); 7.81 (d, *J* = 7.2 Hz, 2H); 7.74–7.64 (m, 4H); 7.55–7.48 (m, 2H); 7.46–7.42 (m, 2H); 7.30–7.28 (m, 1H); 7.13 (d, *J* = 3.2 Hz, 2H); 5.18 (s, 2H); 3.52–3.48 (m, 1H); 3.24–3.23 (m, 1H); 3.16–3.15 (m, 1H); 1.71–1.70 (m, 1H); 1.49–1.48 (m, 1H); 1.24–1.23 (m, 1H); 0.79 (d, *J* = 6.8 Hz, 3H). ^13^C NMR *diastereomeric mixture* (100 MHz, MeOD) *δ*: 170.4; 169.8; 163.8 (*δ*, *J*_1 C–F_ = 247 Hz); 163.1; 135.6; 135.3 (d, *J*_3 C–F_ = 10 Hz); 134.0; 132.6; 132.5; 132.3 (d, *J*_3 C–F_ = 9 Hz); 131.59 (d, *J*_4 C–F_ = 3 Hz); 130.4; 130.3; 129.5; 128.2; 117.8 (d, *J*_2 C–F_ = 25 Hz); 117.6; 116.0; 115.4; 115.3 (d, *J*_2 C–F_ = 19 Hz); 74.1; 69.1; 68.1; 62.1; 60.9; 59.0; 38.5; 35.6; 35.4; 34.0; 33.1; 16.2; 15.3. HRMS (ESI, *m*/*z*) calculated for C_26_H_27_N_3_O_6_FSCl [M − H]^−^: 562.12204; found: 562.12140. Elemental analysis for C_26_H_27_N_3_O_6_FSCl, calculated: % C, 55.37; % H, 4.83; % N, 7.45. Found: % C, 55.42; % H, 4.90; % N, 7.22.

##### 1-Benzoyl-4-((4-((2-chloro-4-fluorobenzyl)oxy)phenyl)sulfonamido)-*N*-hydroxypiperidine-4-carboxamide (2)

The title compound was synthesized from carboxylate 18 following the general procedure. The obtained crude was purified by flash chromatography using ISOLUTE Si II 2 g cartridge (DCM/MeOH gradient from DCM 100% to 10 : 1 v/v) leading to pure final compound 2 as a white solid. Yield over two steps: 20%. Mp: 89–93 °C. ^1^H NMR (400 MHz, MeOD) *δ*: 7.86–7.84 (d, *J* = 8.8 Hz, 2H); 7.62–7.60 (m, 1H); 7.47–7.45 (m, 3H); 7.37–7.35 (m, 2H); 7.32 (dd, *J*_1_ = 2.4 Hz, J_2_= 8.8 Hz, 1H); 7.18–7.14 (m, 3H); 5.26 (s, 2H); 4.13–4.10 (m, 1H); 3.46–3.43 (m, 1H); 3.21–3.15 (m, 2H); 2.15–1.96 (m, 4H). ^13^C NMR (100 MHz, MeOD) *δ*: 170.9; 170.5; 162.4 (d, *J*_1 C–F_ = 247.3 Hz); 161.8; 135.3; 134.4; 134.00 (d, *J*_3 C–F_ = 10 Hz); 131.0 (d, *J*_3 C–F_ =9 Hz); 130.2 (d, *J*_4 C–F_ = 3 Hz); 129.8; 129.0; 128.4; 126.4; 116.5 (d, *J*_2 C–F_ = 26 Hz); 114.7; 113.9 (d, *J*_2 C–F_ = 21 Hz); 66.8; 59.0; 43.2; 37.5; 32.8; 32.1. HRMS (ESI, *m*/*z*) calculated for C_26_H_25_N_3_O_6_FSCl [M − H]^−^: 560.10639; found: 560.10651. Elemental analysis for C_26_H_25_N_3_O_6_FSCl, calculated: % C, 55.57; % H, 4.48; % N, 7.48. Found: % C, 55.48; % H, 4.55; % N, 7.50.

##### (*R*)-3-(1-Benzoylpiperidin-4-yl)-2-((4-((2-chloro-4-fluorobenzyl)oxy)phenyl) sulfonamido)-*N*-hydroxypropanamide (4)

The title compound was synthesized from carboxylate 19 following the general procedure. The obtained crude was purified by flash chromatography using a ISOLUTE Si II 5 g cartridge (isocratic eluent CHCl_3_/MeOH 40 : 1 v/v) yielding pure final compound 4 as a white solid. Yield over two steps: 14%. Mp: 90–94 °C. ^1^H NMR (400 MHz MeOD) *δ*: 7.83–7.81 (m, 2H); 7.61–7.57 (m, 1H); 7.44–7.43 (m, 3H); 7.38–7.29 (m, 3H); 5.20 (d, *J* = 24.8 Hz, 2H); 4.57–4.46 (m, 1H); 2.94–2.47 (m, 4H); 1.73–1.72 (m, 1H); 1.51–1.08 (m, 7H). ^13^C NMR (100 MHz, MeOD) *δ*: 170.9; 162.5 (d, *J*_1 C–F_ = 248 Hz); 161.9; 135.8; 132.7; 130.1 (d, *J*_4 C–F_ = 4 Hz); 129.5; 129.0; 128.3; 126.3; 116.5 (d, *J*_2 C–F_ = 25 Hz); 114.8; 114.0 (d, *J*_2 C–F_ = 22 Hz); 66.9; 60.8; 39.1; 31.9; 29.3. HRMS (ESI, *m*/*z*) calculated for C_28_H_29_N_3_O_6_FSCl [M − H]^−^: 588.13769; found: 588.13800. Elemental analysis for C_28_H_29_N_3_O_6_FSCl, calculated: % C, 57.00; % H, 4.95; % N, 7.12. Found: % C, 56.93; % H, 4.99; % N, 7.15.

##### (*R*)-2-(1-Benzoylpiperidin-4-yl)-*N*-hydroxy-2-((4′-(trifluoromethyl)-[1,1′-biphenyl])-4 sulfonamido)acetamide (3a)

The title compound was synthesized from carboxylate 32a following the general procedure. The obtained crude was purified flash chromatography using a ISOLUTE Si II 5 g cartridge (CHCl_3_) yielding final compound 3a as a white solid. Yield over two steps: 46%. Mp: 170–173 °C. ^1^H NMR (400 MHz DMSO-d_6_) *δ*: 10.58 (s, 1H); 8.87 (s, 1H); 8.21–7.86 (m, 8H); 7.40–7.29 (m, 5H); 4.38–4.36 (m, 1H); 3.50–3.45 (m, 2H); 2.91–2.90 (m, 2H); 1.79–1.77 (m, 1H); 1.47–0.85 (m, 4H). ^13^C NMR (100 MHz, DMSO-d_6_) *δ*: 168.8; 165.9; 142.6; 142.0;141.2; 136.2; 129.3; 128.8; 128.5; 128.4; 127.9; 127.6; 127.1; 126.5; 126.0; 125.6; 122.9; 58.0; 46.7; 38.2; 28.6. HRMS (ESI, *m*/*z*) calculated for C_27_H_26_F_3_N_3_O_5_S [M − H]^−^: 560.14725; found: 560.14771; calculated for C_27_H_26_F_3_N_3_O_5_S [M + H]^+^: 562.16180, found: 562.16199. Elemental analysis for C_27_H_26_F_3_N_3_O_5_S, calculated: % C, 57.75; % H, 4.67; % N, 7.48. Found: % C, 57.72; % H, 4.60; % N, 7.53.

##### (*R*)-2-(1-Benzoylpiperidin-4-yl)-*N*-hydroxy-2-((*N*-methyl-4′-(trifluoromethyl)-[1,1′-biphenyl])-4-sulfonamido)acetamide (3b)

The title compound was synthesized from carboxylate 32b following the general procedure. The obtained crude was purified by flash chromatography using a ISOLUTE Si II 5 g cartridge (DCM/MeOH gradient from DCM 100% to 50 : 1 v/v) to give final compound 3b, as a white solid. Yield over two steps: 60%. Mp: 137–140 °C. ^1^H NMR (400 MHz, CDCl_3_) *δ*: 10.01 (br s, 1H); 7.87–7.82 (m, 2H); 7.71–7.62 (m, 6H); 7.39–7.37 (m, 5H); 4.69–4.67 (m, 1H); 4.10–4.08 (m, 1H); 3.81–3.80 (m, 1H); 3.04–3.00 (m, 1H); 2.95 (s, 3H); 2.90–2.75 (m, 1H); 2.28–2.26 (m, 1H); 1.30–0.88 (m, 4H). ^13^C NMR (100 MHz, CDCl_3_) *δ*: 170.8; 170.6; 165.0; 144.2; 142.6; 138.2; 135.1; 130.8; 130.4; 130.2; 128.7; 127.9; 127.7; 127.0; 126.0; 125.4; 122.7; 120.0; 60.3; 47.7; 42.2; 34.3; 30.3; 29.7; 29.5. HRMS (ESI, *m*/*z*) calculated for C_28_H_27_F_3_N_3_O_5_S [M – H]^−^: 574.16290; found: 574.16284. Elemental analysis for C_28_H_28_F_3_N_3_O_5_S, calculated: % C, 58.43; % H, 4.90; % N, 7.30. Found: % C, 58.22; % H, 4.96; % N, 7.33.

##### (*R*)-2-(1-Benzoylpiperidin-4-yl)-*N*-hydroxy-2-((3′-(trifluoromethyl)-[1,1′-biphenyl])-4-sulfonamido)acetamide (3c)

The title compound was synthesized from carboxylate 32c following the general procedure. Purification by flash chromatography using a ISOLUTE Si II 5 g cartridge (gradient: CHCl_3_/MeOH gradient from CHCl_3_ 100% to 50 : 1 v/v) gave pure final compound 3c, as a white solid. Yield over two steps: 45%. Mp: 126–129 °C. ^1^H NMR (400 MHz, MeOD) *δ*: 7.96–7.90 (m, 4H); 7.84–7.82 (m, 2H); 7.75–7.67 (m, 2H); 7.63–7.43 (m, 5H); 4.62–4.60 (m, 1H); 3.70–3.67 (m, 1H); 3.50–3.45 (m, 1H); 3.07–3.01 (m, 1H); 2.80–2.77 (m, 1H); 1.81–1.79 (m, 1H); 1.67–1.64 (m, 1H); 1.50–1.45 (m, 1H); 1.11–1.00 (m, 1H). ^13^C NMR (100 MHz, MeOD) *δ*: 172.4; 168.6; 145.0; 141.8; 137.0; 132.6; 132.3; 132.2; 131.0; 129.7; 129.3; 128.9; 127.7; 127.0; 126.0; 125.0; 124.2; 59.8; 54.8; 42.9; 40.1; 30.1; 29.5. HRMS (ESI, *m*/*z*) calculated for C_27_H_26_F_3_N_3_O_5_S [M − H]^−^: 560.14725; found: 560.14722. Elemental analysis for C_27_H_26_F_3_N_3_O_5_S, calculated: % C, 57.75; % H, 4.67; % N, 7.48. Found: % C, 57.68; % H, 4.73; % N, 7.57.

##### (*R*)-2-(1-Benzoylpiperidin-4-yl)-*N*-hydroxy-2-((4′-methoxy-[1,1′-biphenyl])-4-sulfonamido)acetamide (3d)

The title compound was synthesized from carboxylate 32d following the general procedure. Purification by flash chromatography using a ISOLUTE Si II 2 g cartridge (CHCl_3_/MeOH gradient from 100% CHCl_3_ to 30 : 1 v/v) gave pure final compound 3d, as a white solid. Yield over two steps: 44%. Mp: 193–197 °C. ^1^H NMR (400 MHz, DMSO-d_6_) *δ*: 10.58 (s, 1H); 8.87 (s, 1H); 8.01–7.99 (m, 1H); 7.80–7.78 (m, 3H); 7.71–7.69 (m, 2H); 7.41–7.29 (m, 5H); 7.08–7.06 (m, 2H); 4.37–4.35 (m, 1H); 3.64 (s, 3H); 3.46–3.44 (m, 2H); 2.89–2.67 (m, 2H); 1.77–1.75 (m, 2H); 1.55–1.14 (m, 4H). ^13^C NMR (100 MHz, DMSO-d_6_) *δ*: 168.3; 165.4; 159.2; 142.7; 138.9; 135.7; 130.2; 128.8; 127.9; 127.7; 126.6; 126.5; 126.0; 125.8; 114.0; 57.4; 54.8; 46.2; 37.7, 29.0. HRMS (ESI, *m*/*z*) calculated for C_27_H_29_N_3_O_6_S [M − H]^−^: 522.17043; found: 522.17053. Elemental analysis for C_27_H_29_N_3_O_6_S, calculated: % C, 61.94; % H, 5.58; % N, 8.03. Found: % C, 62.03; % H, 5.60; % N, 7.58.

##### (*R*)-2-(1-Benzoylpiperidin-4-yl)-2-((4′-fluoro-[1,1′-biphenyl])-4-sulfonamido)-*N*-hydroxyacetamide (3e)

The title compound was synthesized from carboxylate 32e following the general procedure. Purification of the obtained crude by flash chromatography using a ISOLUTE Si II 2 g cartridge (CHCl_3_/MeOH gradient from 100% CHCl_3_ to 50 : 1 v/v) afforded pure final compound 3e, as a white solid. Yield over two steps: 46%. Mp: 179–183. ^1^H NMR (400 MHz, DMSO-d_6_) *δ*: 10.56 (s, 1H); 8.84 (s, 1H); 8.09–8.06 (m, 1H); 7.82–7.76 (m, 6H); 7.40–7.27 (m, 7H); 4.40–4.33 (m, 1H); 3.48–3.39 (m, 2H); 2.91–2.59 (m, 2H); 1.79–1.77 (m, 2H); 1.56–0.85 (M, 4H). ^19^F (376 MHz, DMSO-d_6_) *δ*: −114.0. ^13^C NMR (100 MHz, MeOD-d_4_) *δ*: 172.4; 168.7; 164.5 (d, *J*_1 C–F_ = 245 Hz); 145.6; 140.9; 137.0 (d, *J*_4 C–F_ = 5 Hz); 131.0; 130.3 (d, *J*_3 C–F_ = 8 Hz); 129.7; 128.6 (d, *J*_2 C–F_ = 17 Hz); 127.7; 116.8 (d, *J*_2 C–F_ = 22 Hz); 59.8; 43.2; 42.9; 40.2; 30.7; 30.1; 29.2. HRMS (ESI, *m*/*z*) calculated for C_26_H_26_FN_3_O_5_S [M − H]^−^: 510.15044; found: 510.15012. Elemental analysis for C_26_H_26_FN_3_O_5_S, calculated: % C, 61.04; % H, 5.12; % N, 8.21. Found: % C, 61.20; % H, 5.03; % N, 8.32.

##### (*R*)-4′-(*N*-(1-(1-benzoylpiperidin-4-yl)-2-(hydroxyamino)-2-oxoethyl)sulfamoyl)-*N*,*N*-dimethyl-[1,1′-biphenyl]-4-aminium chloride (3f)

The title compound was synthesized from carboxylate 32f following the general procedure. Purification by flash chromatography using a ISOLUTE Si II 2 g cartridge (CHCl_3_/MeOH gradient from 30 : 1 to 10 : 1 v/v) gave pure compound 3f, as a white solid. The final compound was obtained as hydrochloride salt. Yield over two steps: 53%. Mp: 196–200 °C. ^1^H NMR (400 MHz, DMSO-d_6_) *δ*: 10.56 (s, 1H); 8.86–8.81 (m, 1H); 7.97–7.95 (m, 1H); 7.75–7.72 (m, 4H); 7.62–7.59 (m, 2H); 7.42–7.30 (m, 3H); 7.29–7.26 (m, 2H); 6.84–6.65 (m, 2H); 4.40–4.36 (m, 1H); 3.47–3.45 (m, 2H); 2.97 (s, 6H); 2.73–2.66 (M, 2H); 1.78–1.76 (m, 1H); 1.60–1.00 (m, 4H). ^13^C NMR (100 MHz, DMSO-d_6_) *δ*: 206.5; 168.8; 166.1; 150.5; 143.8; 138.3; 136.2; 129.3; 128.4; 127.5; 127.0; 126.5; 125.4; 125.3; 125.1; 125.0; 112.5; 111.9; 57.9; 38.3; 34.2; 29.5. HRMS (ESI, *m*/*z*) calculated for C_28_H_32_N_4_O_5_SxHCl [M − H]^−^: 571.17874; found: 571.17871. Elemental analysis for C_28_H_33_ClN_4_O_5_S, calculated: % C, 58.68; % H, 5.80; % N, 9.78. Found: % C, 58.73; % H, 5.98; % N, 9.82.

##### (*R*)-4-(4′-(*N*-(1-(1-Benzoylpiperidin-4-yl)-2-(hydroxyamino)-2-oxoethyl)sulfamoyl)-[1,1′-biphenyl]-4-yl)morpholin-4-ium chloride (3g)

The title compound was synthesized from carboxylate 32g following the general procedure. Purification by flash chromatography using ISOLUTE Si II 2 g cartridge (CHCl_3_/MeOH 20 : 1 v/v) gave pure final compound 3g, as a brownish highly hygroscopic solid. 3g was obtained as hydrochloride salt. Yield over two steps: 73%. Mp: 180–184 °C. ^1^H NMR (400 MHz, DMSO-d_6_) *δ*: 10.57 (s, 1H); 10.04; 8.01 (d, *J* = 8.8 Hz, 1H); 7.80–7.75 (m, 4H); 7.65–7.63 (m, 2H); 7.42.7.39 (m, 3H); 7.28–7.26 (m, 2H); 7.08–7.06 (m, 2H); 4.36–4.34 (m, 1H); 3.77–3.75 (m, 4H); 3.38–3.34 (m, 1H); 3.23–3.19 (m, 4H); 2.89–2.86 (m, 1H); 2.72–2.66 (m, 1H); 1.78–1.73 (m, 1H); 1.60–1.409 (m, 1H); 1.39–0.88 (m, 4H). ^13^C NMR (100 MHz, DMSO-d_6_) *δ*: 168.8; 166.0; 151.0; 143.4; 138.9; 136.2; 129.3; 128.6; 128.4; 127.6; 127.0; 126.5; 125.8; 115.1; 66.0; 57.9; 48.6; 47.9; 38.3. HRMS (ESI, *m*/*z*) calculated for C_30_H_34_N_4_O_6_S·HCl [M − H]^−^: 613.18931; found: 613.18994. Elemental analysis for C_30_H_35_ClN_4_O_6_S, calculated: % C, 58.58; % H, 5.74; % N, 9.11. Found: % C, 58.64; % H, 5.97; % N, 9.06.

### 4.2. Biological assays

#### 4.2.1. Enzyme inhibition assays

Pro-MMP1 and recombinant human ADAM17 were purchased from Calbiochem (Merck Millipore). Pro-MMP1 was activated immediately prior to use with *p*-aminophenylmercuric acetate (APMA, 2 mM for 2 h at 37 °C). The inhibitor stock solutions (DMSO, 10 mM) were further diluted in the fluorometric assay buffer (FAB, 50 mM Tris,pH 7.5, 150 mM NaCl, 10 mM CaCl_2_, 0.05% Brij-35 and 1% DMSO) following the protocol already reported.^44^ Activated MMP1 (2 nM) and inhibitor solutions were incubated in FAB for 3 h at 25 °C. ADAM17 (5 nM) was incubated for 30 min at 37 °C in Tris 25 mM, ZnCl_2_ 25 μM, Brij-35 0.005%, pH 9.0. After the addition of 200 μM solution of the fluorogenic substrate Mca-Lys-Pro-Leu-Gly-Leu-Dap(Dnp)-Ala-Arg-NH_2_ (Bachem) in DMSO (final concentration 2 μM), the hydrolysis was monitored every 15 s for 20 min recording the increase in fluorescence (*λ*_ex_ = 325 nm, *λ*_em_ = 400 nm) with a molecular devices SpectraMax Gemini XPS plate reader. The assays were performed in triplicate in a total volume of 200 μL per well in 96-well microtiter plates (Corning black, NBS). Control wells lacked inhibitor.

Human recombinant full-length ADAMTS1, ADAMTS4, ADAMTS5, and ADAMTS7-T8 (truncated before the C-terminal PLAC domain) were expressed, purified and quantified as before.^43,45–47^ Final concentrations of all ADAMTS proteases in FAB buffer was 10 nM. Assays were run in 384 well black plates (Cat. No: 784900, Greiner Bio-One, Austria) on a Spectramax I3x microplate reader using excitation and emission wavelengths of 485 and 520 nm, respectively. Following pre-incubation of enzyme and inhibitors, the following FRET substrates (all custom-synthesized by Bachem) were added at 40 μM: 5,6 fluorescein [FAM]-Ala-Glu-Leu-Gln-Gly-Arg-Pro-Ile-Ser-Ile-Ala-Lys-carboxytetramethylrhodamine [TAMRA] (ADAMTS1 and ADAMTS4, 2 h, 37 °C), FAM-Thr-Glu-Ser-Glu-Ser-Arg-Gly-Ala-Ile-Tyr-Lys-Lys-TAMRA (ADAMTS5) and FAM-Glu-Ala-Ala-Ala-Arg-Ala-Glu-Ala-Ala-Ala-Lys-TAMRA (ADAMTS7, 24 h, 37 °C).

Inhibitory activity was expressed in relative fluorescent units (RFU). Percent of inhibition was calculated from control reactions without the inhibitor. IC_50_ was determined using the formula: *v*_i_/*v*_o_ = 1/(1 + [I]/ IC_50_), where *v*_i_ is the initial velocity of substrate cleavage in the presence of the inhibitor at concentration [I] and *v*_o_ is the initial velocity in the absence of the inhibitor.

*K*_i_ values were determined using the Cheng–Prusoff equation:^48^*K*_i_ = IC_50_/(1 + [S]/*K*_m_)where [S] is the substrate concentration and *K*_m_ is the Michaelis Menten constant (MMP1: 28 μM, ADAMTS1: 13.5 μM; ADAMTS4: 23 μM; ADAMTS5: 76 μM; ADAMTS7:10.5 μM).

Results were analyzed using SoftMax Pro software and Origin 6.0 software.

LTBP4S-A cleavage assay was performed as described.^43^ Purified FLAG-tagged LTBP4S-A (3.5 μg) was incubated with ADAMTS7 (20 nM) for 24 h at 37 °C with and without pre-incubation with compound 3a (15.1–1000 nM). Following SDS-PAGE, cleavage fragments were immunoblotted using a mouse anti-FLAG (OctA probe) Ab sc-66355 (Santa Cruz Biotechnology) and a rabbit anti-neo epitope antibody. The latter was custom made by Life Technologies Ltd. and raised in rabbits against the sequence AAAPYTVKKK-C of LTBP4 conjugated to keyhole limpet hemocyanin. Antigen specific IgG was affinity purified using the same peptide followed by negative selection using the spanning peptide EAAAPYTVKKK-C. This purified neo-epitope antibody was detected by IRDye® 800CW goat anti-rabbit IgG (H + L) (Licor) and the anti-FLAG antibody by RDye® 680RD goat anti-mouse IgG (Licor). The protein ladder used was chameleon duo pre-stained protein ladder (Li-cor).

### 4.3. Computational methods

For docking and molecular dynamics calculations the X-ray structure of human ADAMTS5 bound to Batimastat (PDB code 2RJQ) was used.^49^ The human ADAMTS7 model was generated using the crystal structure of ADAMTS5 as a template. The average structure obtained from the molecular dynamics simulation of our previous work was used as the ADAMTS7 reference conformation. (ref. 30) Compound 3a was docked into the structure of ADAMTS5 and ADAMTS7 by means of GOLD software using ChemScore as the scoring function. The docking procedure generated a total of 50 poses for both calculations. To further investigate the two potential binding modes of 3a, the two best poses were subjected to 451 ns of MD simulation. All simulations were performed using AMBER version 20 (ref. 50) and carried out using the ff14SB force field at 300 K. General Amber force field (GAFF) parameters were assigned to the ligands, whereas partial charges were calculated using the AM1-BCC method with the Antechamber suite of AMBER 20. The two ligand–protein complexes were placed in a rectangular parallelepiped water-box, by using a TIP3P explicit solvent model and solvated with a 15.0 Å water cap. Sodium ions were added as counterions for the neutralization of the systems. Before MD simulations, two stages of energy minimization were carried out. In the first step, a position restraint of 100 kcal mol^−1^ Å^−2^ was applied to the complex, thus minimizing only the position of the water molecules through 5000 steps of steepest descent, followed by conjugate gradient until a convergence of 0.05 kcal mol^−1^ Å^−2^ was achieved. In a second step, the whole system was energy-minimized imposing a harmonic force constant of 10 kcal mol^−1^ Å^−2^ only on the protein α carbons. The minimized complexes were used as starting conformations for the MD simulations. An initial MD step of 1 ns with constant-volume periodic boundary conditions was performed and the temperature of the system was raised from 0 to 300 K. The system was then equilibrated through 50 ns of constant pressure periodic boundary MD, employing the Langevin thermostat in order to keep the temperature of the system constant. Then, an additional 400 ns of constant pressure MD production was performed. All the α carbons of the protein were restrained with a harmonic force constant of 10 kcal mol^−1^ Å^−2^ during the whole MD simulation.

## Data availability

The data supporting this article has been included as part of the ESI.†

## Author contributions

D. C.: investigation, methodology, validation, writing – original draft preparation. T. B., B. L. B., R. D. L., X. Z. and S. G.: investigation, methodology, validation. T. T.: methodology, formal analysis. A. R., M. M.: funding acquisition, project administration, supervision, writing – review & editing. S. S., R. dG. and E. N.: conceptualization, methodology, validation, supervision, funding acquisition, writing – original draft preparation. All authors have read and approved the published version of the manuscript.

## Conflicts of interest

No potential conflict of interest was reported by the authors.

## Supplementary Material

MD-015-D4MD00149D-s001
