# Supplementary material for: Design, synthesis and biological evaluation of arylsulfonamides as ADAMTS7 inhibitors
Source: RSC Med Chem. 2024 Jun 19;15(8):2806–25. doi: 10.1039/d4md00149d (PMC11324053; doi:10.1039/d4md00149d)
Supplement: MD-015-D4MD00149D-s001 [file MD-015-D4MD00149D-s001.pdf]

Electronic Supplementary Material (ESI) for RSC Medicinal Chemistry.
This journal is © The Royal Society of Chemistry 2024

SUPPORTING INFORMATION FOR

Design, synthesis and biological evaluation of arylsulfonamides as ADAMTS7 inhibitors

Doretta Cuffaro,[1,¶] *Tina Burkhard,*[2,¶] *Bianca Laura Bernardoni,*[1] *Riccardo Di Leo,*[1] *Xiaohan Zhang,*[3] *Salvatore Galati,*[1] *Tiziano Tuccinardi,*[1] *Marco Macchia,*[1] *Armando Rossello,*[1] *Salvatore Santamaria,*[2,#,]* *Rens de Groot,* [3,#,]* *and Elisa Nuti*[1,#,]*

[1] Department of Pharmacy, University of Pisa, Via Bonanno 6, 56126 Pisa, Italy

[2] Department of Biochemical and Physiological Sciences, School of Biosciences, Faculty of Health and Medical Sciences, Edward Jenner Building, University of Surrey, Guildford GU2 7XH, UK

[3] Institute of Cardiovascular Science, University College London, 51 Chenies Mews, London WC1E 6HX, UK.

*Correspondence: Salvatore Santamaria, e-mail: s.santamaria@surrey.ac.uk; Rens de Groot, e-mail: R.deGroot@ucl.ac.uk; Elisa Nuti: e-mail: elisa.nuti@unipi.it

[¶] D.C. and T.B. contributed equally to this work.

[#] E.N. and RdG and S.S. contributed equally to this work.

Table of Contents

I. Representative NMR spectra of final compounds (1, 2, 3, 3a-g, and 4) **S2**

II. Representative HRMS spectra of final compounds (1, 2, 3, 3a-g, and 4) **S13**

III. Representative NMR spectra of intermediate compounds **S20**

IV. Figure S1 **S43**

V. Figure S2 **S43**

I. Representative NMR spectra of final compounds (1, 2, 3, 3a-g, and 4)

Compound **1**: ^{1}H NMR (400 MHz, DMSO-d_6)

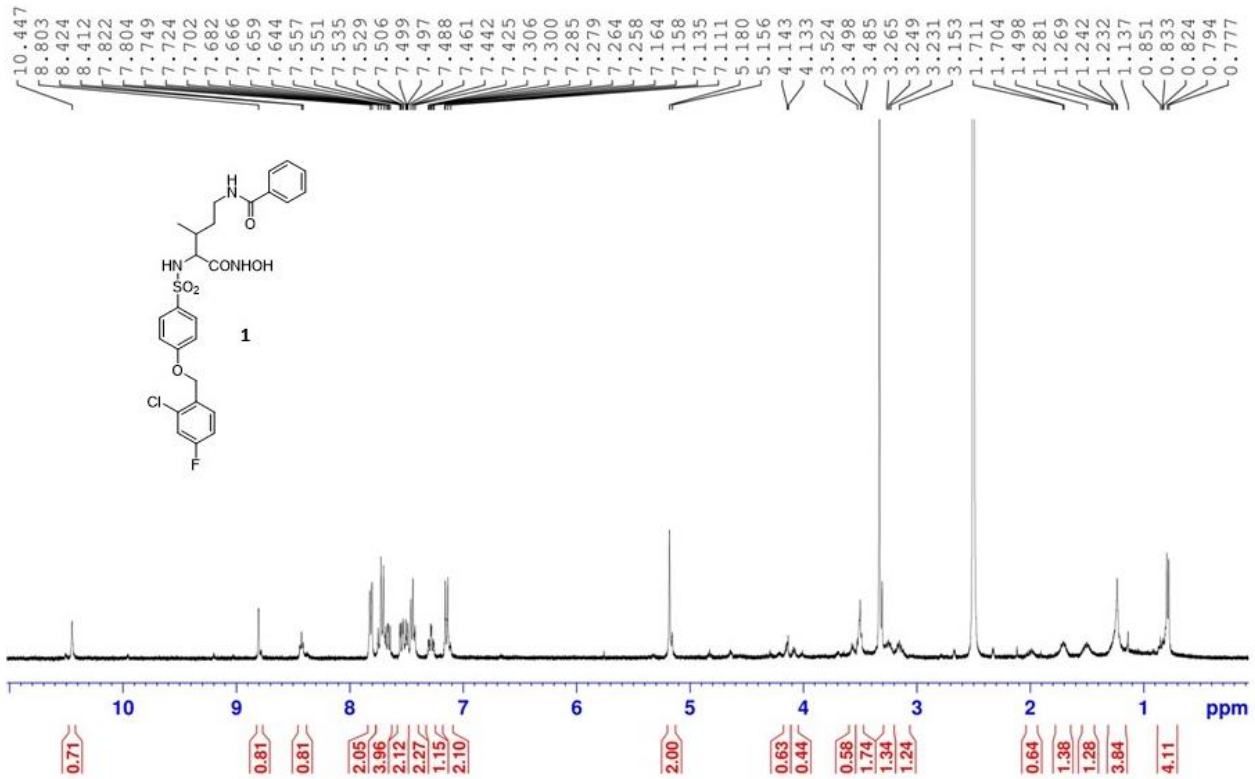

Compound **1:** ^{13}C NMR (CD_3OD)

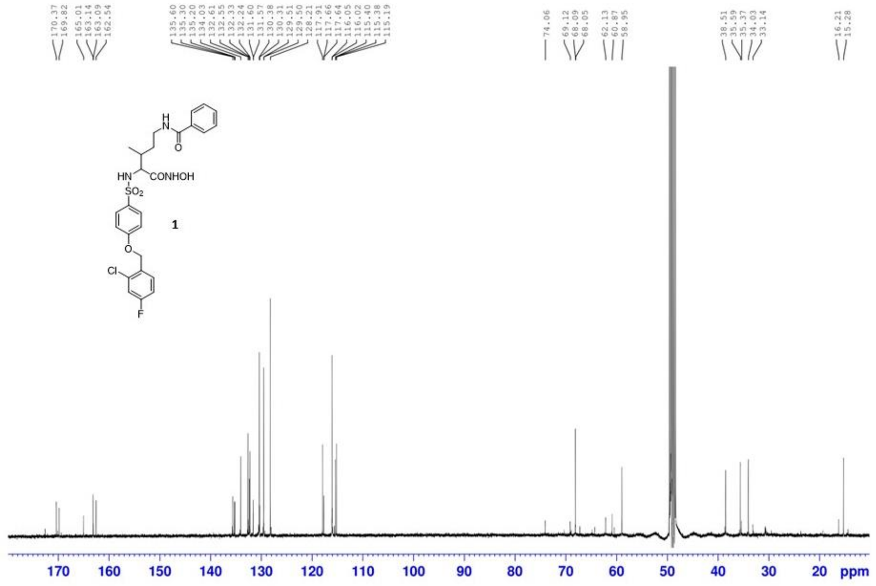

Compound **2**: ^{1}H NMR (400 MHz, CD_3OD)

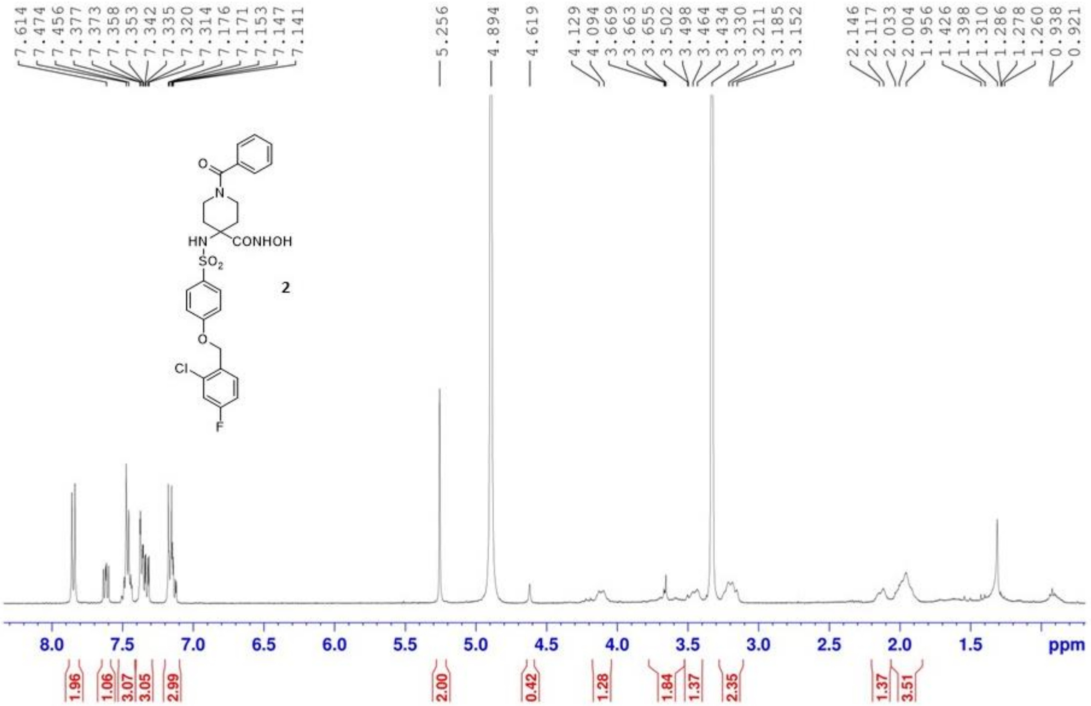

Compound **2**: ^{13}C NMR (100 MHz, CD_3OD)

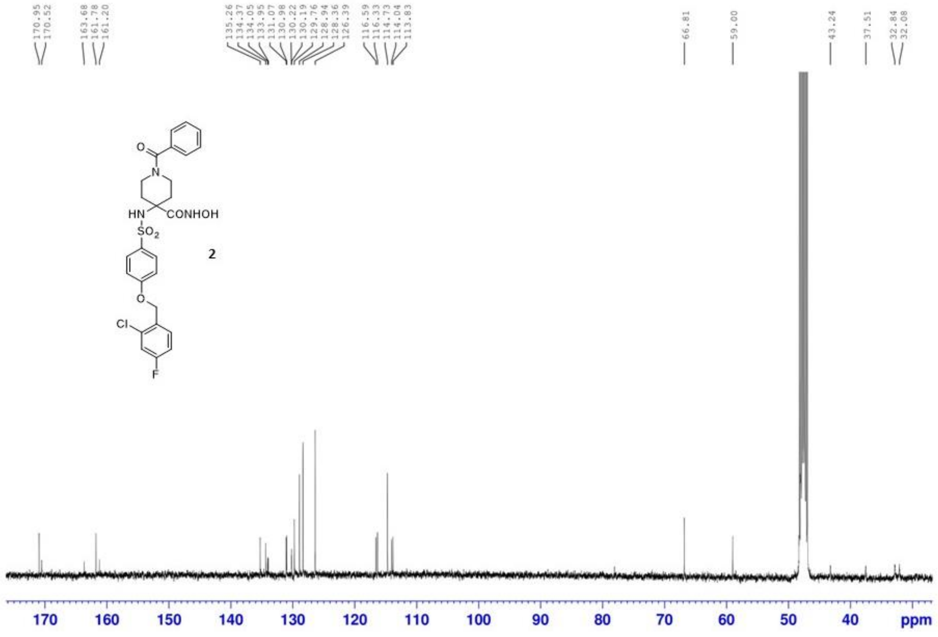

Compound **3**: ^{1}H NMR (400 MHz, CD_3OD).

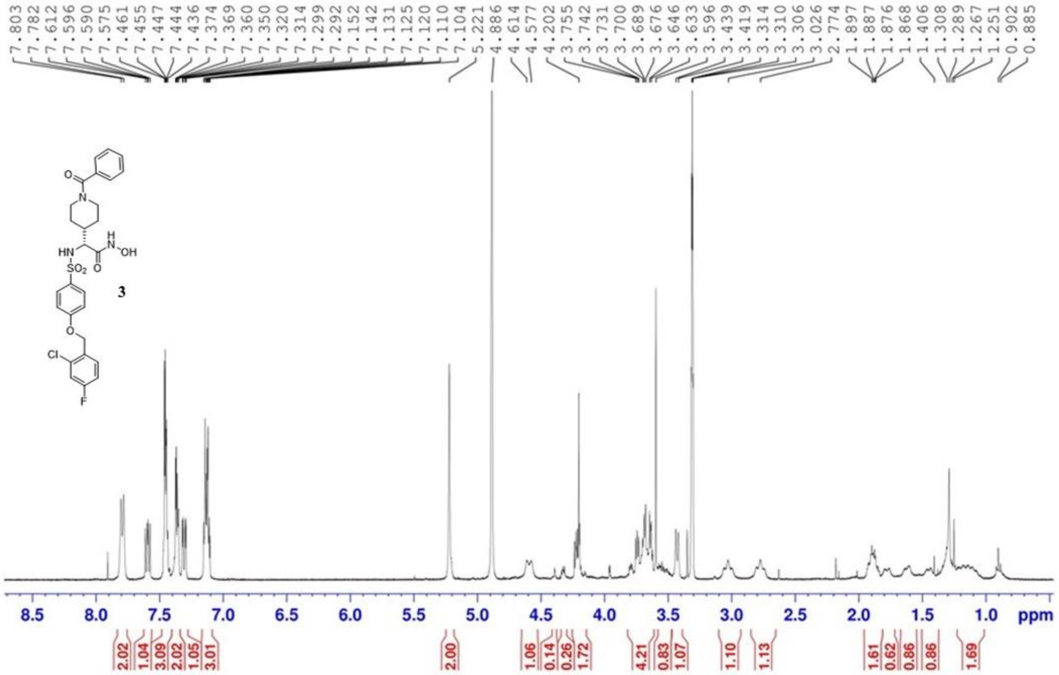

Compound **3**: ^{13}C NMR (100 MHz, CD_3OD).

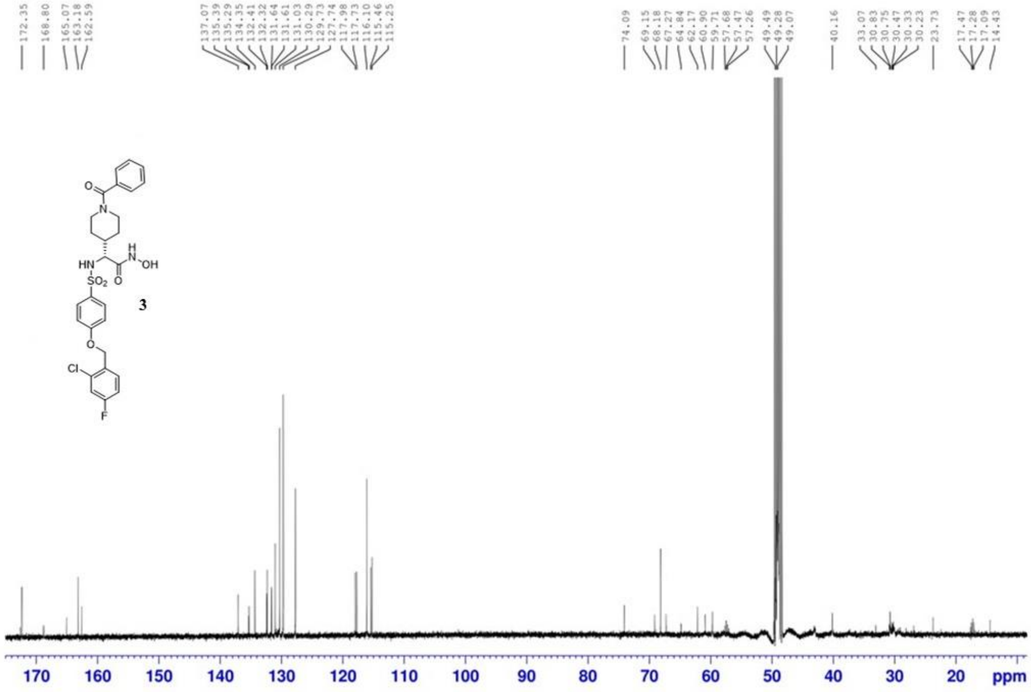

Compound **3a**: ^{1}H NMR (400 MHz, DMSO-d_6).

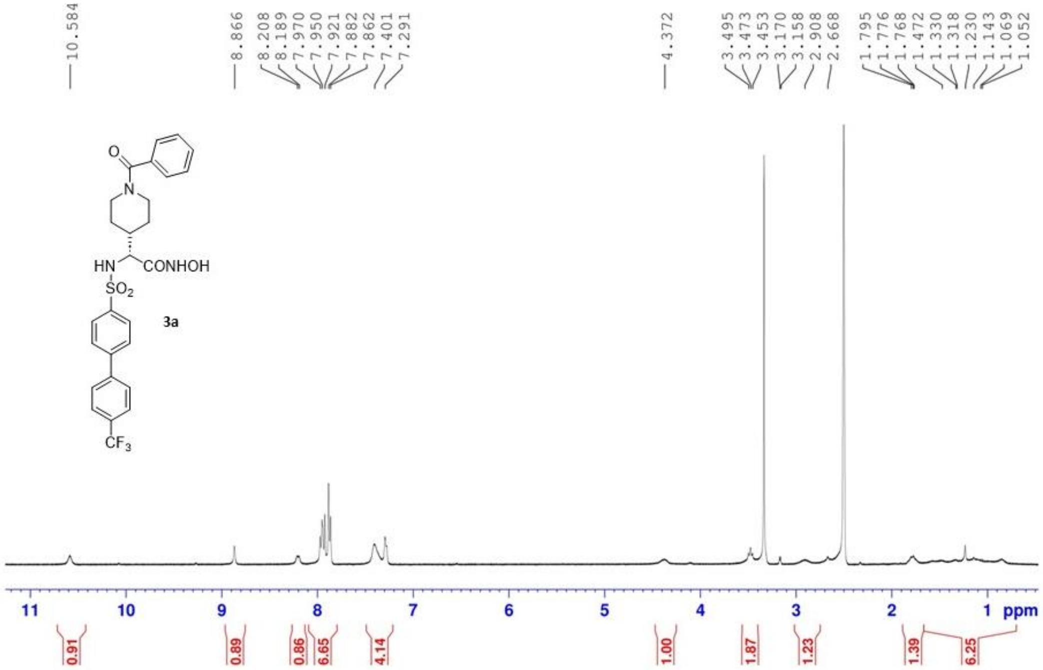

Compound **3a**: ^{13}C NMR (100 MHz, DMSO-d_6).

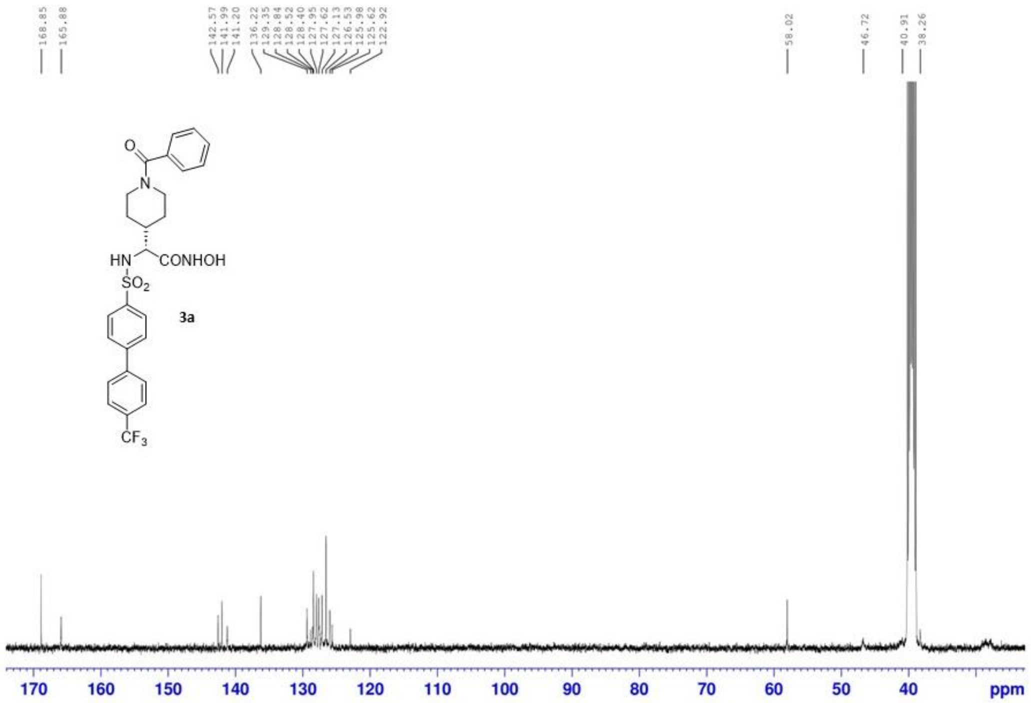

Compound **3b**: ^{1}H NMR (400 MHz, $CDCl_3$).

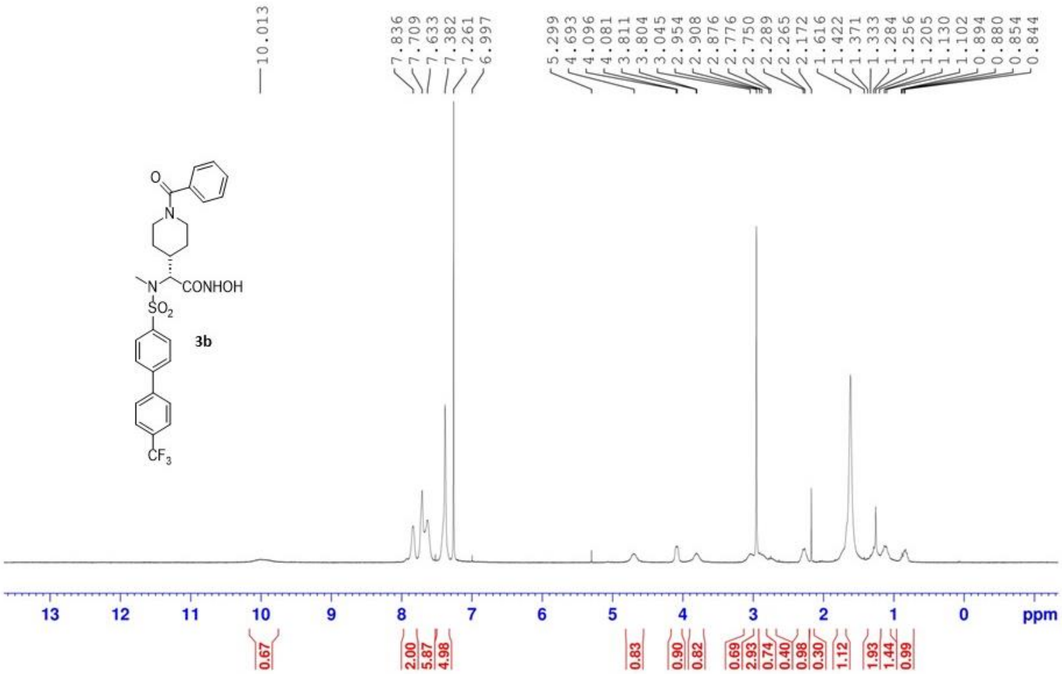

Compound **3b**: ^{13}C NMR (100 MHz, $CDCl_3$).

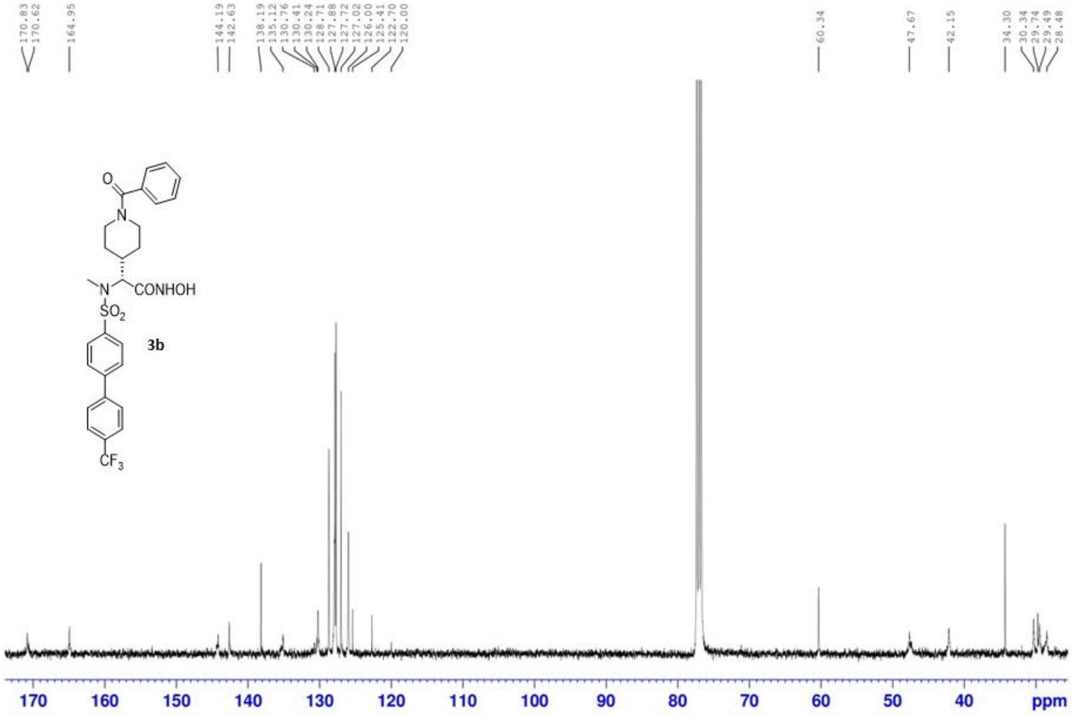

Compound **3c**: ^{1}H NMR (400 MHz, CD_3OD).

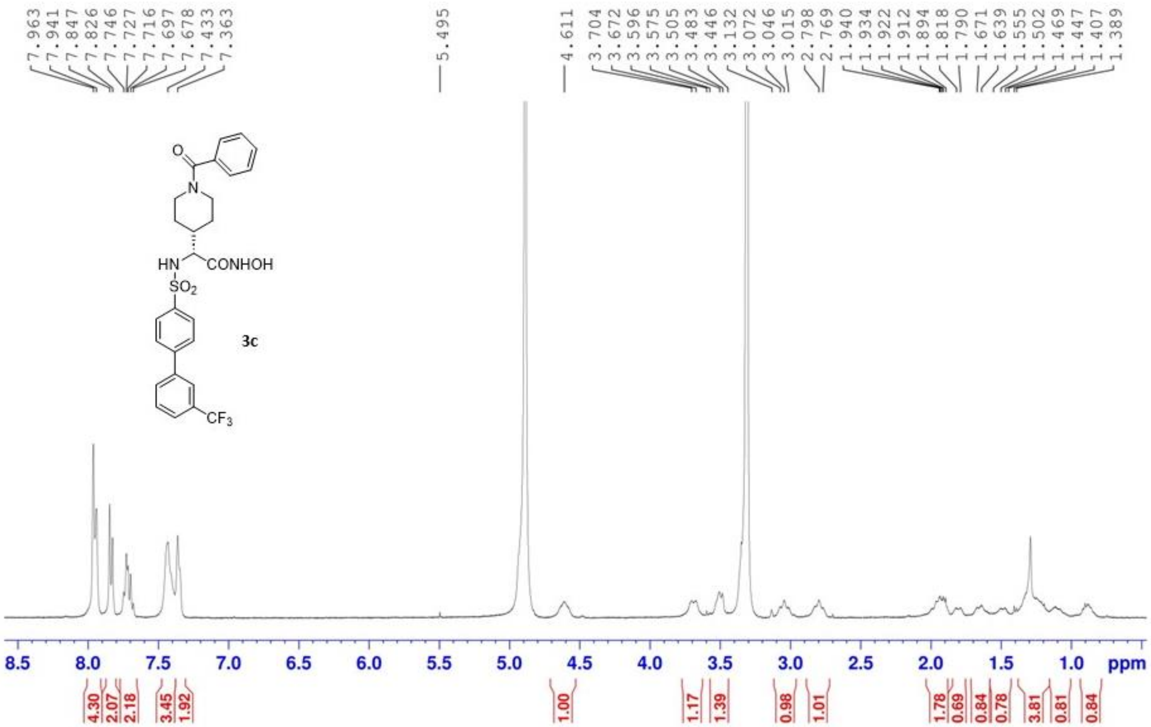

Compound **3c**: ^{13}C NMR (100 MHz, CD_3OD).

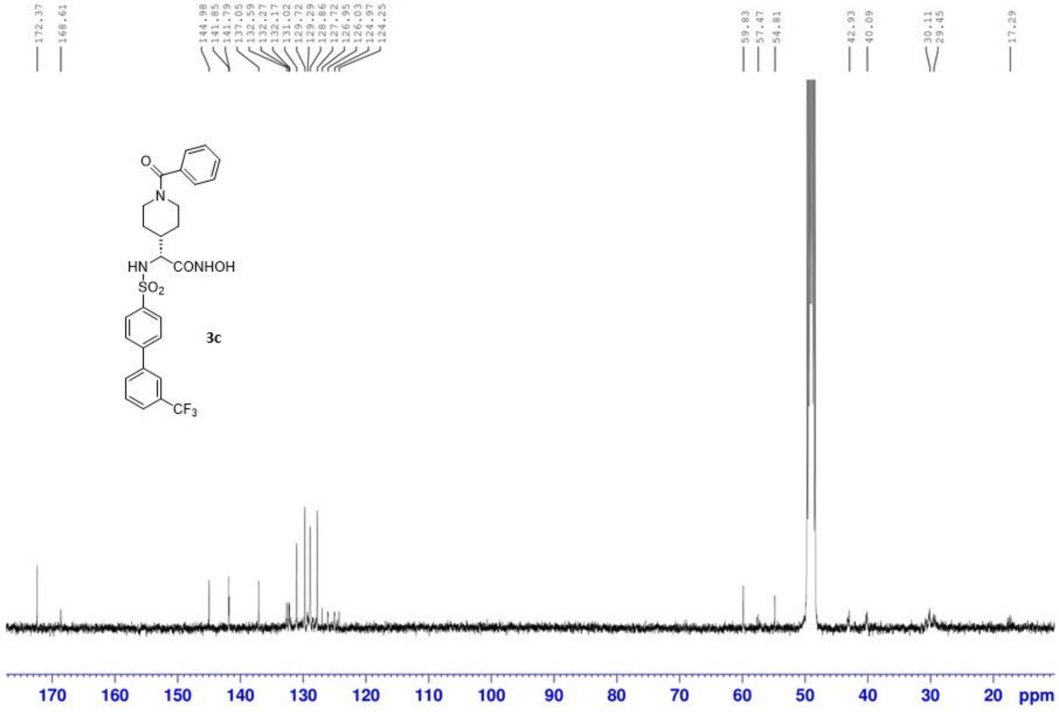

Compound **3d**: ^{1}H NMR (400 MHz, DMSO-d_6).

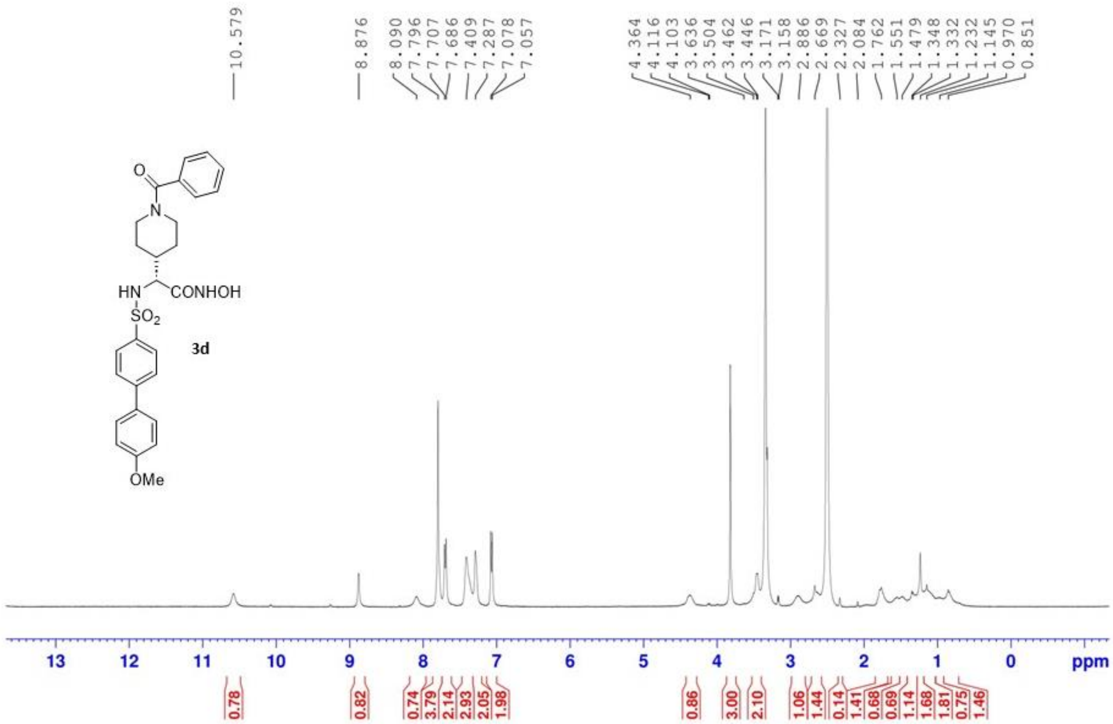

Compound **3d**: ^{13}C NMR (100 MHz, DMSO-d_6).

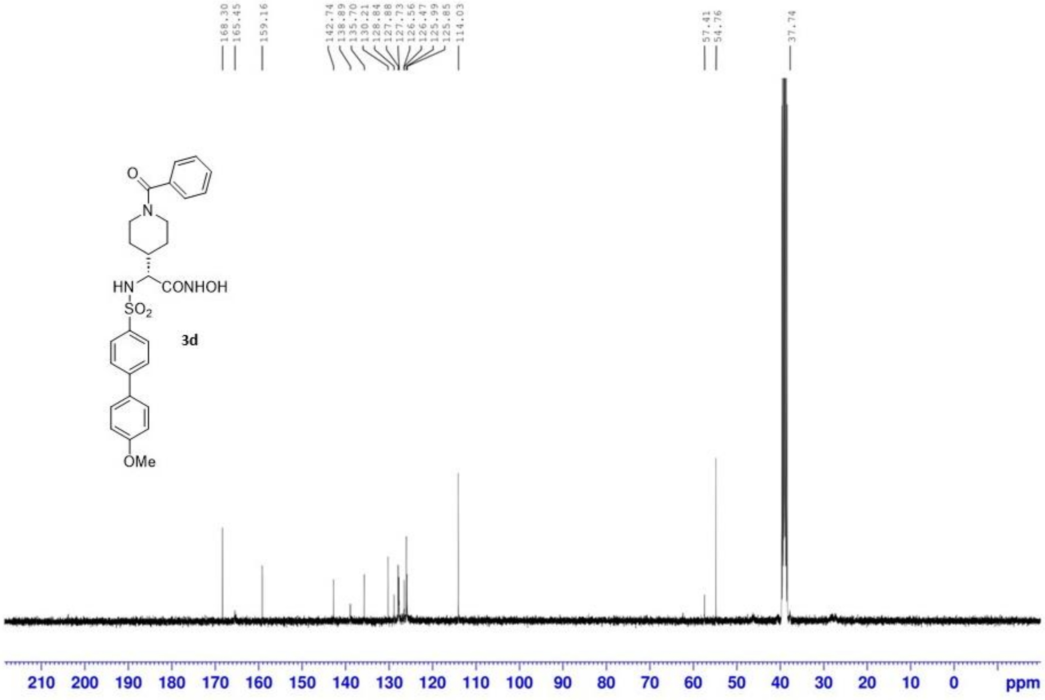

Compound **3e**: ^{1}H NMR (400 MHz, DMSO-d_6).

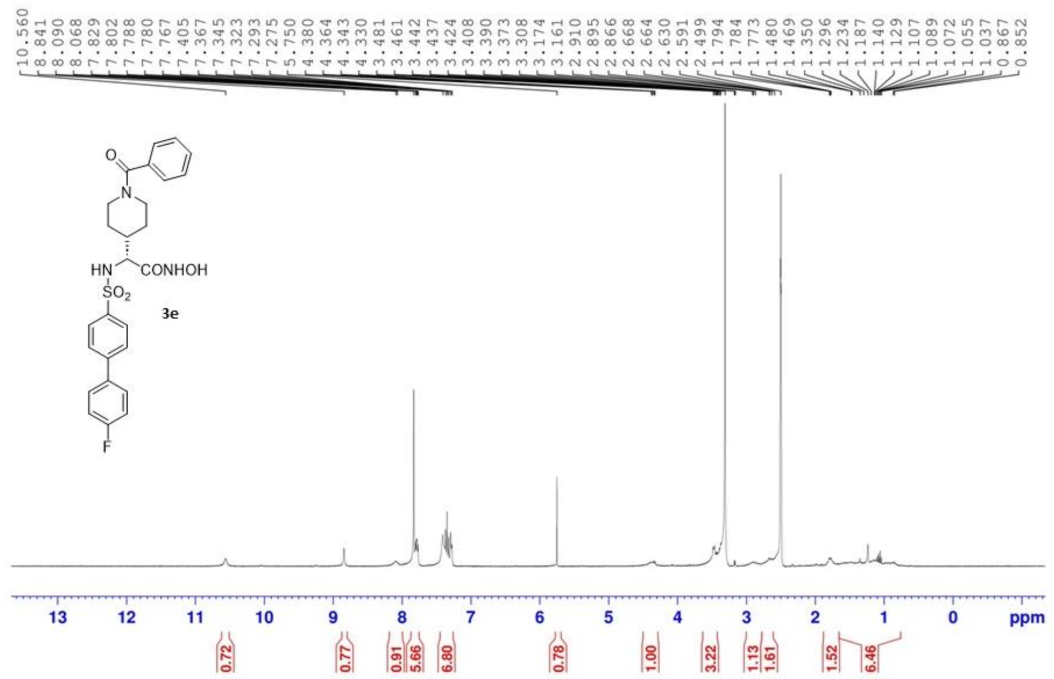

Compound **3e**: ^{13}C NMR (100 MHz, CD_3OD).

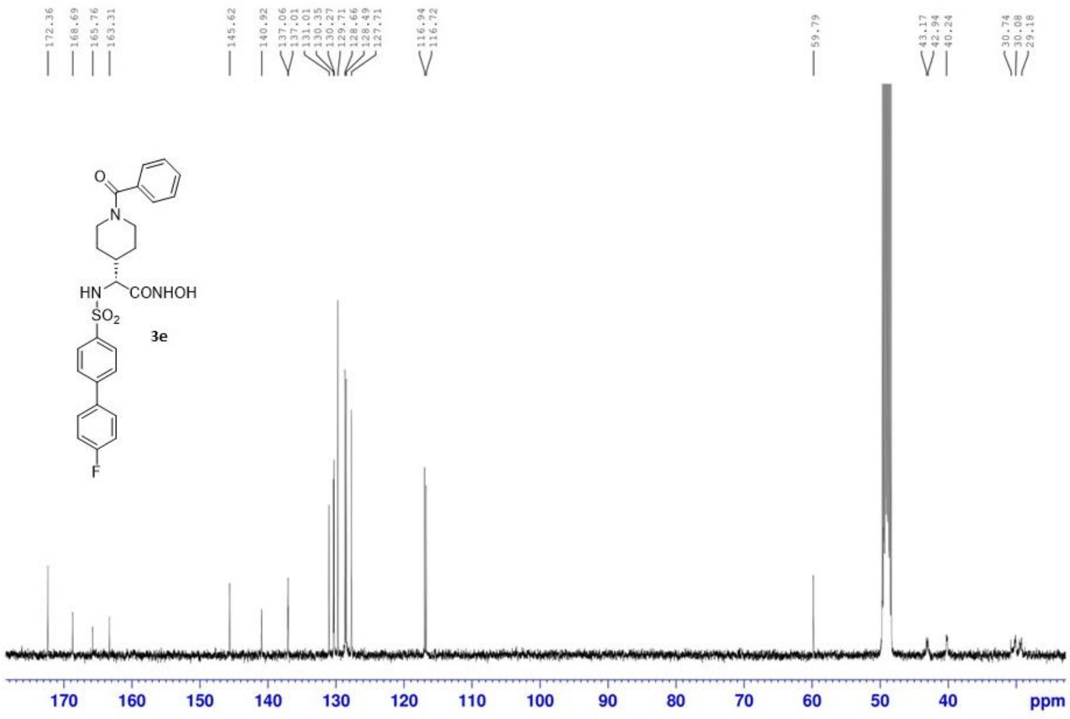

Compound **3f**: ^{1}H NMR (400 MHz, DMSO-d_6).

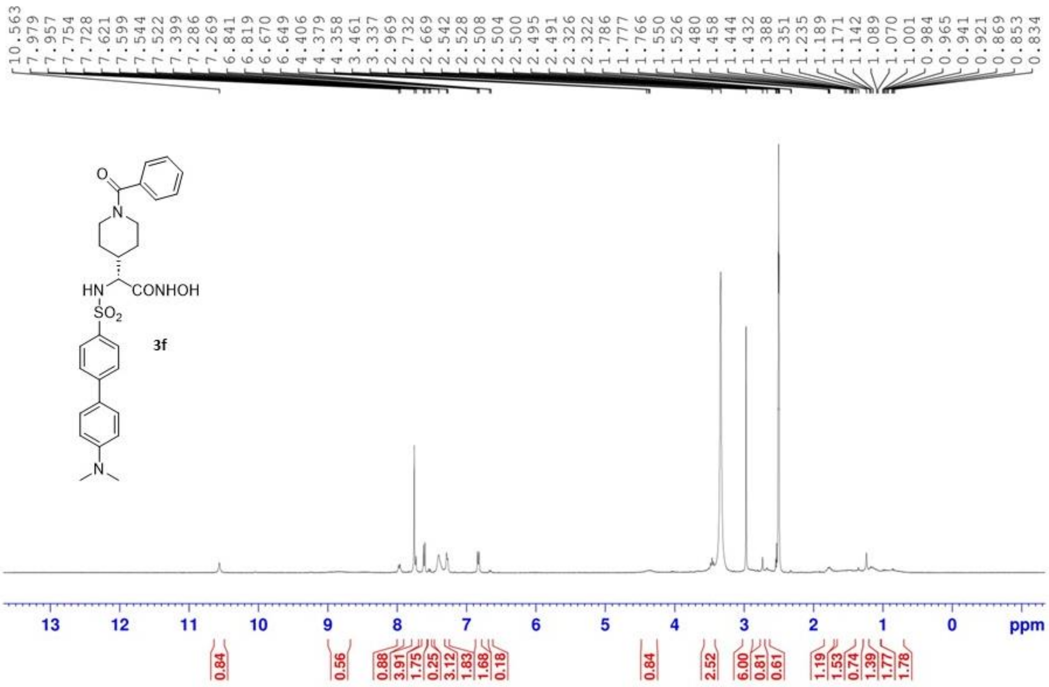

Compound **3f**: ^{13}C NMR (100 MHz, DMSO-d_6).

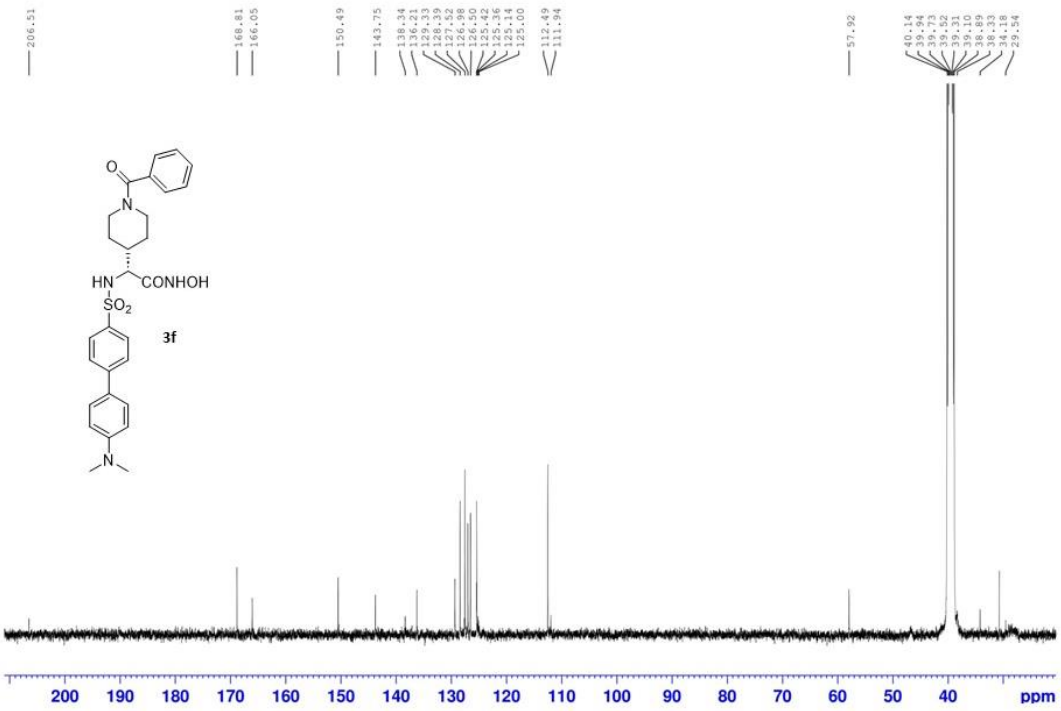

Compound **3g**: ^{1}H NMR (400 MHz, DMSO-d_6).

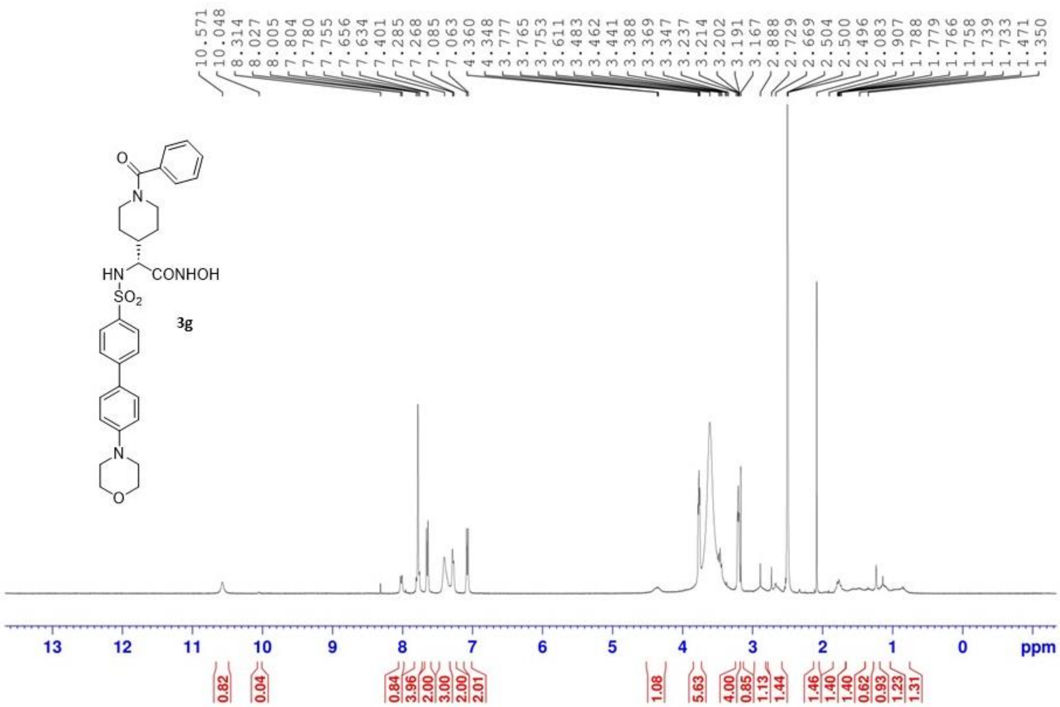

Compound **3g**: ^{13}C NMR (100 MHz, DMSO-d_6).

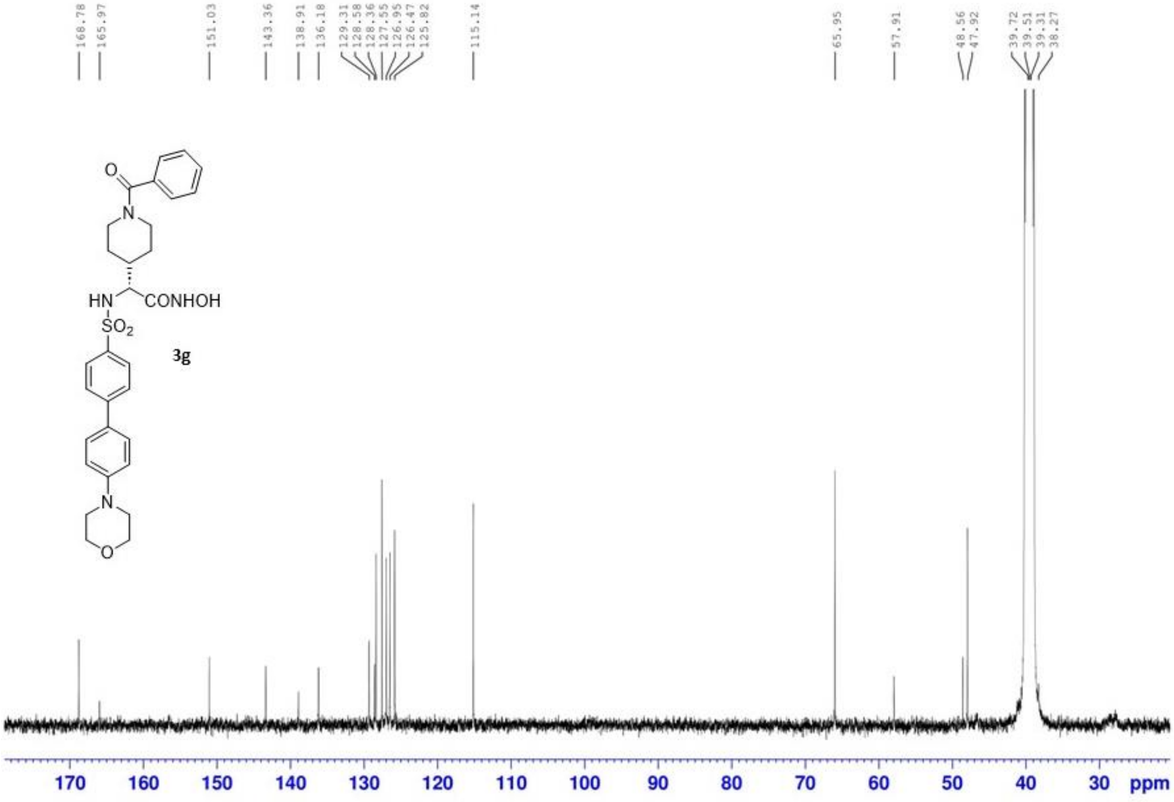

Compound **4**: ^{1}H NMR (400 MHz, CD_3OD).

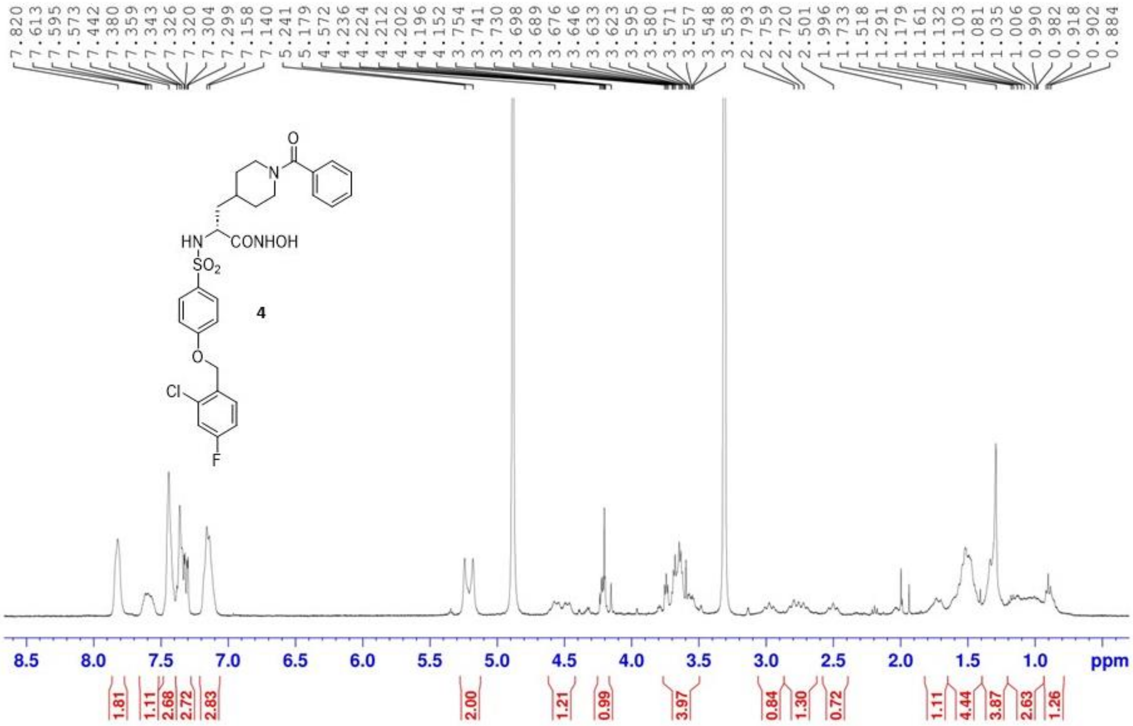

Compound **4**: ^{13}C NMR (100 MHz, CD_3OD).

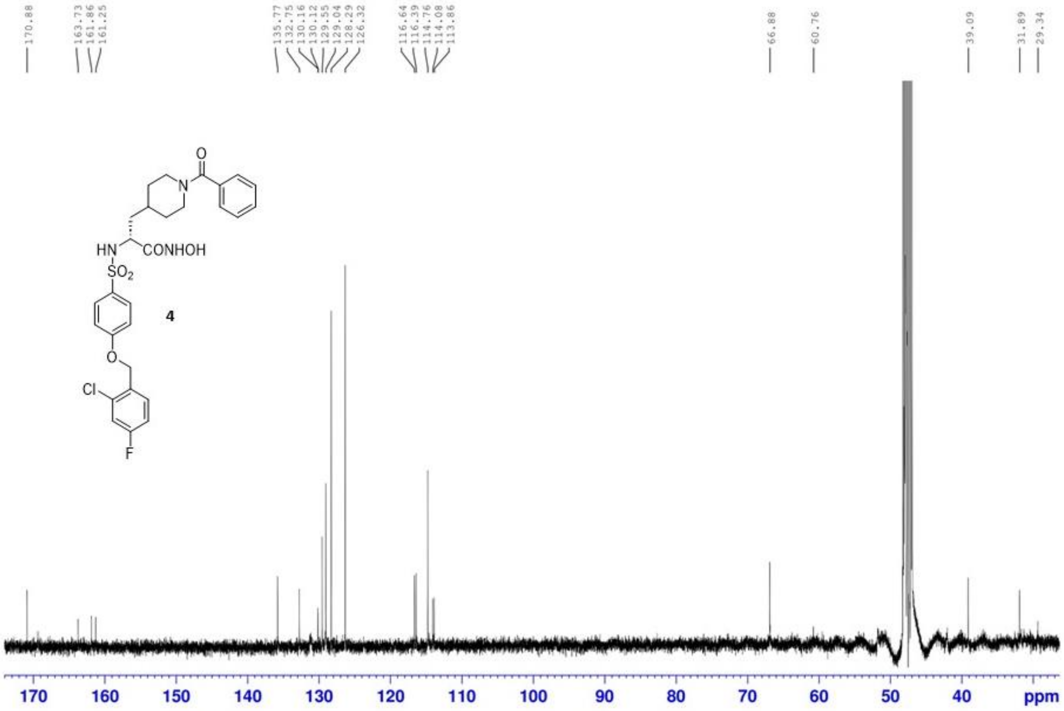

II. Representative HRMS spectra of final compounds (1, 2, 3, 3a-g, and 4)

HRMS spectrum of compound **1**

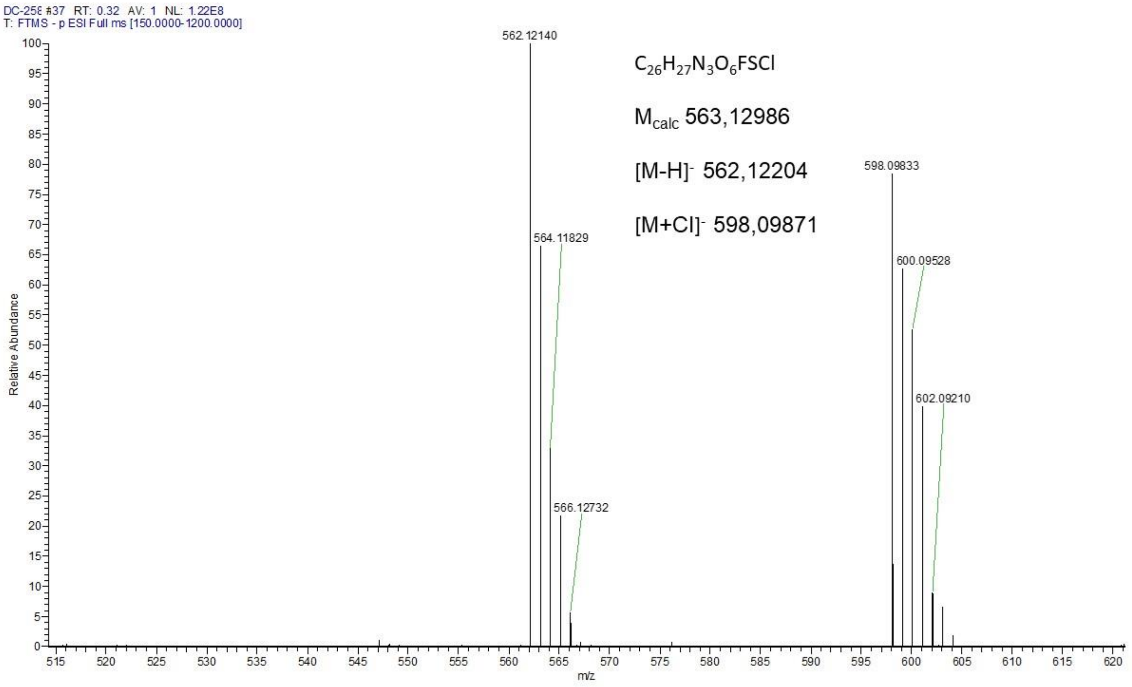

HRMS spectrum of compound **2**

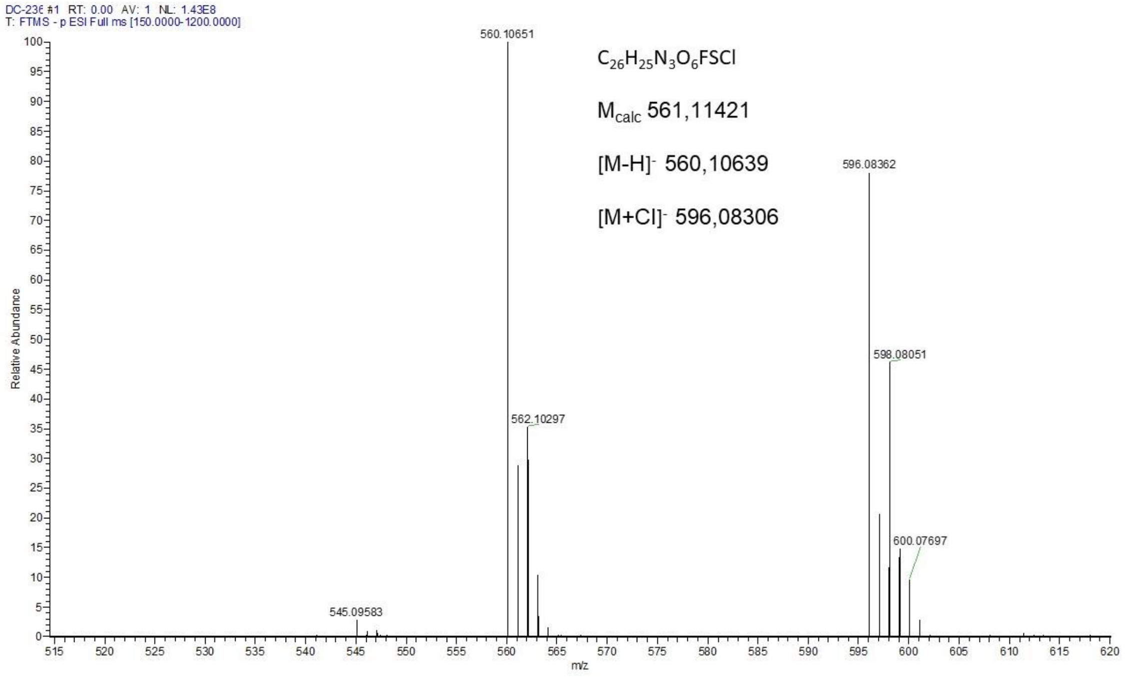

HRMS spectrum of compound **3**

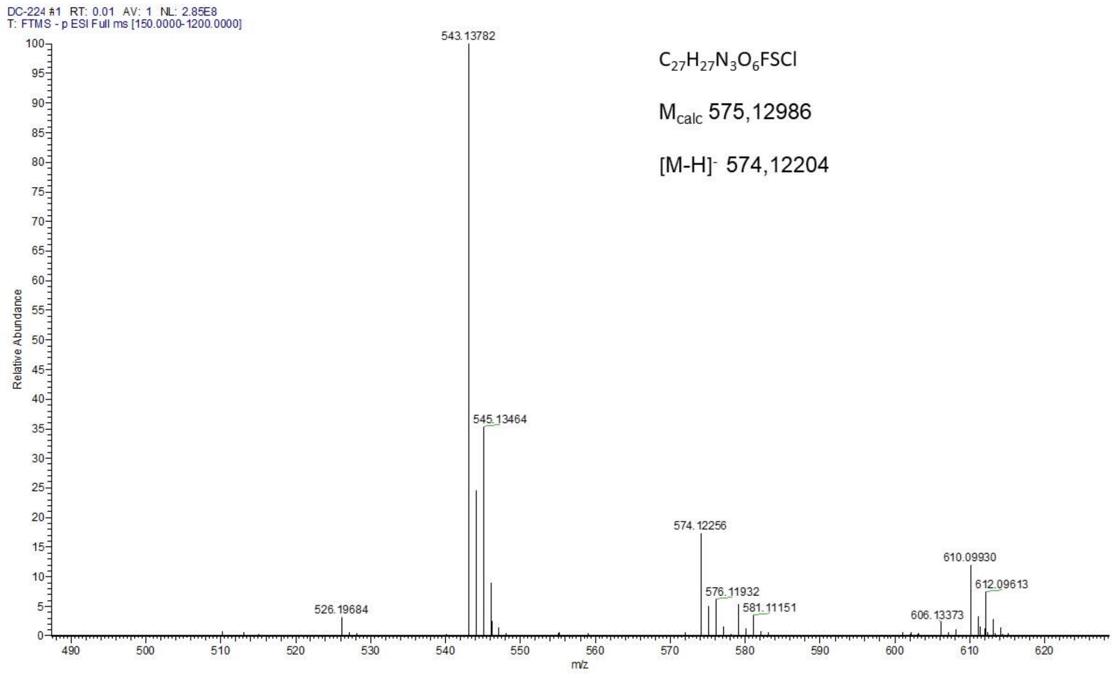

HRMS spectrum of compound **3a**

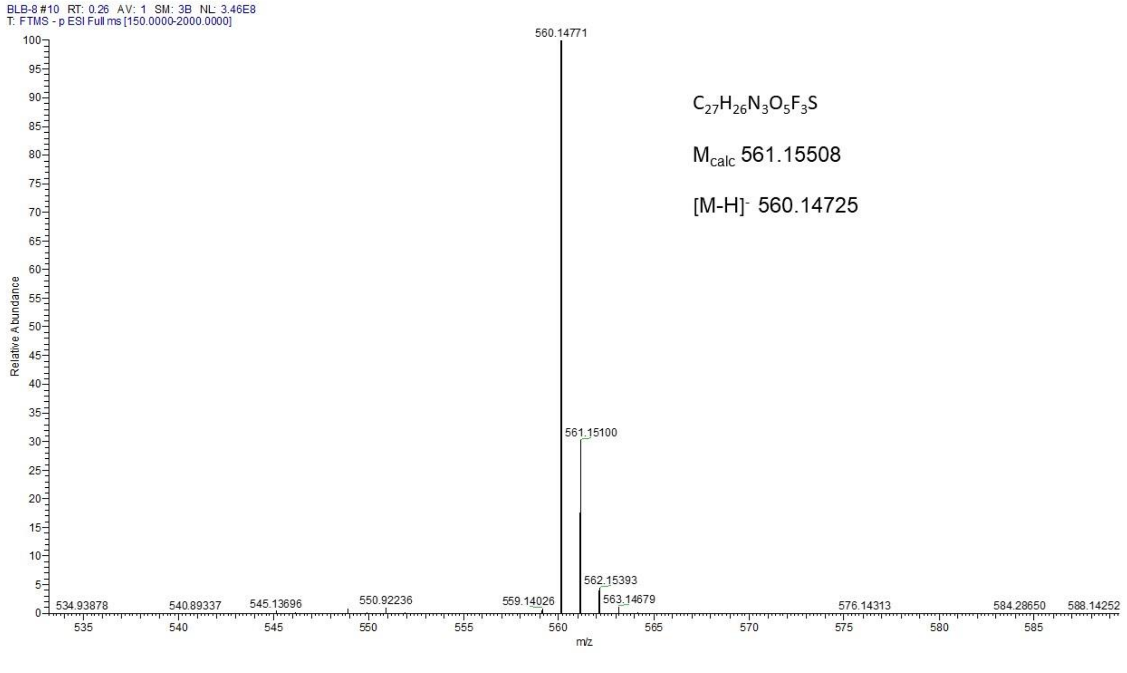

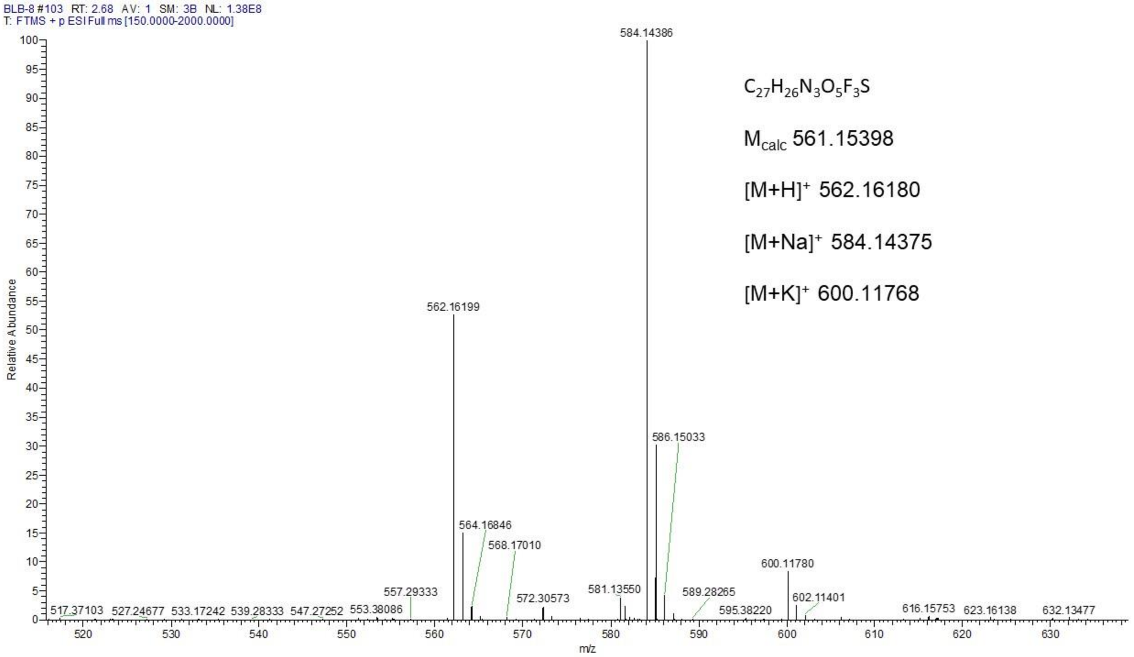

HRMS spectrum of compound **3b**

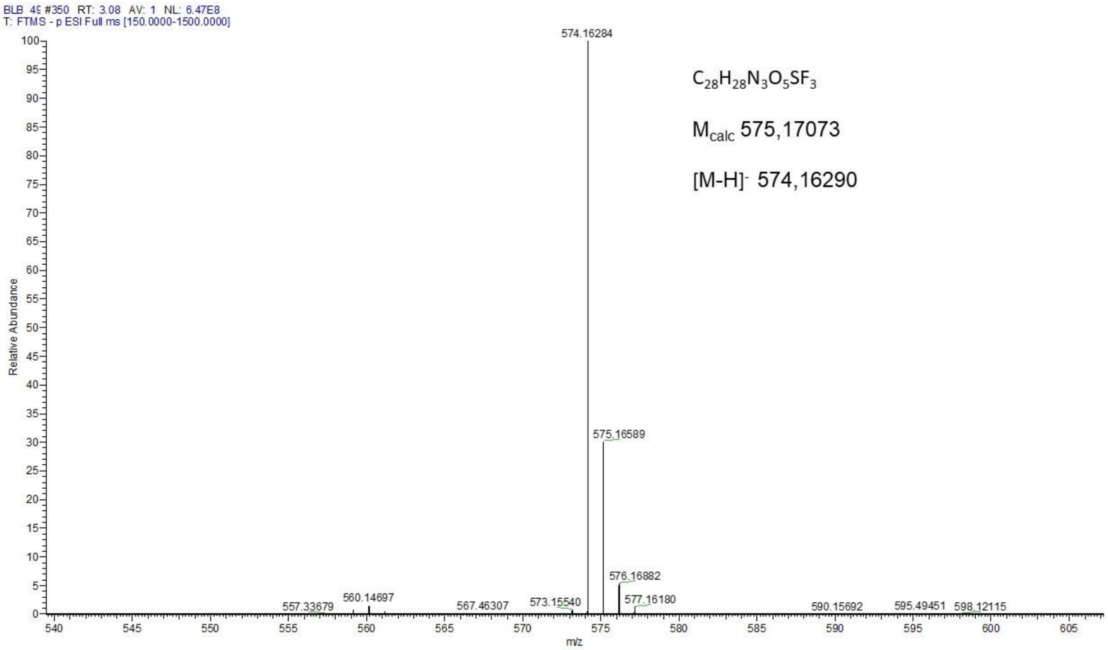

HRMS spectrum of compound **3c**

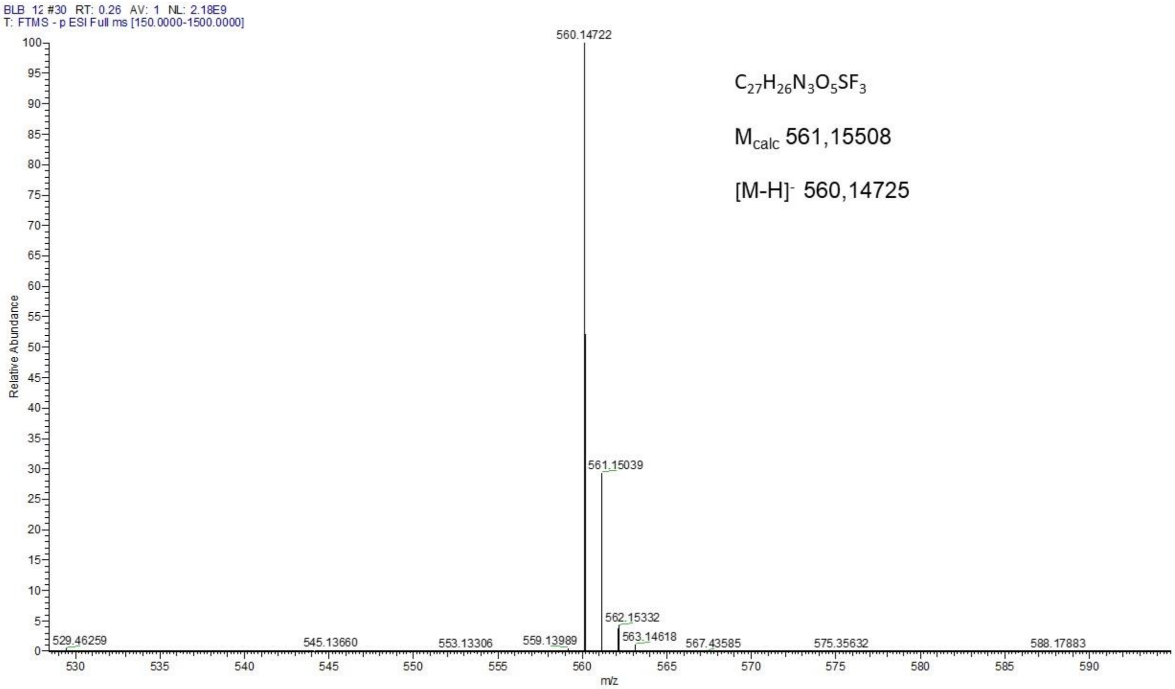

HRMS spectrum of compound **3d**

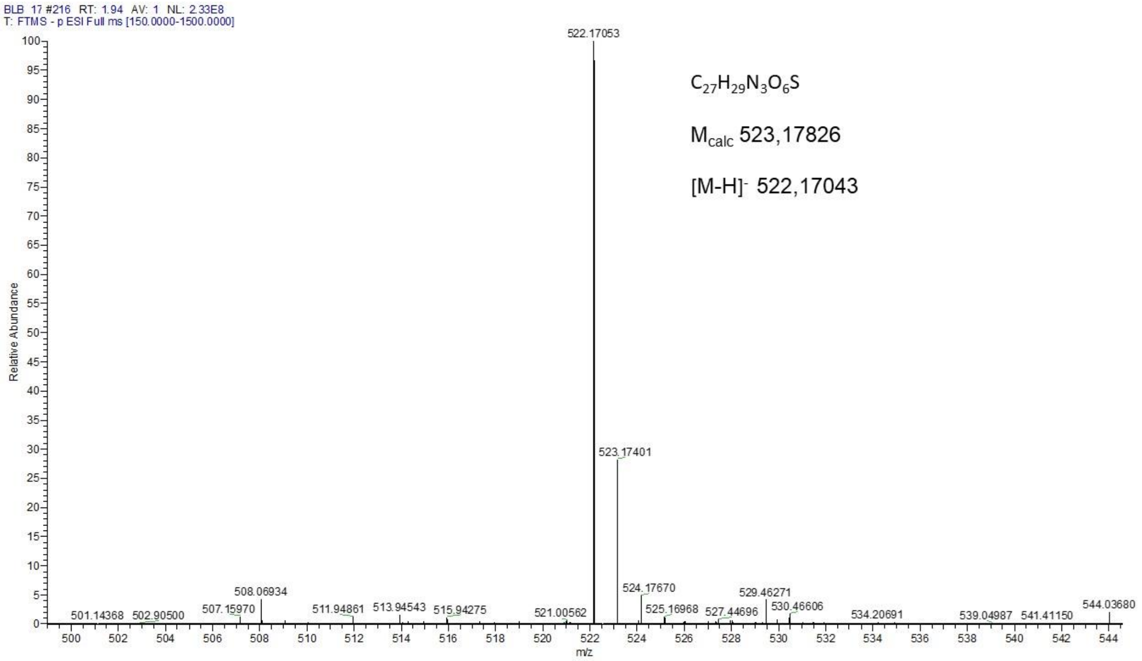

HRMS spectrum of compound **3e**

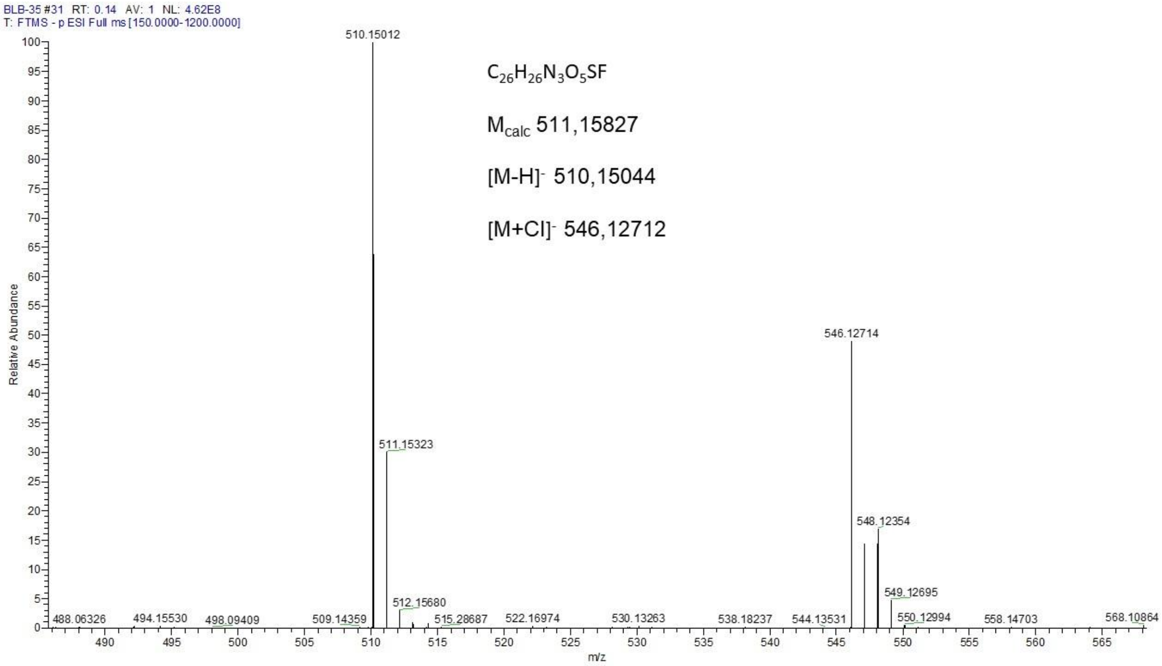

HRMS spectrum of compound **3f**

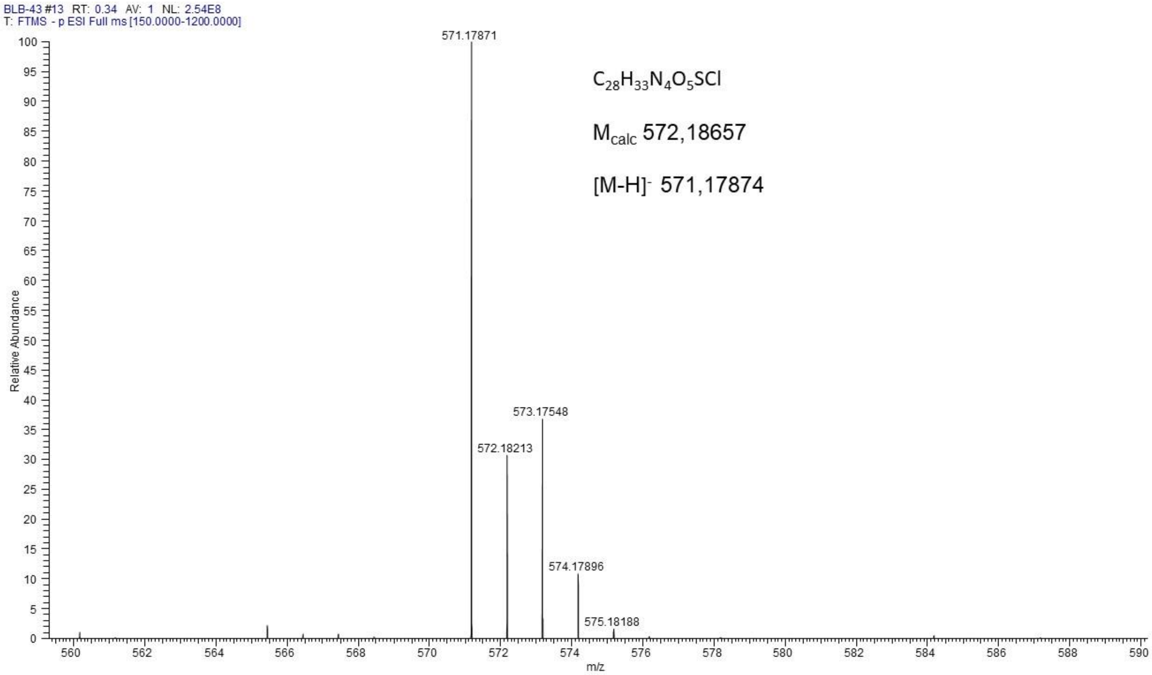

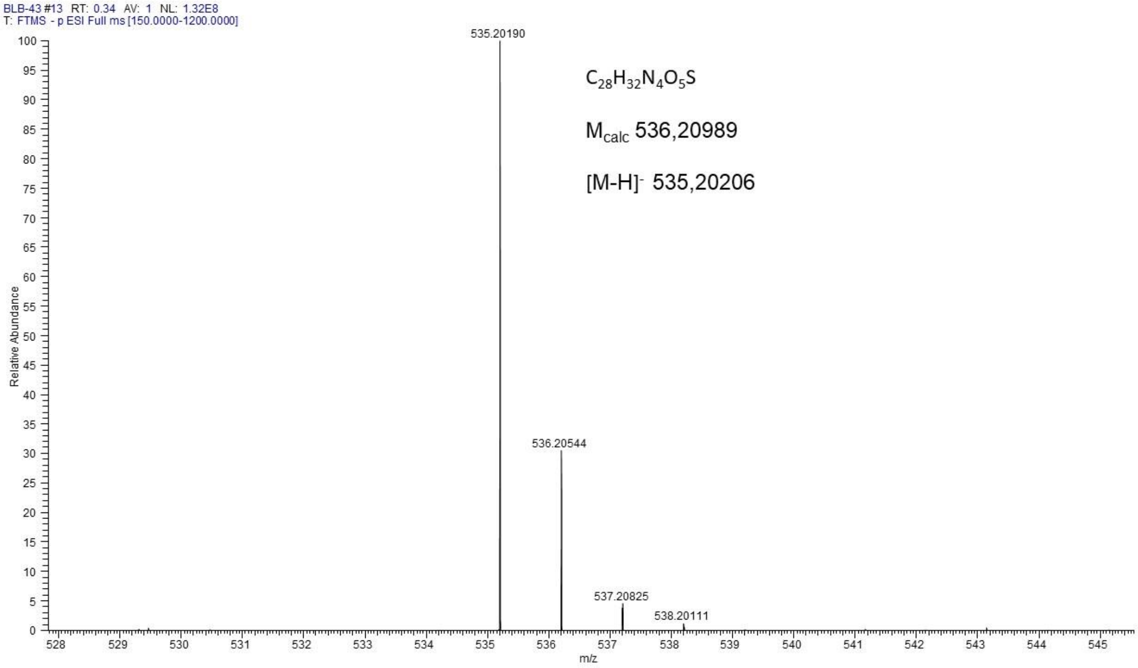

HRMS spectrum of compound **3g**

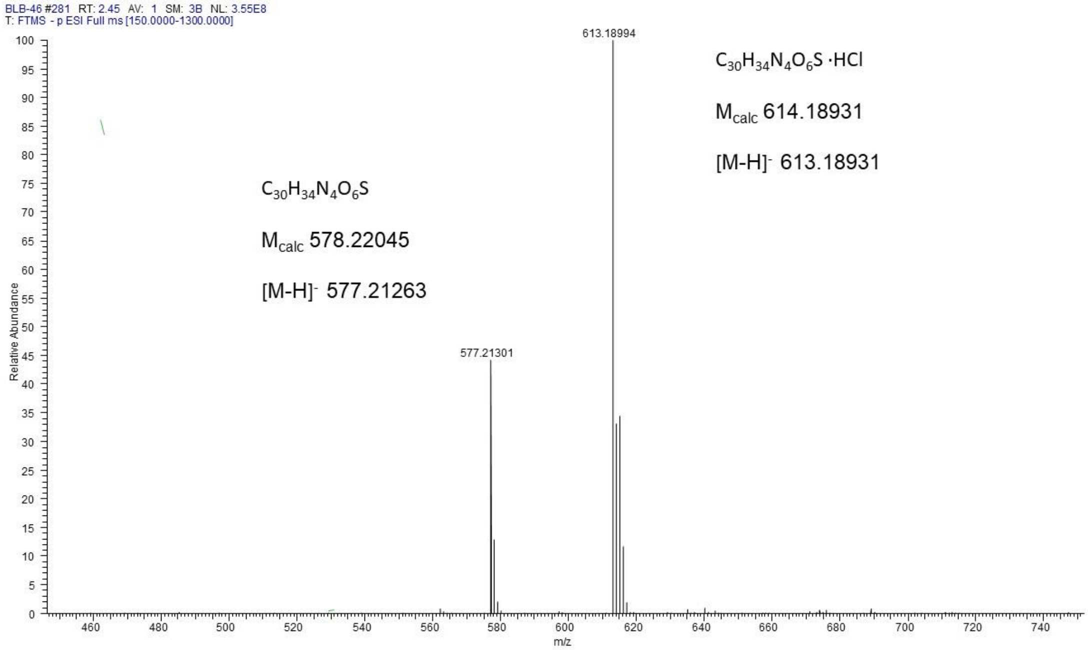

HRMS spectrum of compound **4**

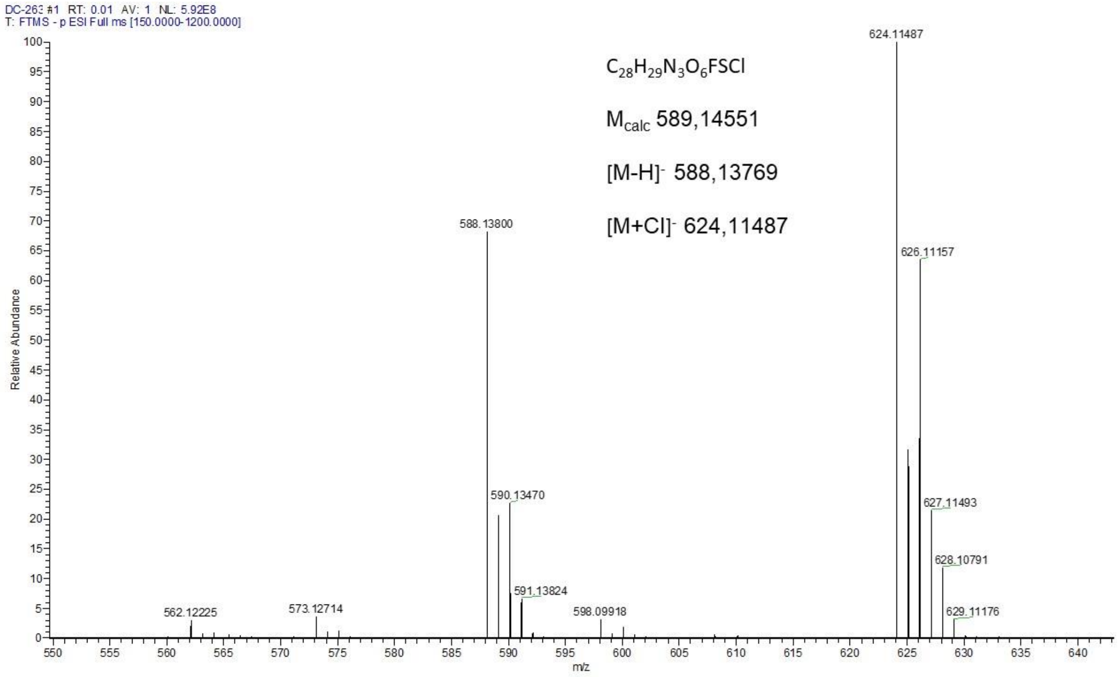

III. Representative NMR spectra of intermediate compounds

Scheme 1: Synthesis of final compound 1

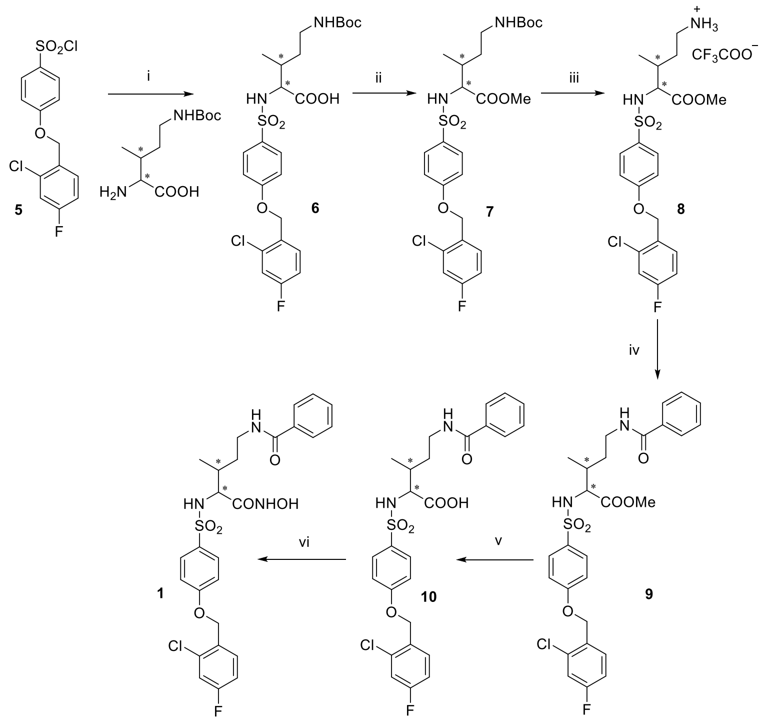

Compound **6**: ^{1}H NMR (400 MHz, DMSO).

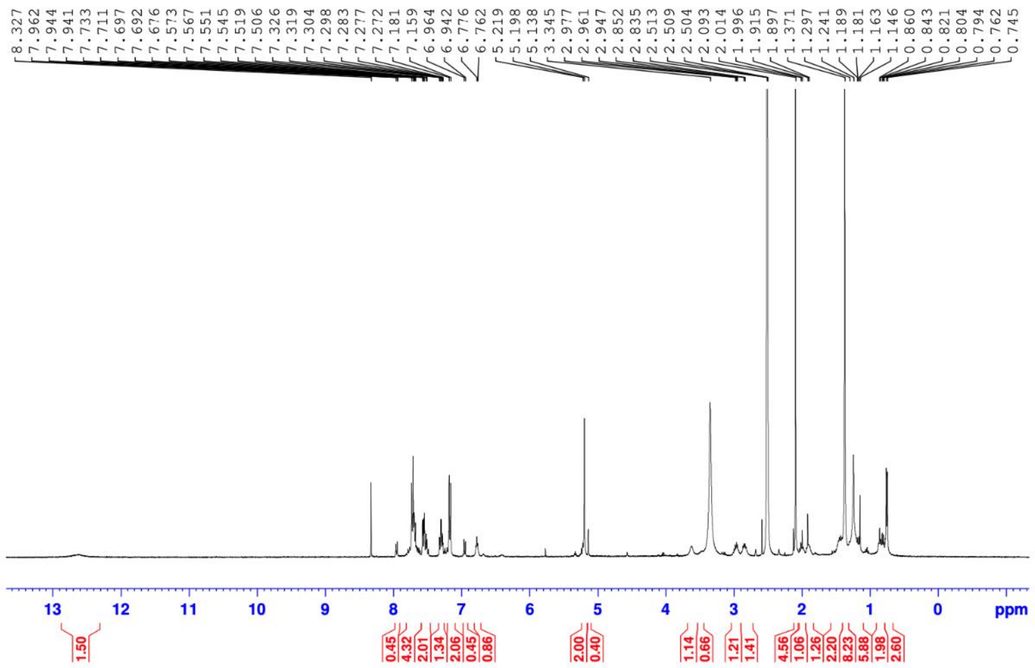

Compound **6**: ^{1}H-^{1}H COSY NMR (400 MHz, DMSO).

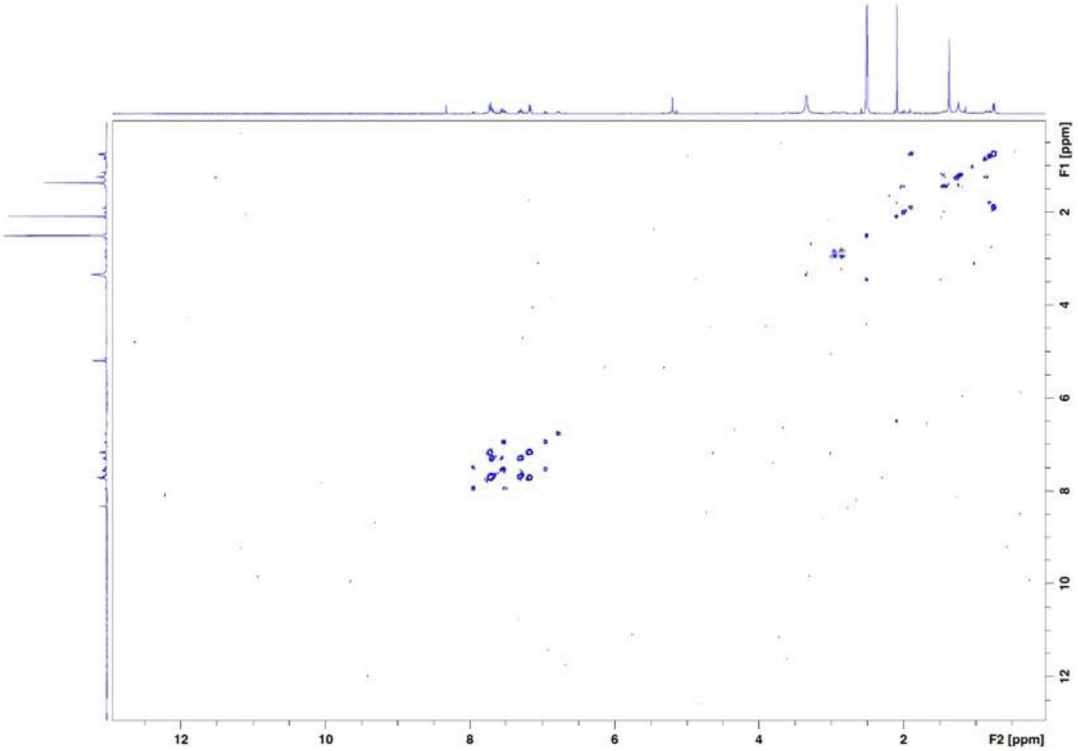

Compound **7**: ^{1}H NMR (400 MHz, $CDCl_3$).

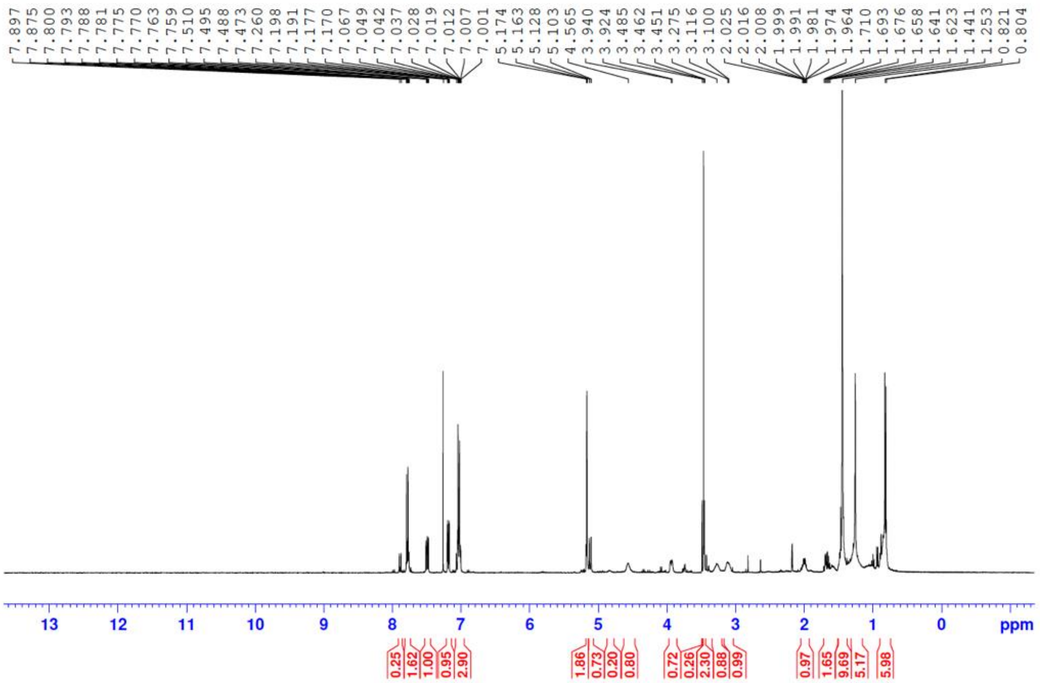

Compound **8**: ^{1}H NMR (400 MHz, DMSO).

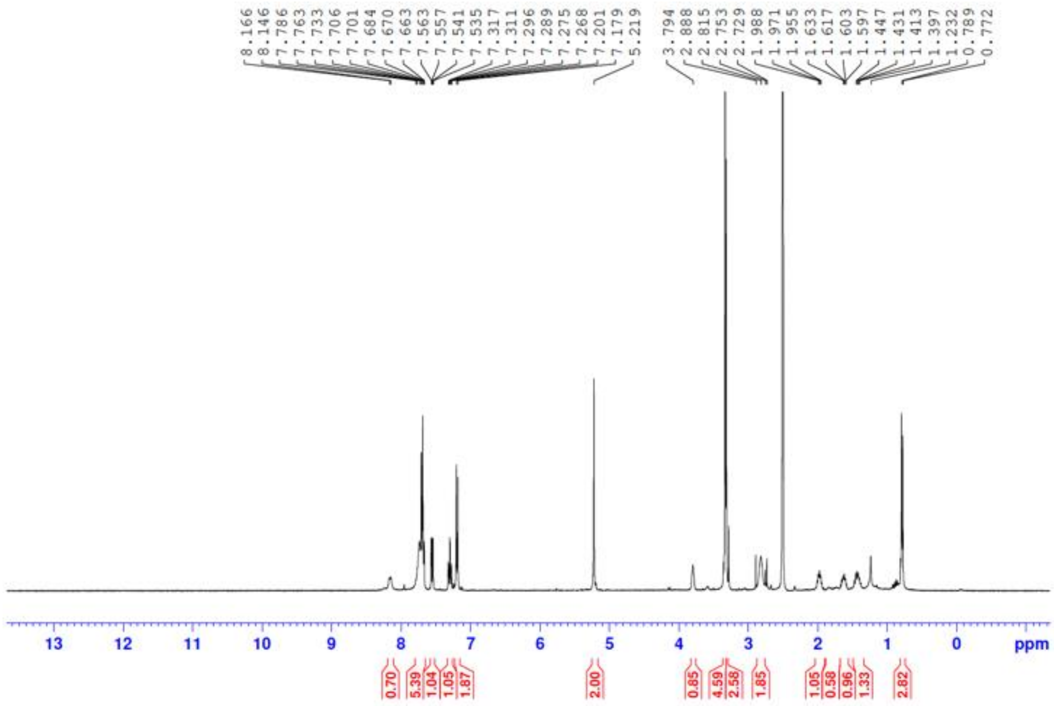

Compound **9**: ^{1}H NMR (400 MHz, $CDCl_3$).

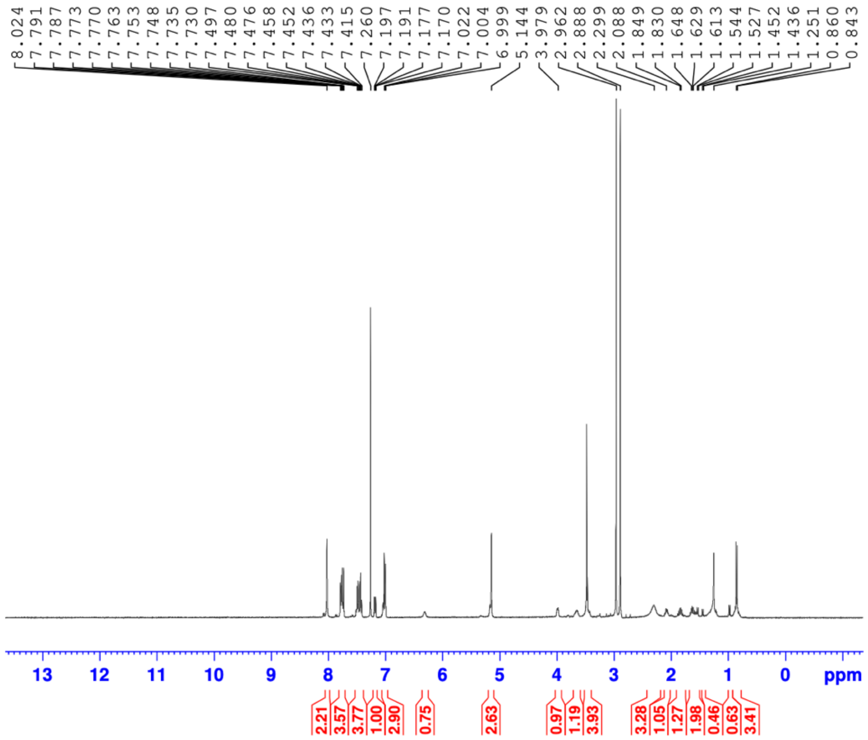

Compound **10**: ^{1}H NMR (400 MHz, DMSO).

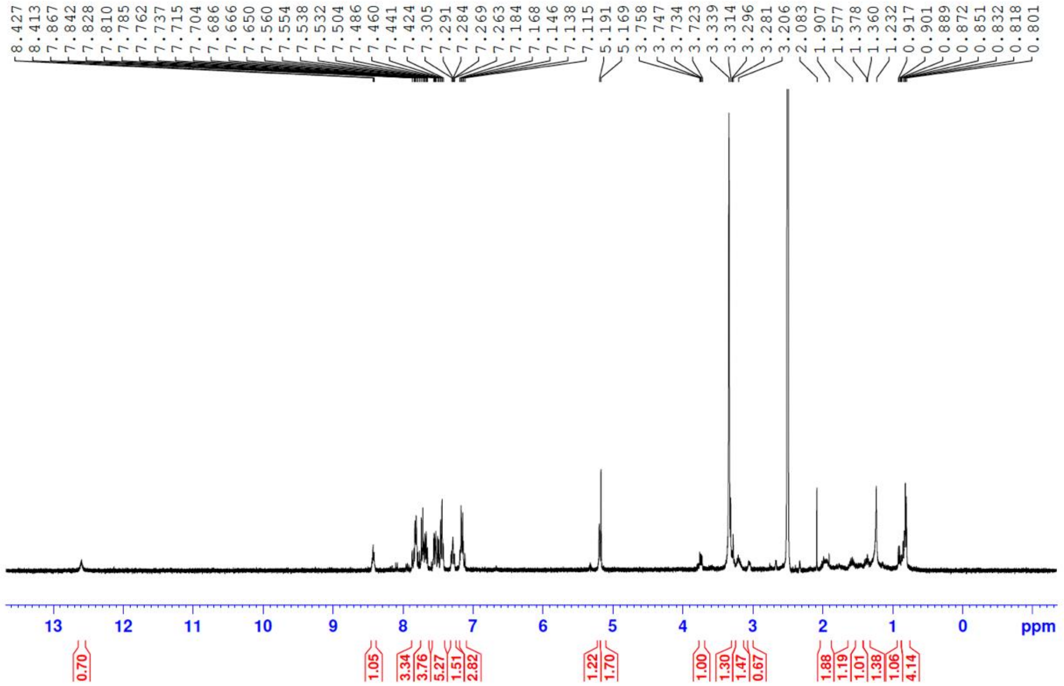

Scheme 2: Synthesis of final compounds 2,4

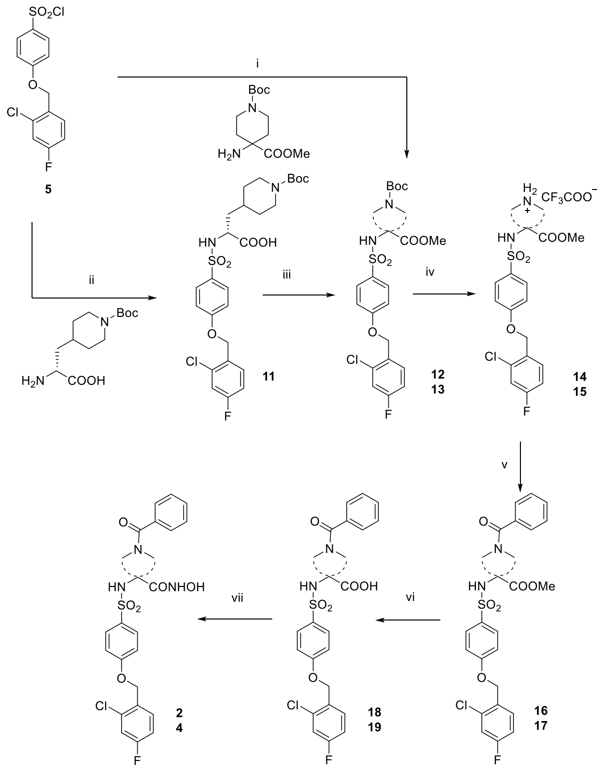

Compound **11**: ^{1}H NMR (400 MHz, $CDCl_3$).

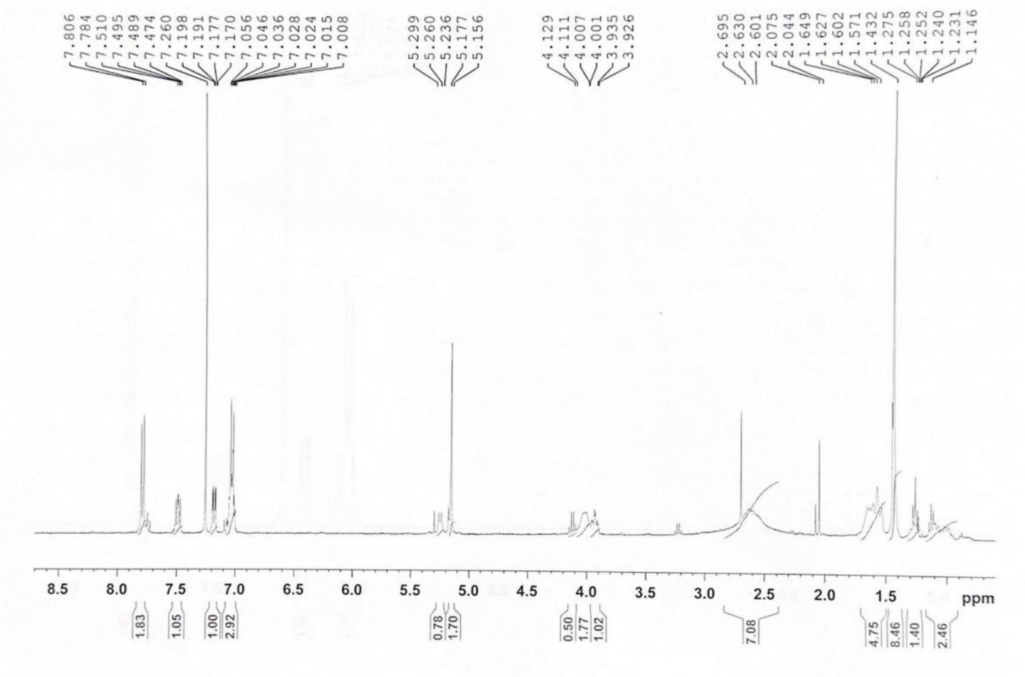

Compound **12**: ^{1}H NMR (400 MHz, $CDCl_3$).

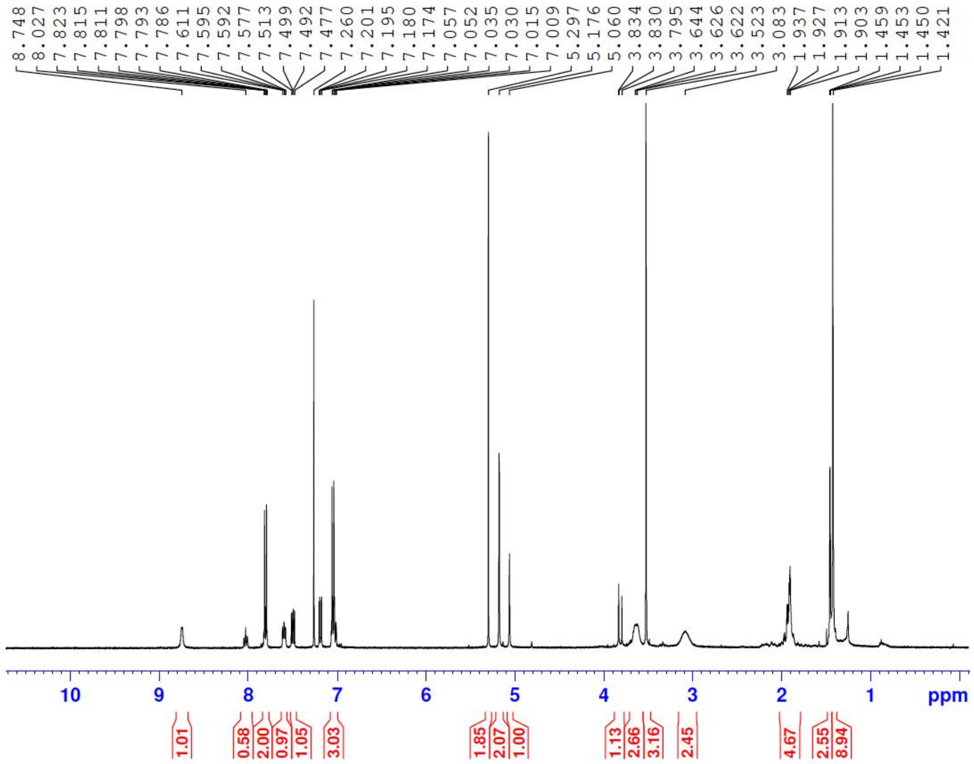

Compound **13**: ^{1}H NMR (400 MHz, $CDCl_3$).

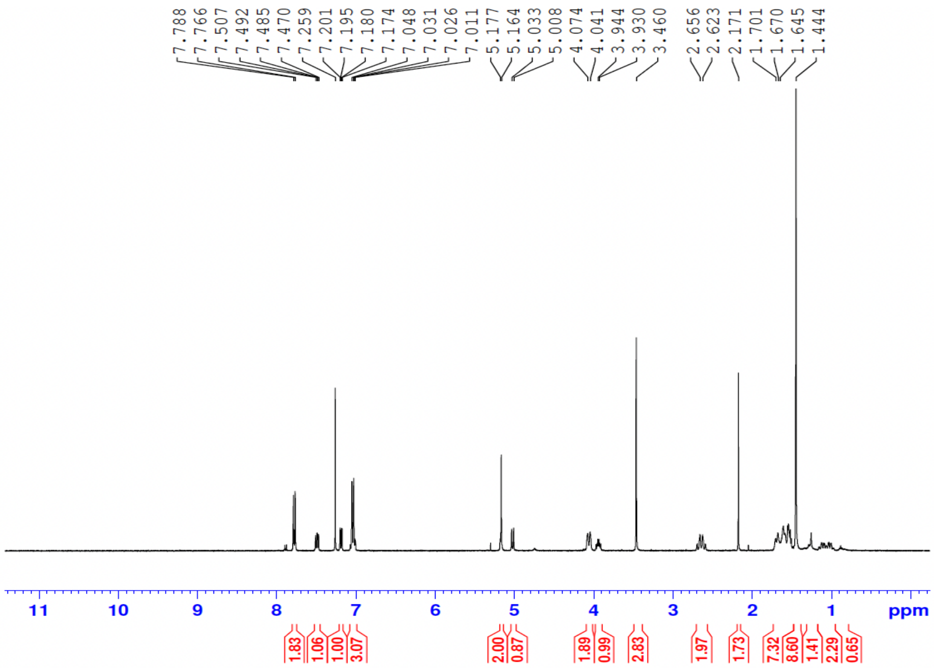

Compound **14**: ^{1}H NMR (400 MHz, DMSO).

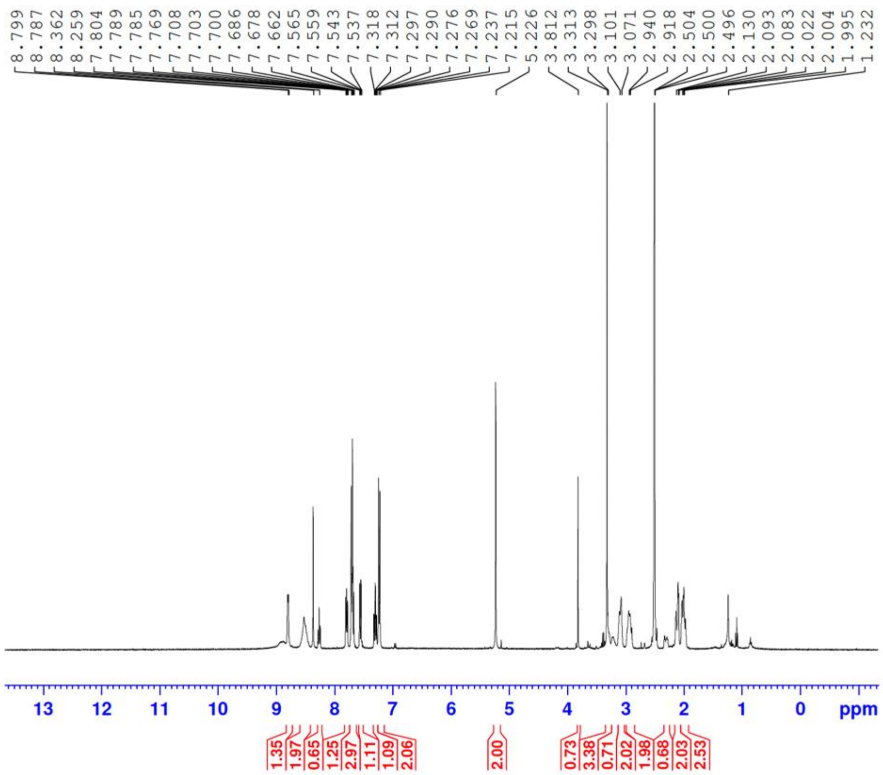

Compound **15**: ^{1}H NMR (400 MHz, MeOD).

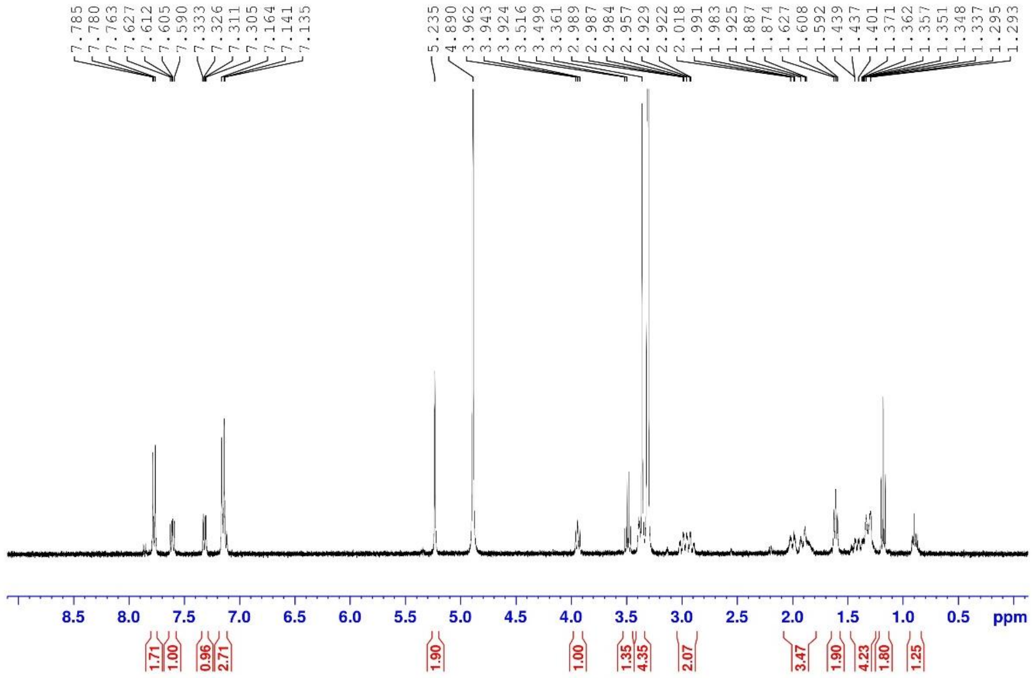

Compound **16**: ^{1}H NMR (400 MHz, $CDCl_3$).

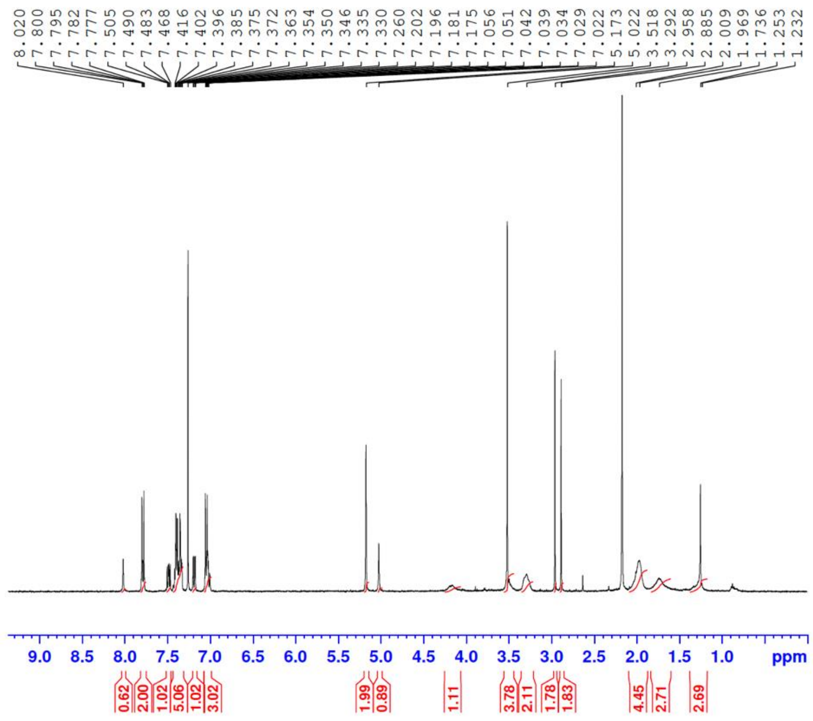

Compound **16**: ^{1}H-^{1}H COSY NMR (400 MHz, $CDCl_3$).

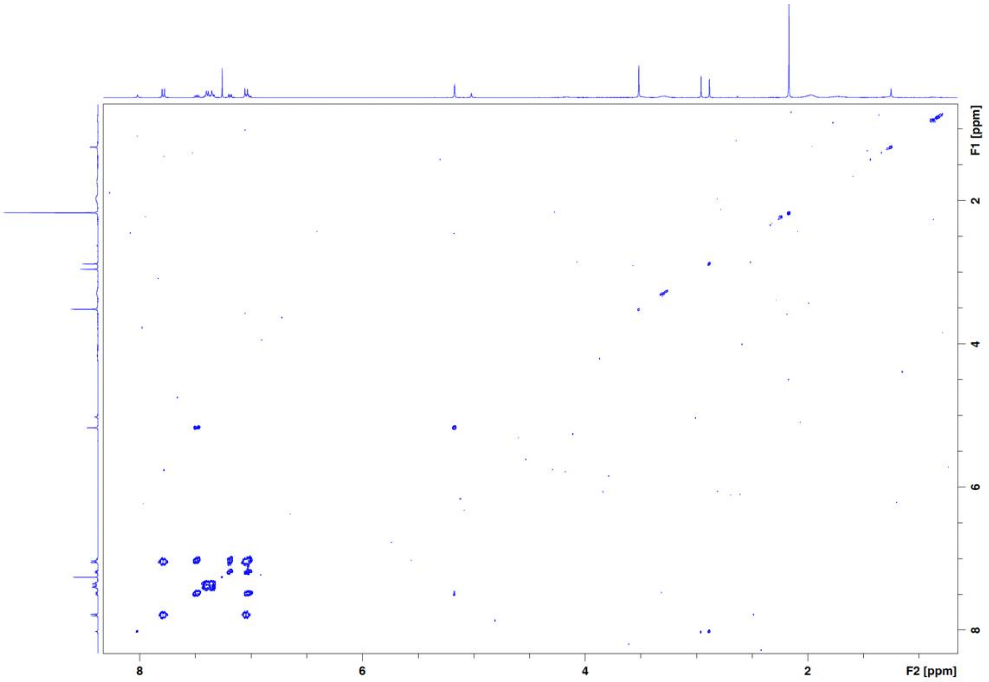

Compound **17**: ^{1}H NMR (400 MHz, $CDCl_3$).

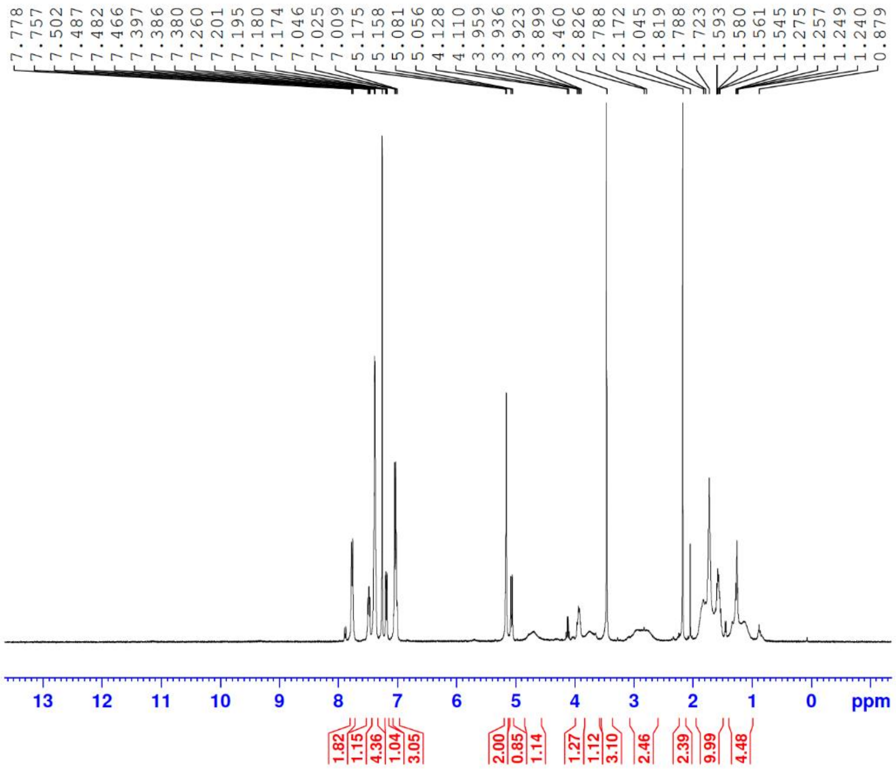

Compound **18**: ^{1}H NMR (400 MHz, DMSO).

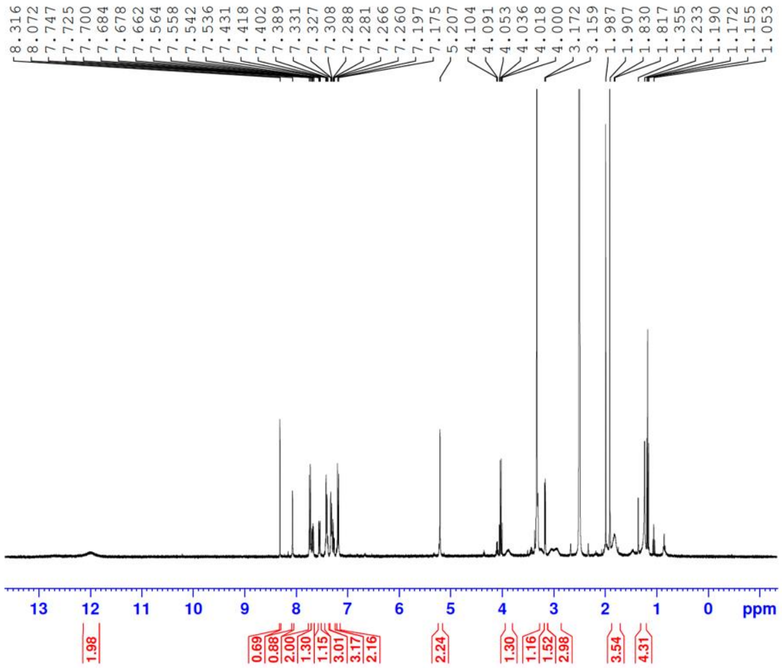

Compound **19**: ^{1}H NMR (400 MHz, MeOD).

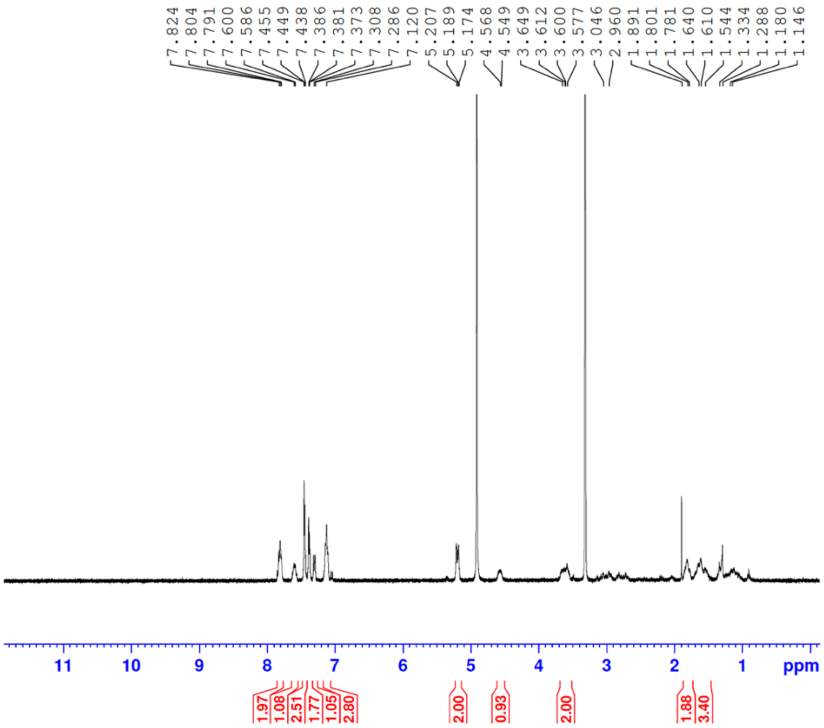

Scheme 3: Synthesis of final compound 3

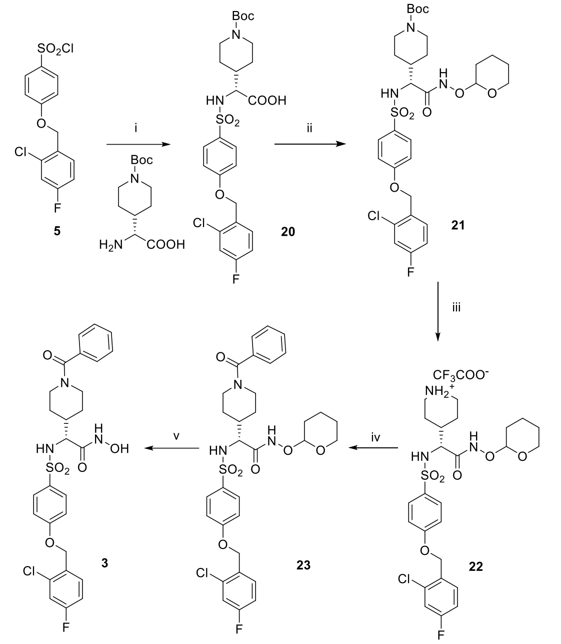

Compound **20**: ^{1}H NMR (400 MHz, DMSO).

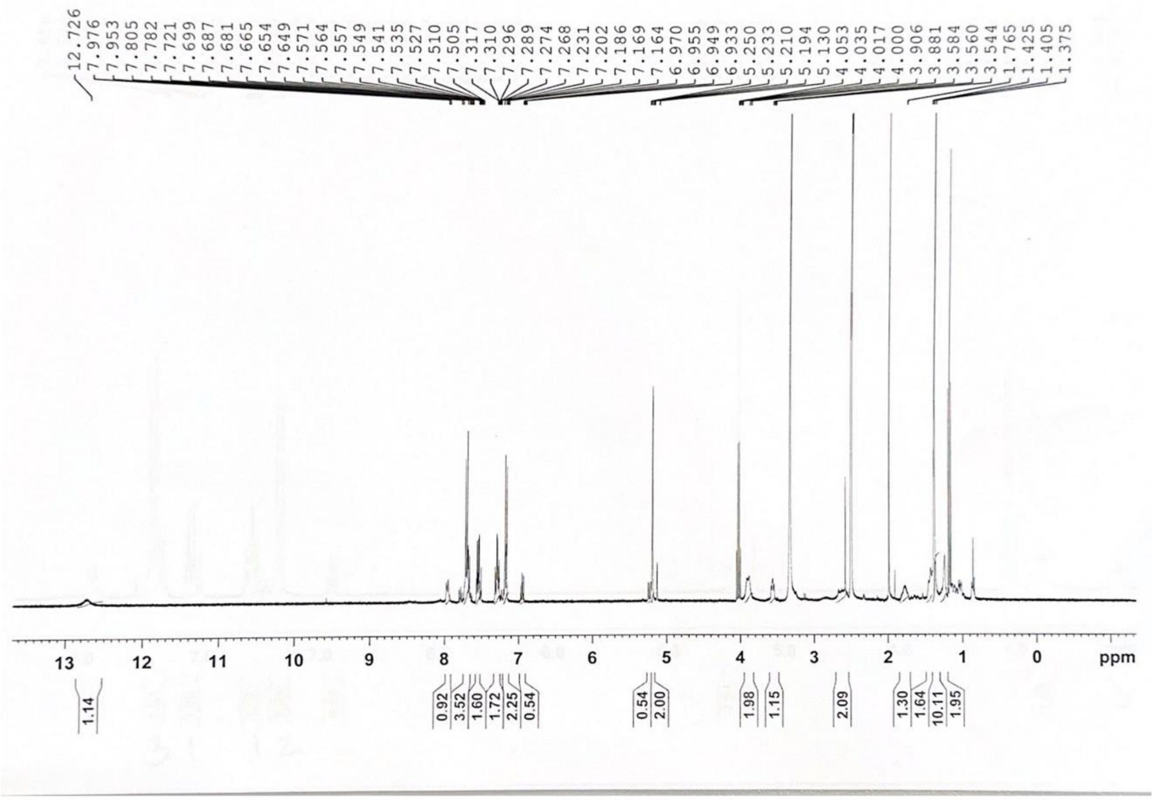

Compound **21**: ^{1}H NMR (400 MHz, $CDCl_3$).

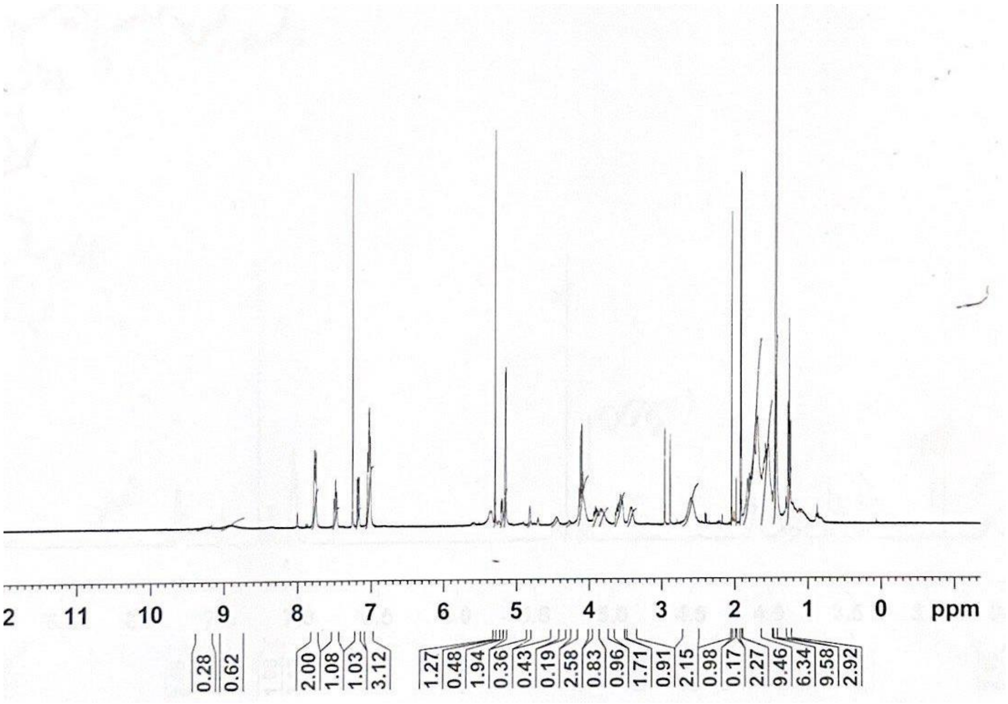

Compound **22**: ^{1}H NMR (400 MHz, MeOD).

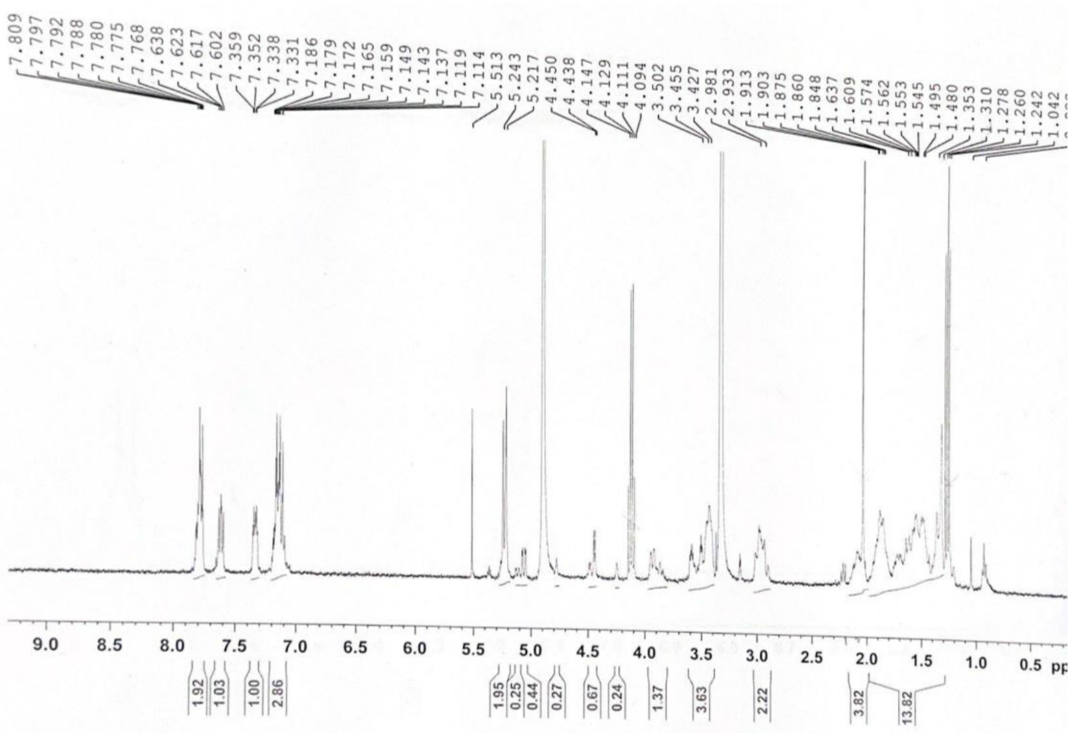

Scheme 4: Synthesis of final compounds 3a-g

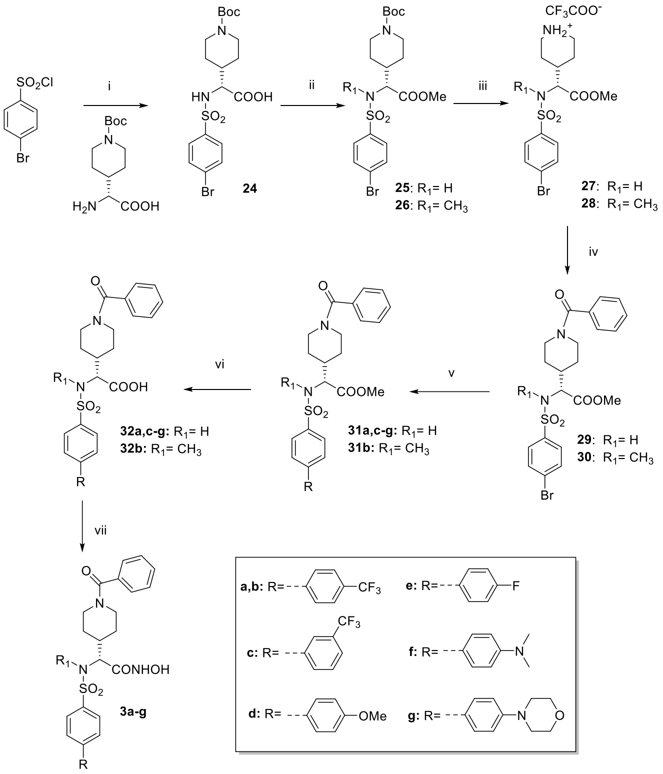

Compound **24**: ^{1}H NMR (400 MHz, $CDCl_3$).

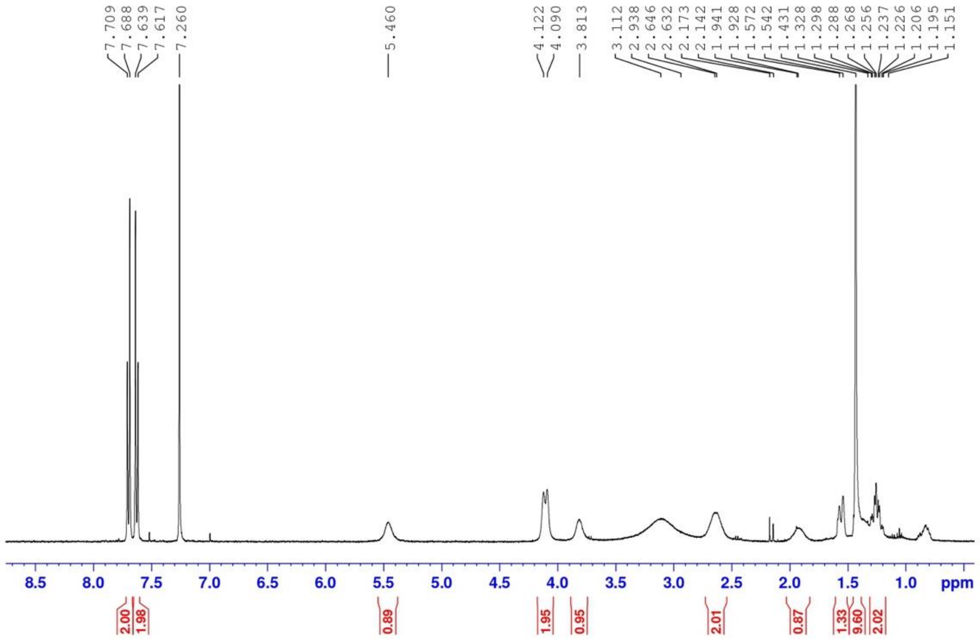

Compound **25**: ^{1}H NMR (400 MHz, $CDCl_3$).

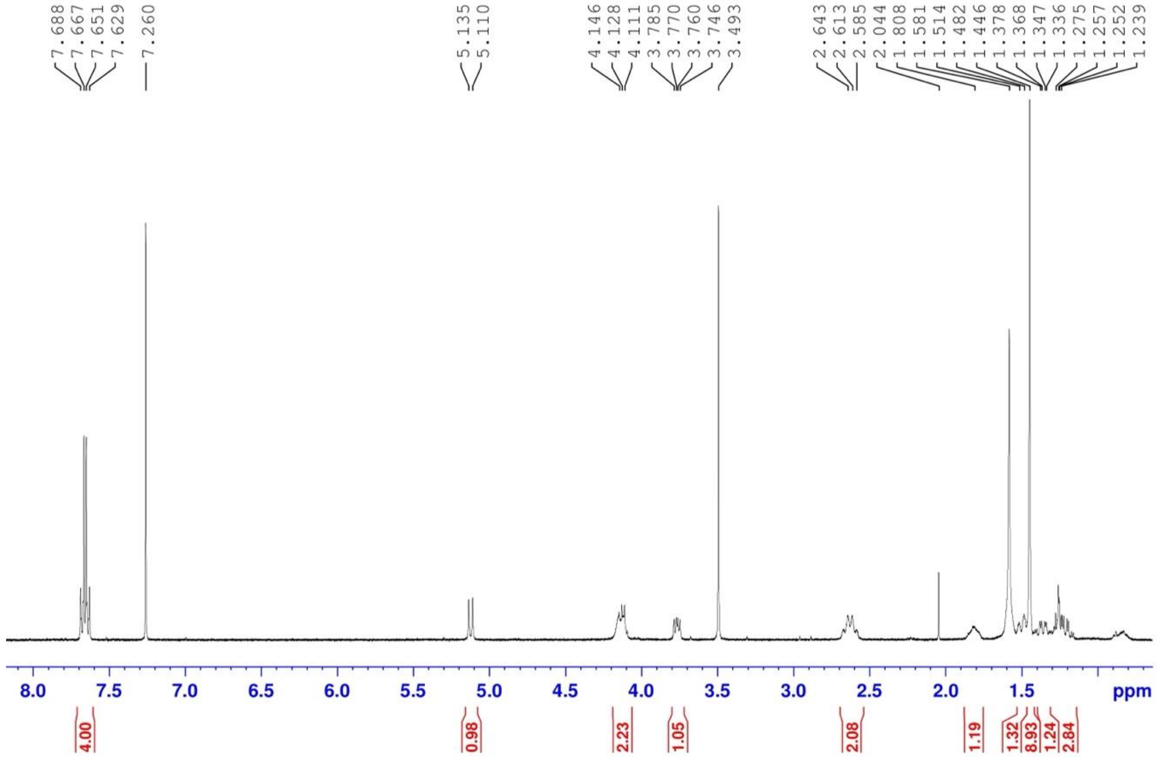

Compound **26**: ^{1}H NMR (400 MHz, $CDCl_3$).

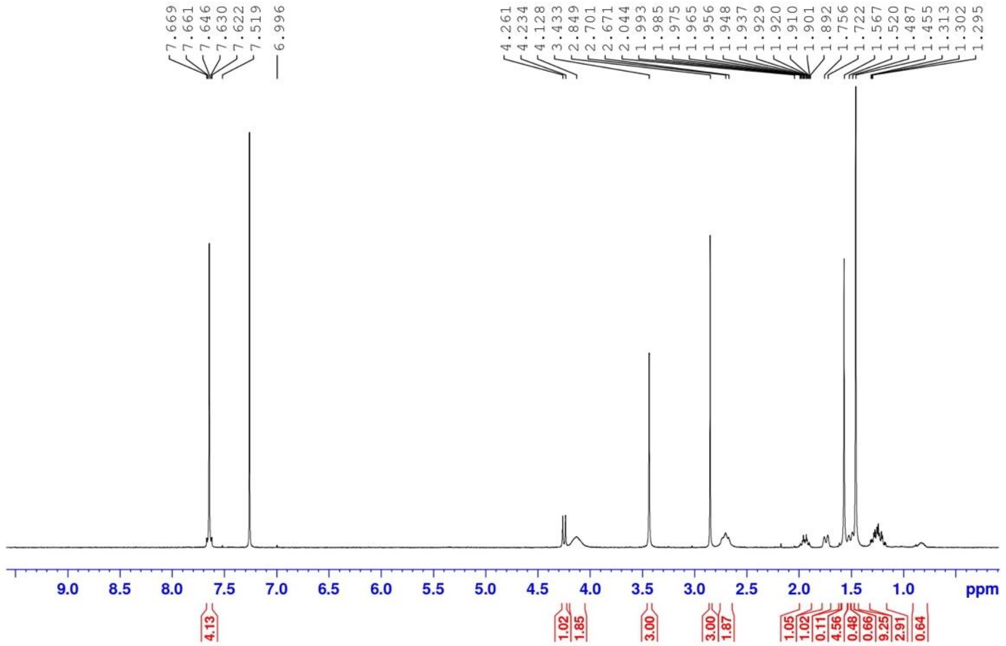

Compound **27**: ^{1}H NMR (400 MHz, $CDCl_3$).

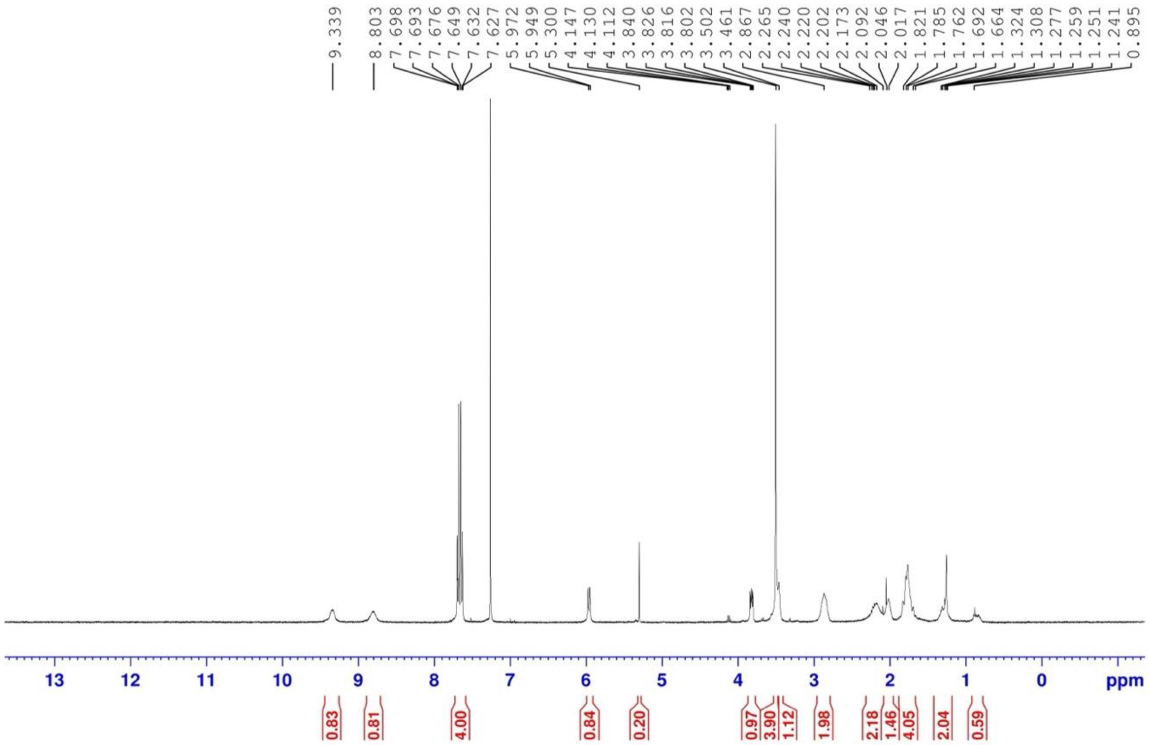

Compound **27**: ^{13}C NMR (400 MHz, $CDCl_3$).

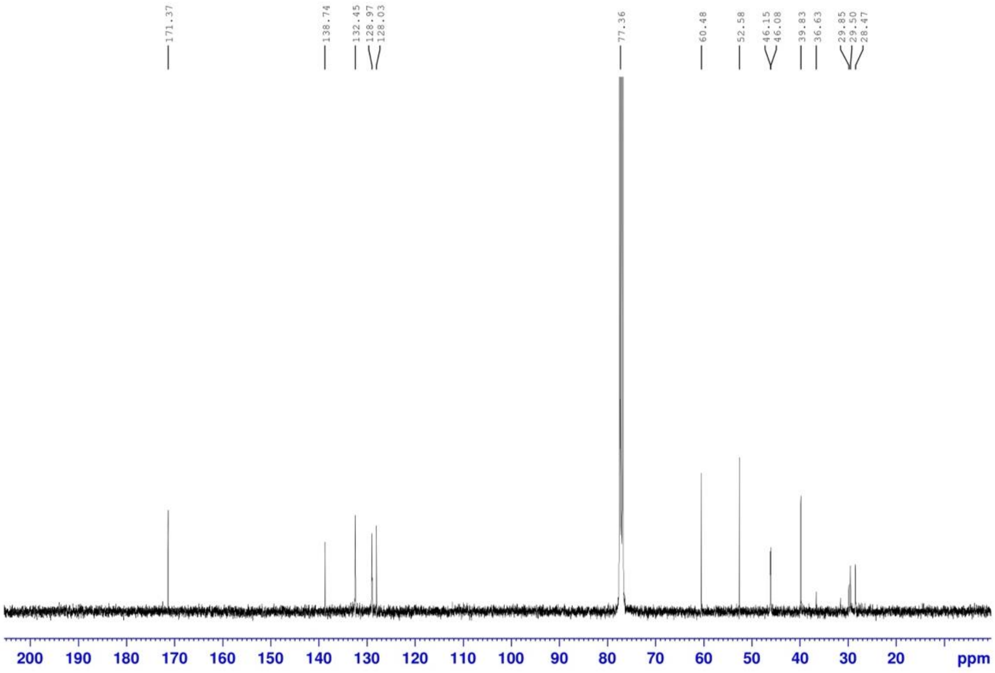

Compound **28**: ^{1}H NMR (400 MHz, $CDCl_3$).

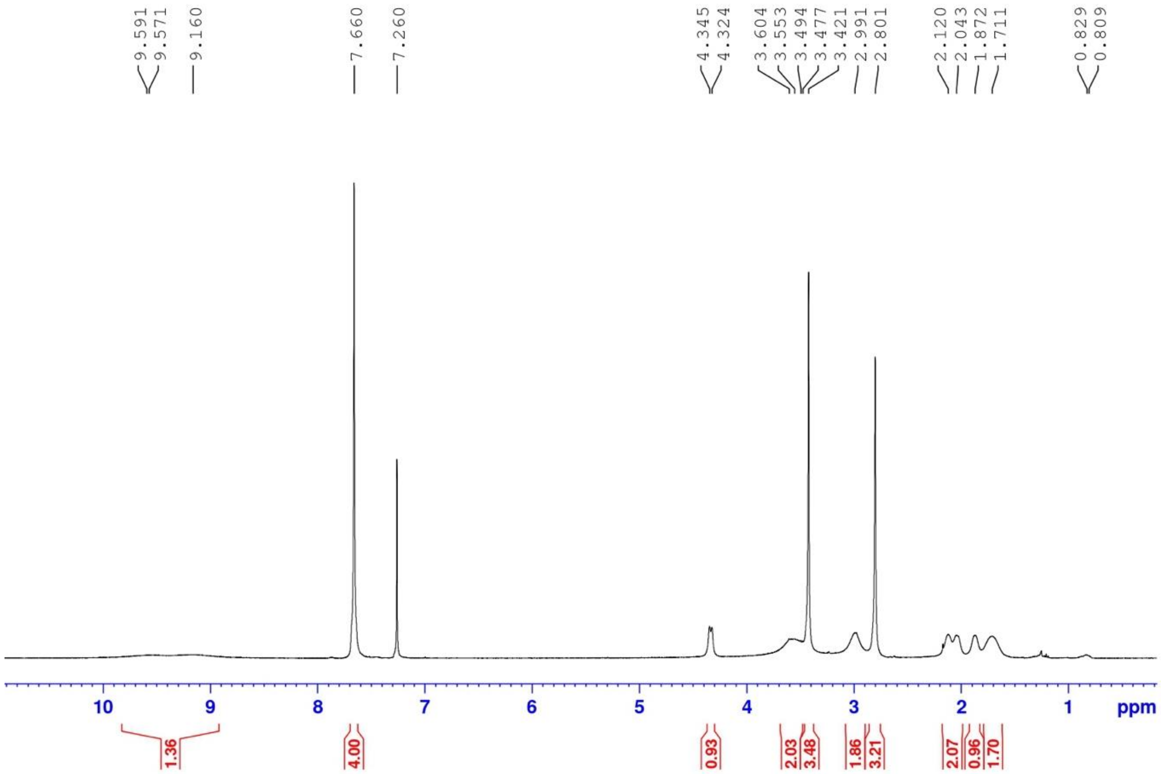

Compound **29**: ^{1}H NMR (400 MHz, $CDCl_3$).

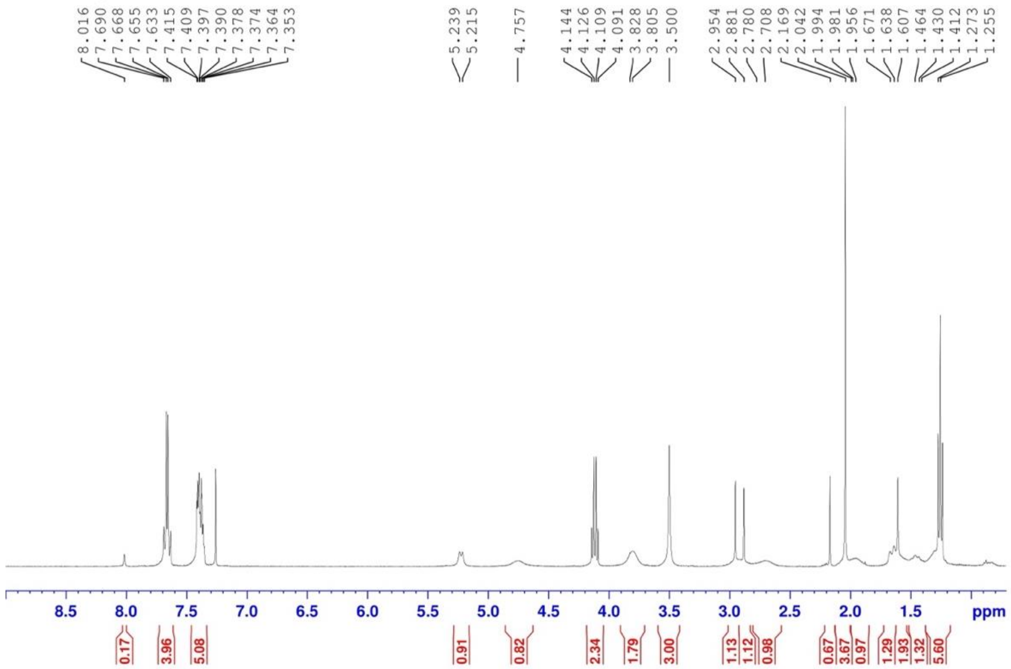

Compound **30:** ^{1}H NMR (400 MHz, $CDCl_3$).

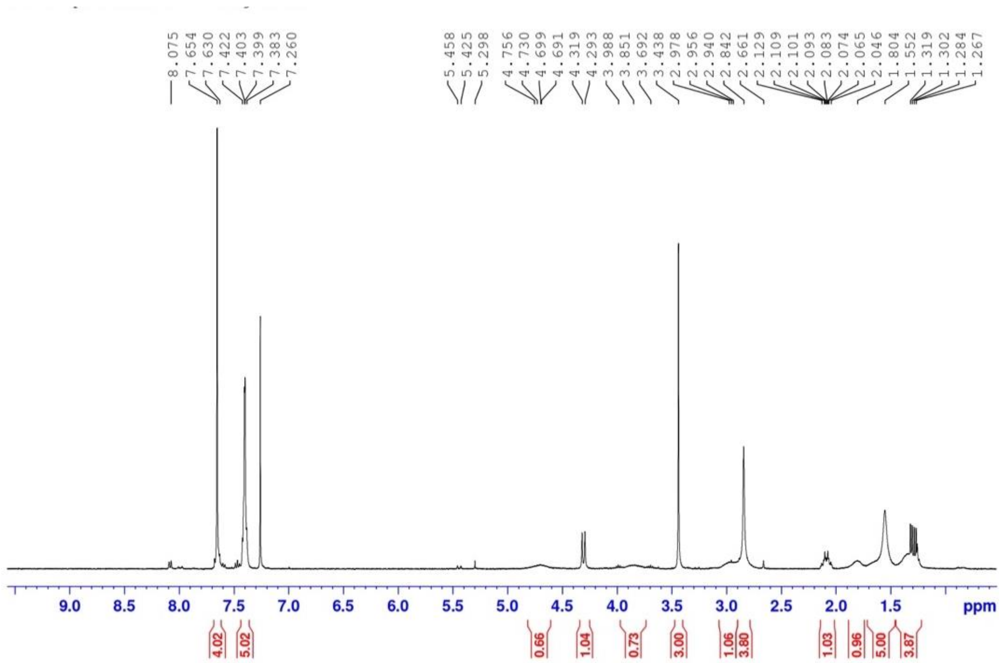

Compound **31a:** ^{1}H NMR (400 MHz, $CDCl_3$).

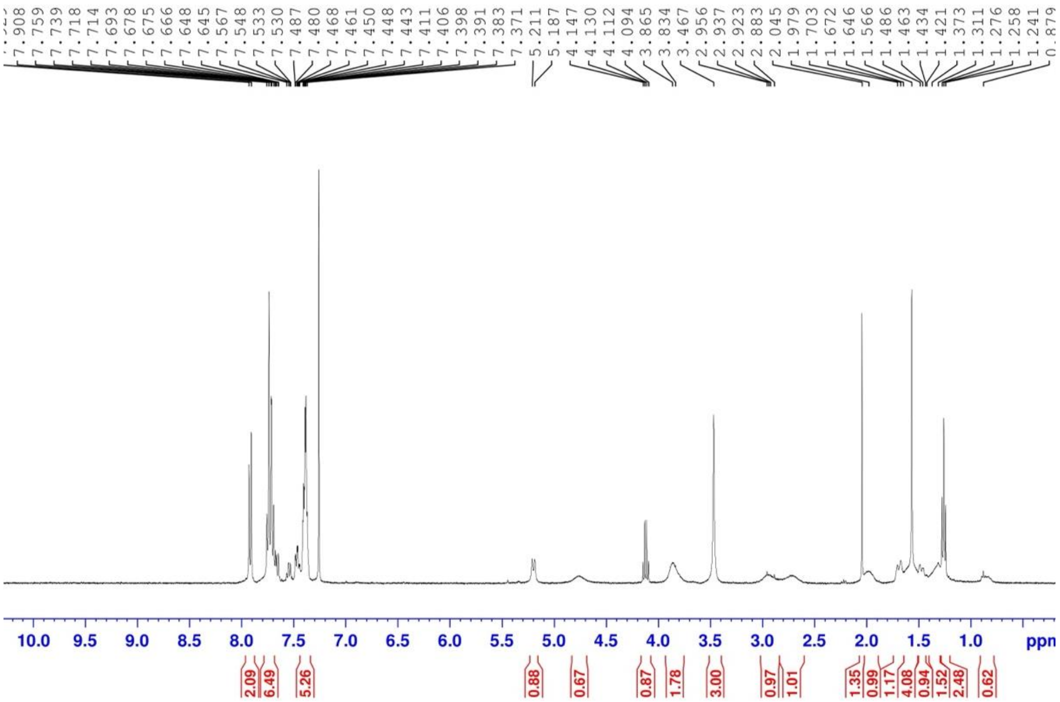

Compound **31b:** ^{1}H NMR (400 MHz, $CDCl_3$).

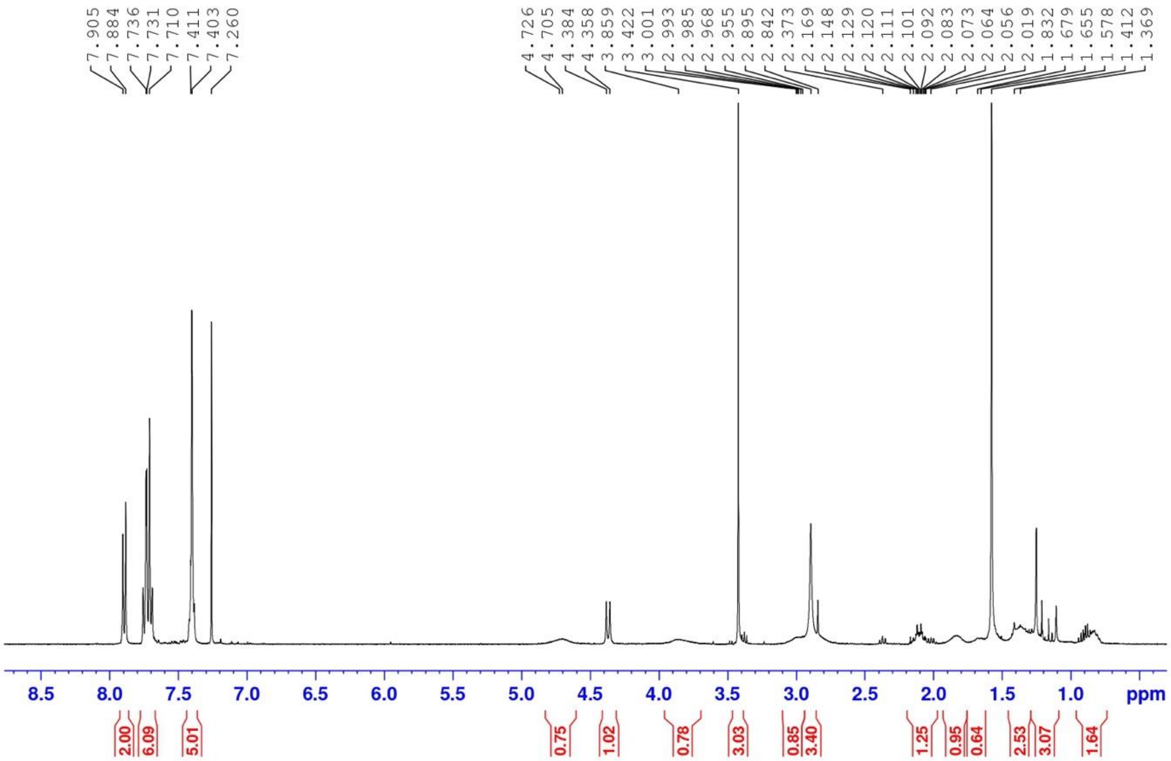

Compound **31c:** ^{1}H NMR (400 MHz, $CDCl_3$).

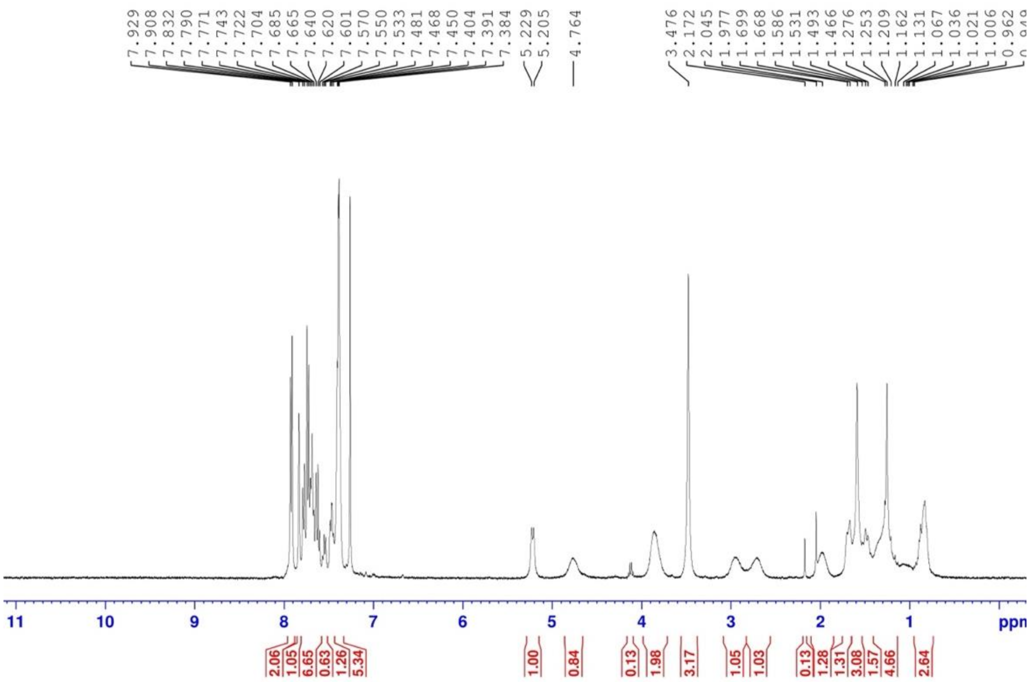

Compound **31d:** ^{1}H NMR (400 MHz, $CDCl_3$).

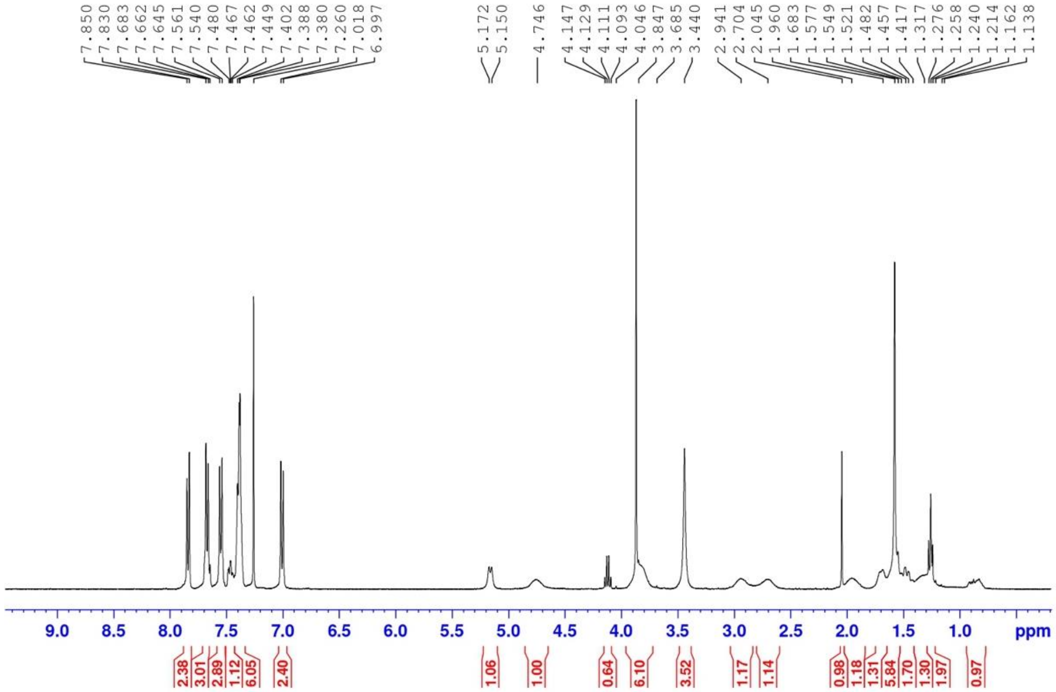

Compound **31e:** ^{1}H NMR (400 MHz, $CDCl_3$).

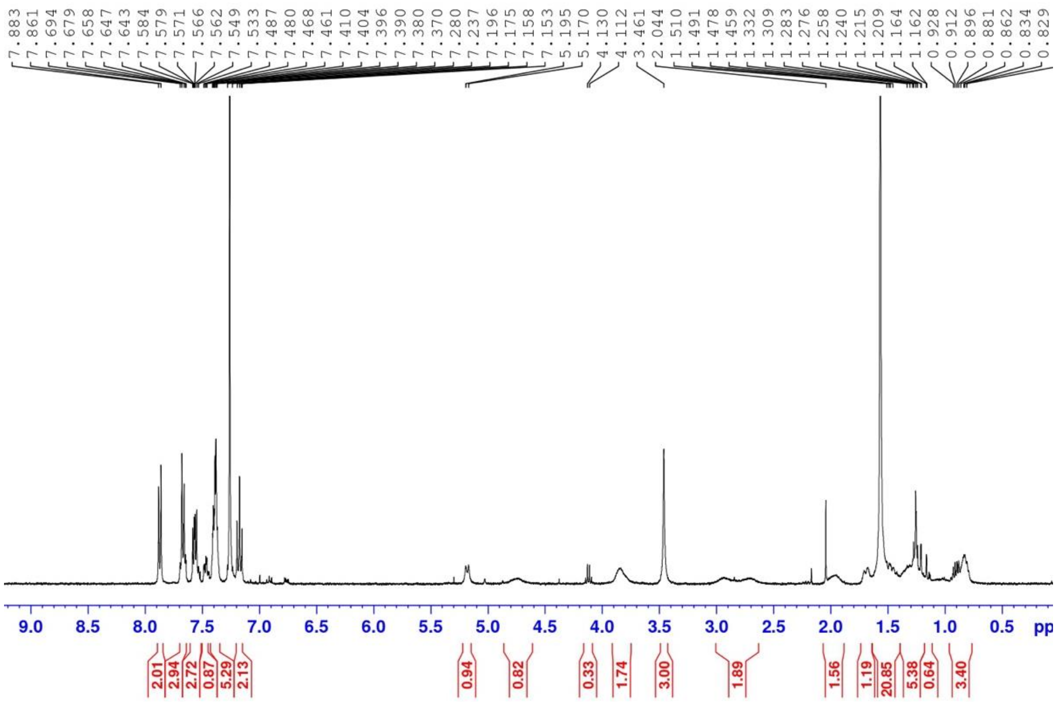

Compound **31f:** ^{1}H NMR (400 MHz, $CDCl_3$).

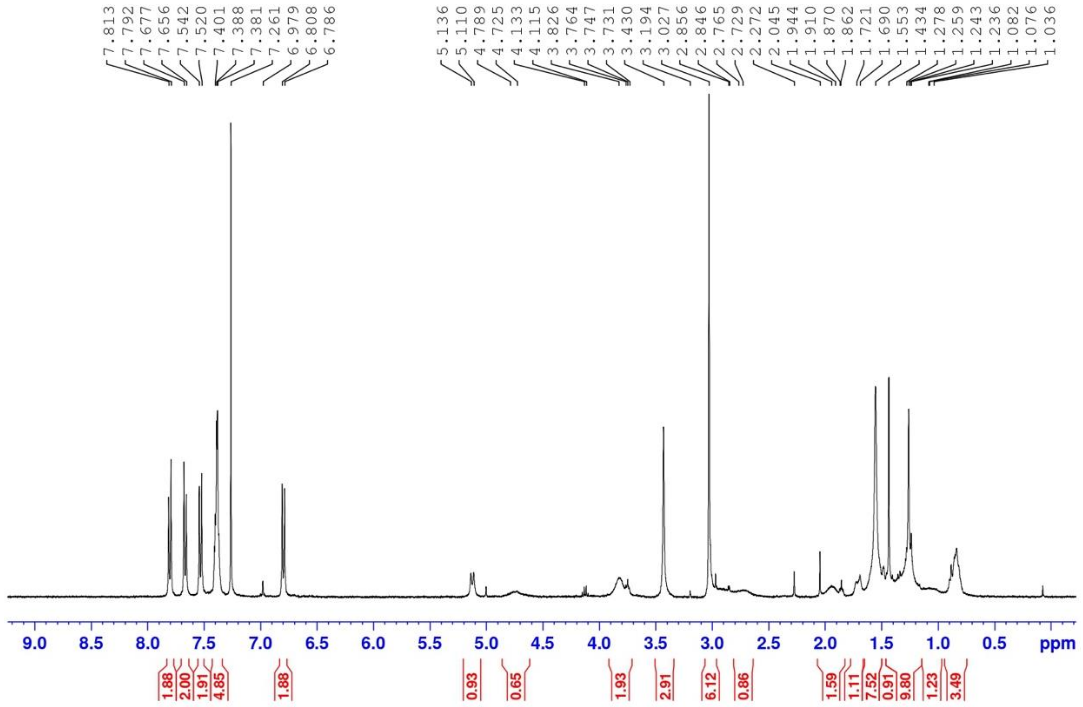

Compound **31g:** ^{1}H NMR (400 MHz, $CDCl_3$).

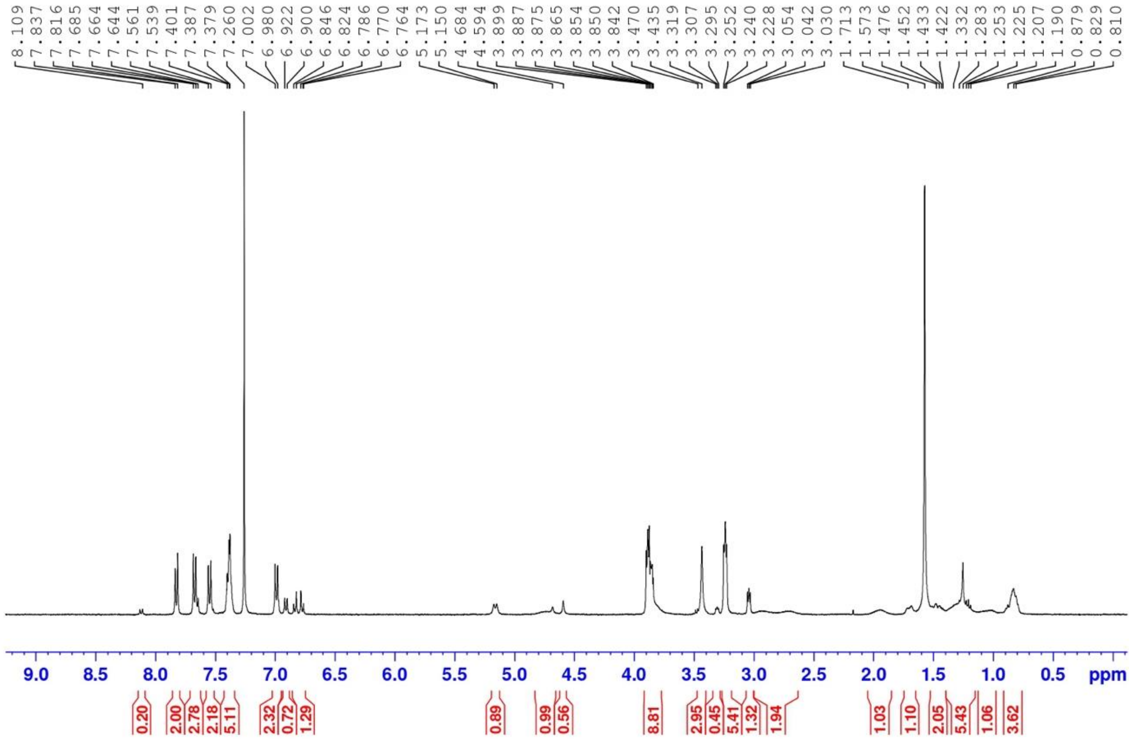

Compound **32a:** ^{1}H NMR (400 MHz, $CDCl_3$).

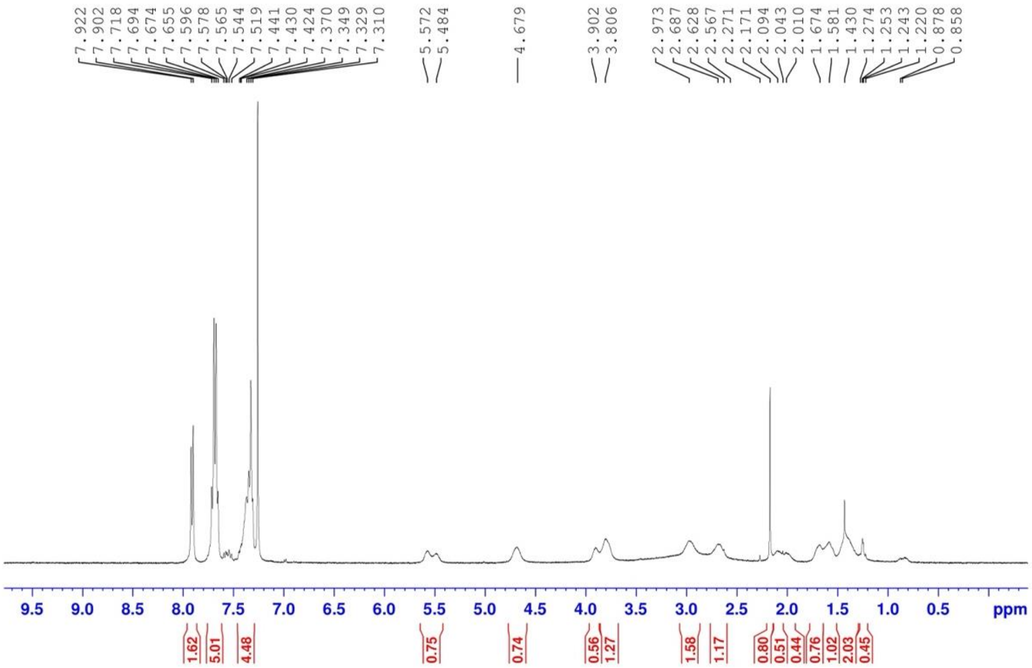

Compound **32b:** ^{1}H NMR (400 MHz, $CDCl_3$).

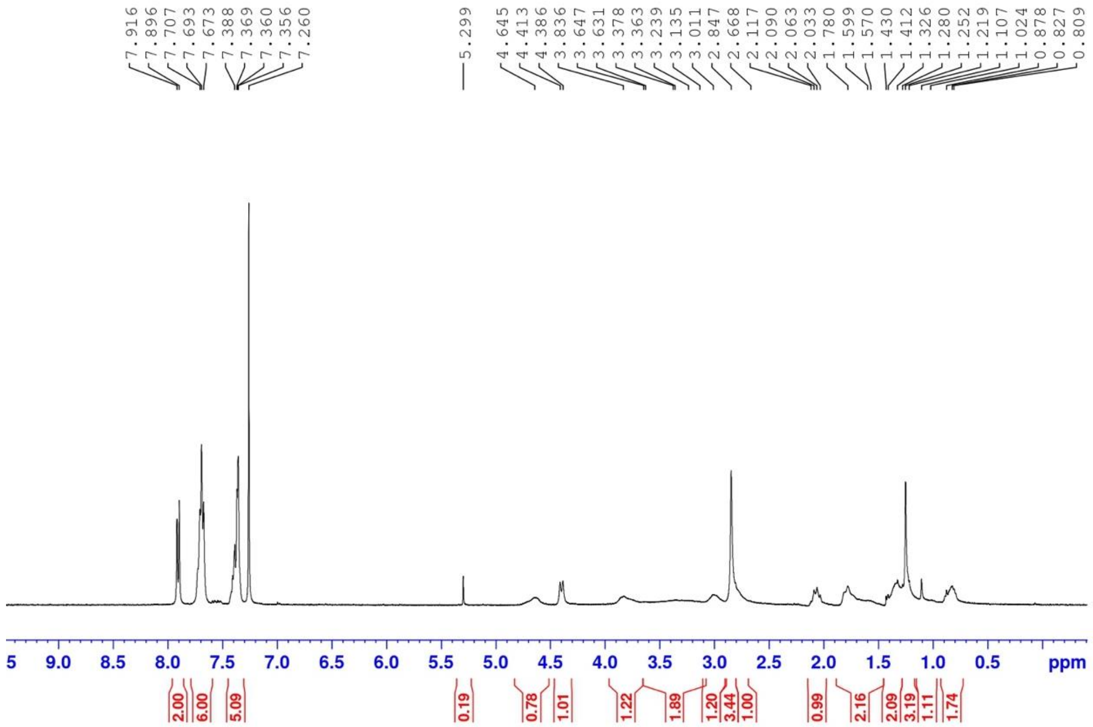

Compound **32c:** ^{1}H NMR (400 MHz, $CDCl_3$).

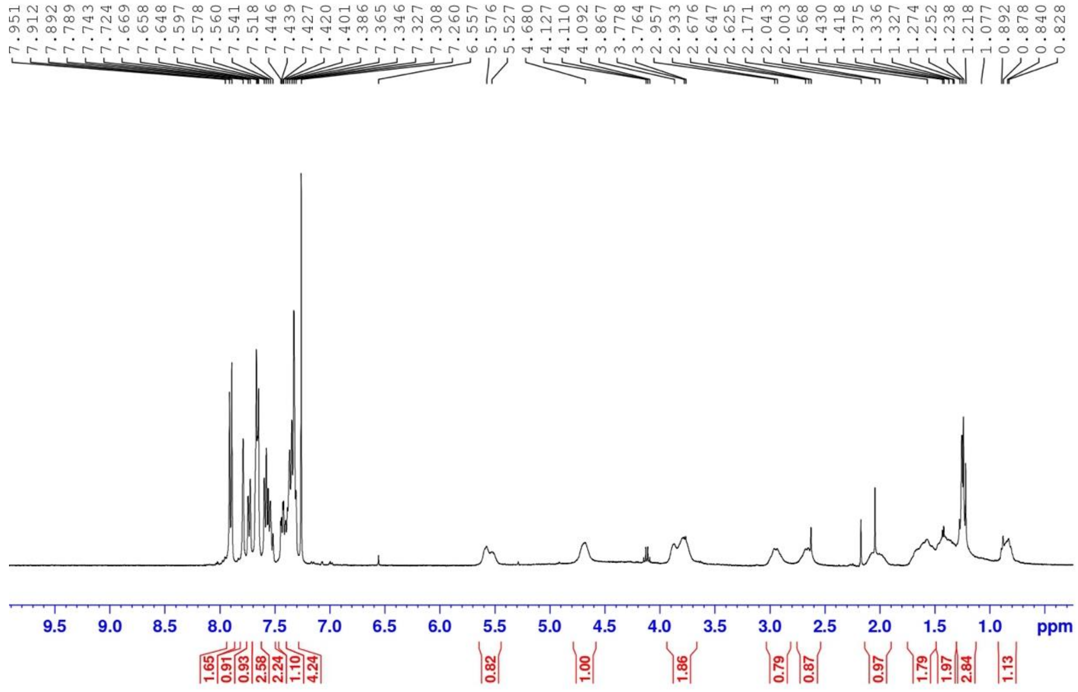

Compound **32c:** ^{13}C NMR (400 MHz, $CDCl_3$).

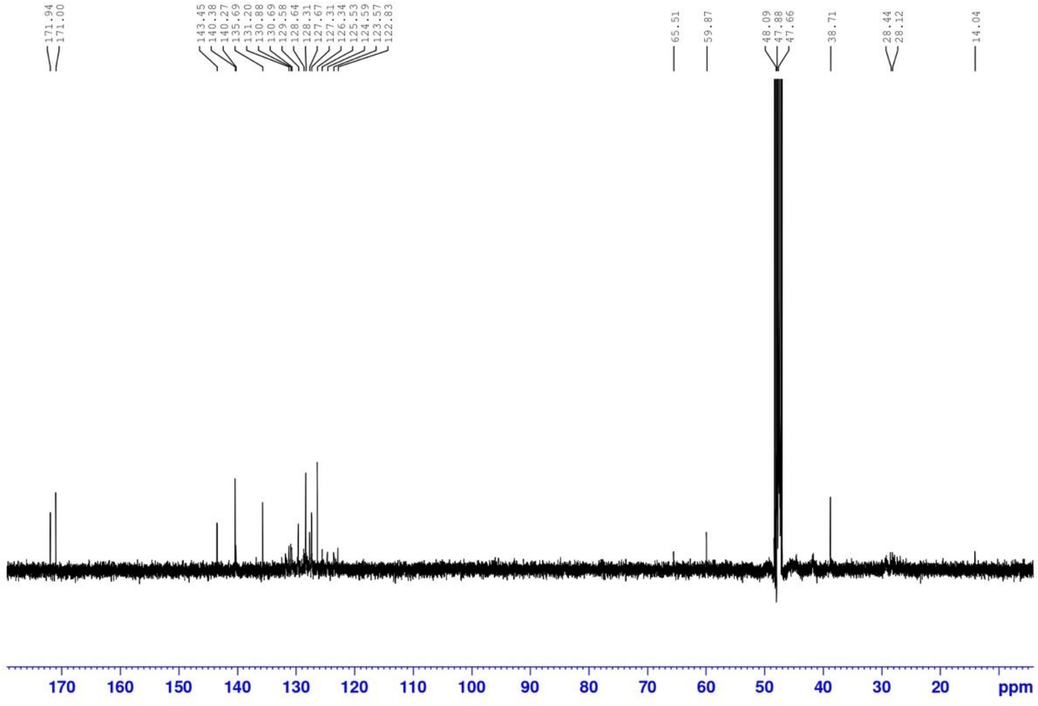

Compound **32d:** ^{1}H NMR (400 MHz, $CDCl_3$).

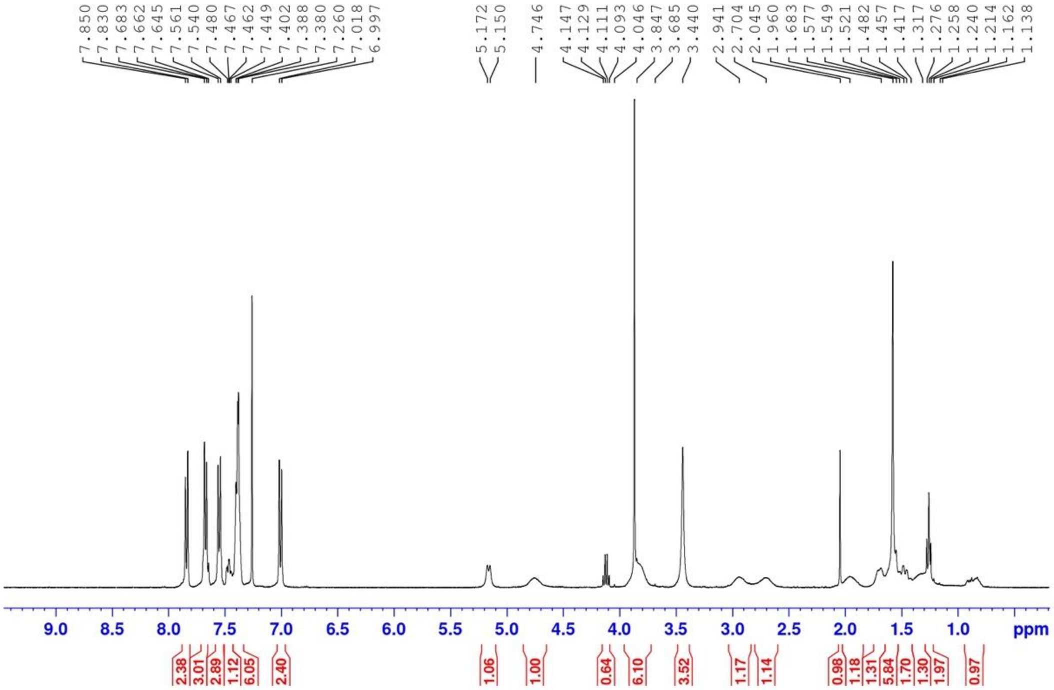

Compound **32e:** ^{1}H NMR (400 MHz, $CDCl_3$).

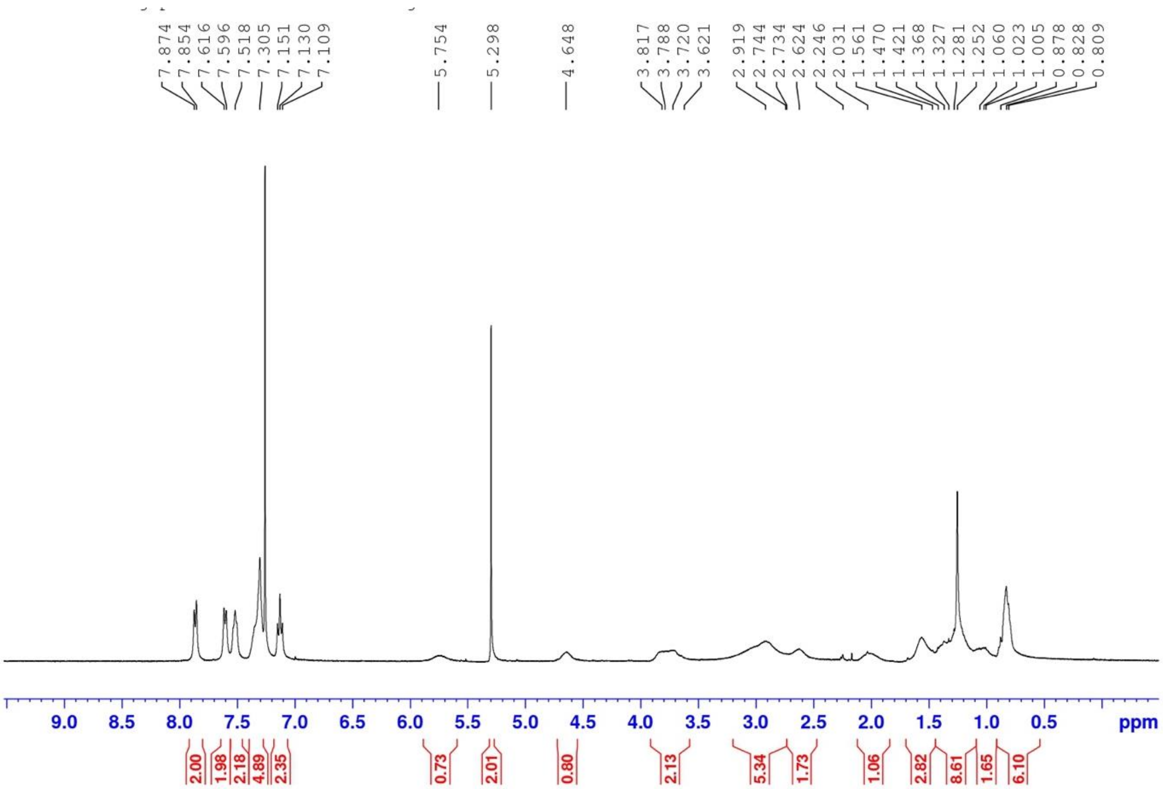

Compound **32f:** ^{1}H NMR (400 MHz, DMSO).

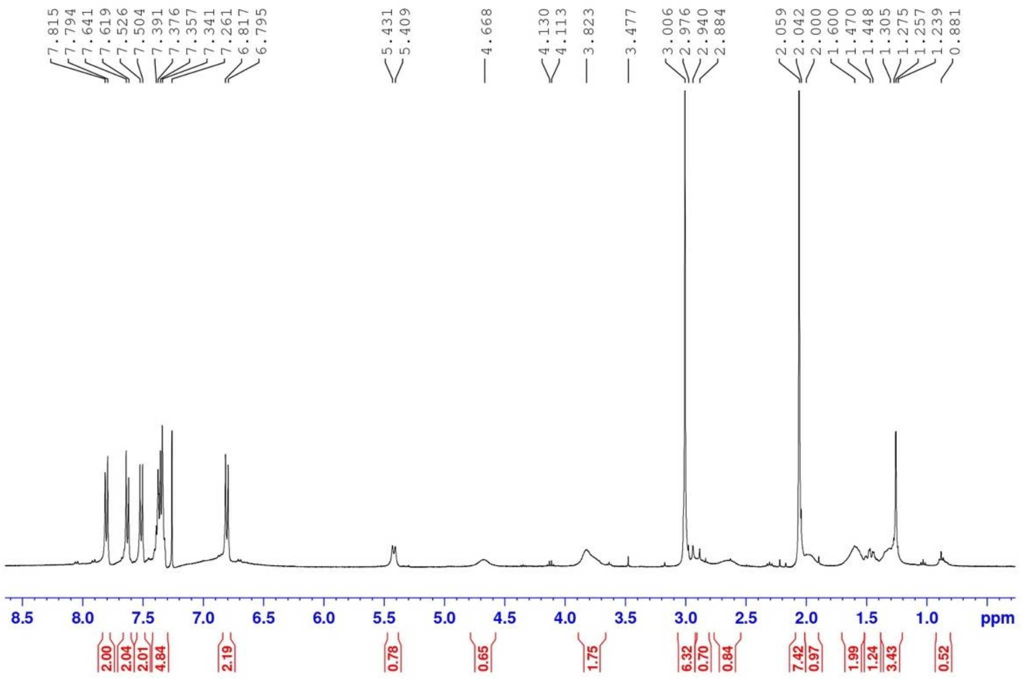

Compound **32g:** ^{1}H NMR (400 MHz, DMSO).

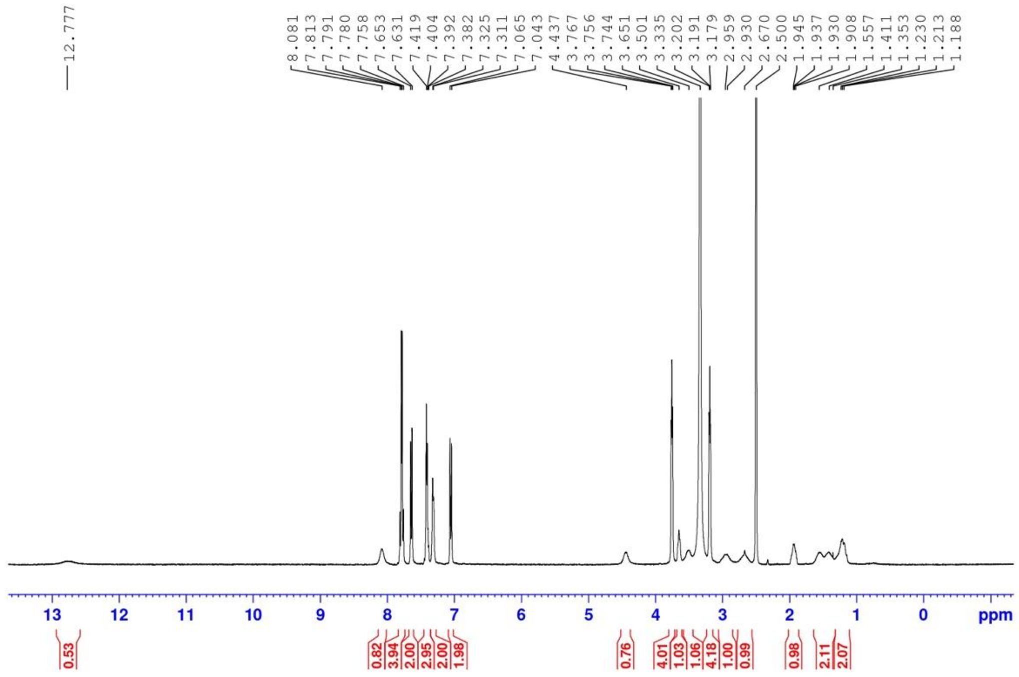

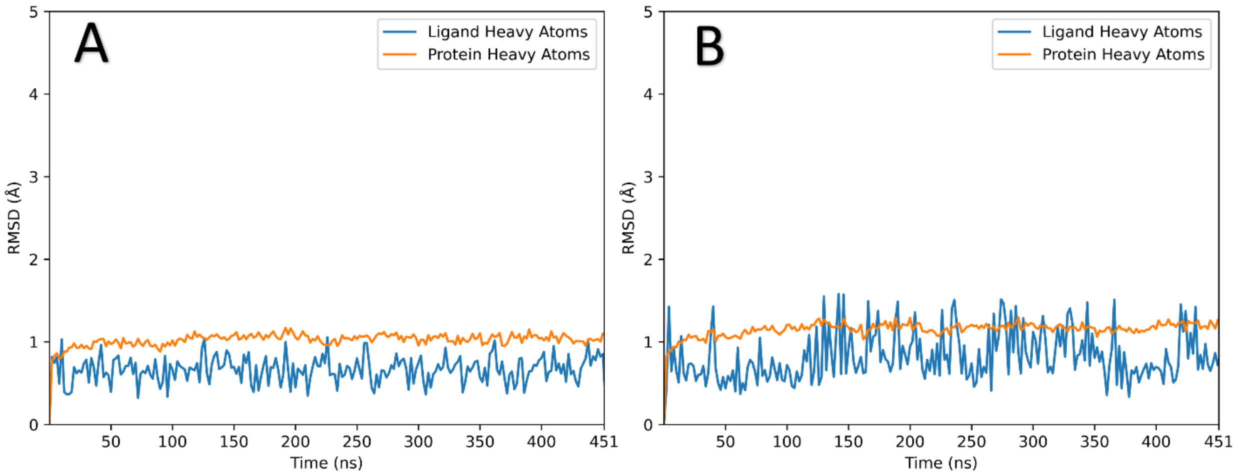

Figure S1. The plot shows the RMSD of **3a** and protein heavy atoms for the ADAMTS5-**3a** (A) and ADAMTS7-**3a** (B) complexes during the MD simulations.

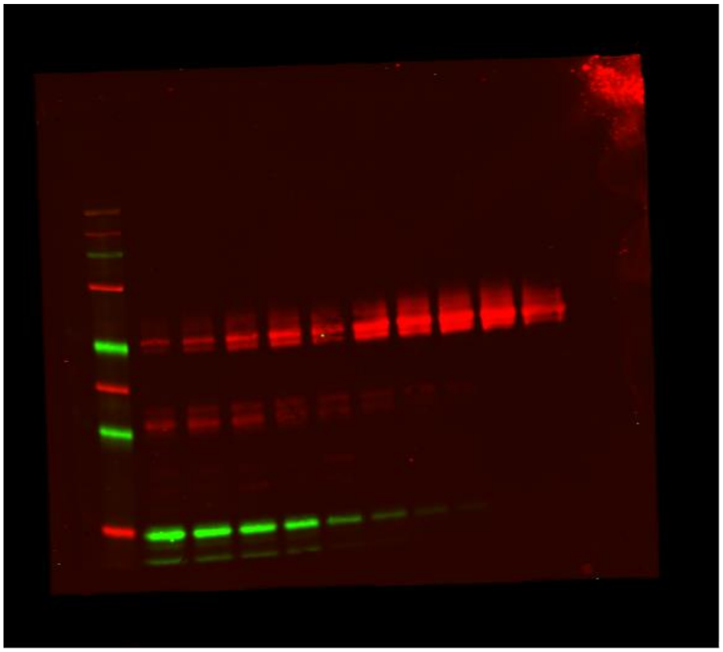

Figure S2. Original Western Blot for the image shown in Figure 2 in the main text. In the latter, the last lane of the original image as shown here was cropped out as it related to a different experiment.
